# Supplementary material for: A randomised controlled trial of a duodenal-jejunal bypass sleeve device (EndoBarrier) compared with standard medical therapy for the management of obese subjects with type 2 diabetes mellitus
Source: BMJ Open. 2017 Nov 15;7(11):e018598. doi: 10.1136/bmjopen-2017-018598 (PMC5695522; doi:10.1136/bmjopen-2017-018598)
Supplement: Author's manuscript [file bmjopen-2017-018598.draft_revisions.pdf]

BMJ Open is committed to open peer review. As part of this commitment we make the peer review history of every article we publish publicly available.

When an article is published we post the peer reviewers' comments and the authors' responses online. We also post the versions of the paper that were used during peer review. These are the versions that the peer review comments apply to.

The versions of the paper that follow are the versions that were submitted during the peer review process. They are not the versions of record or the final published versions. They should not be cited or distributed as the published version of this manuscript.

BMJ Open is an open access journal and the full, final, typeset and author-corrected version of record of the manuscript is available on our site with no access controls, subscription charges or pay-per-view fees (http://bmjopen.bmj.com).

If you have any questions on BMJ Open's open peer review process please email editorial.bmjopen@bmj.com

BMJ Open

Study Protocol: A randomized controlled trial of a duodenal-jejunal bypass sleeve device (EndoBarrier®) compared with standard medical therapy for the management of obese subjects with type 2 diabetes mellitus.

Journal:	*BMJ Open*
Manuscript ID	bmjopen-2017-018598
Article Type:	Protocol
Date Submitted by the Author:	12-Jul-2017

Complete List of Authors:	Glaysher, Michael; University Hospital Southampton, Southamptom Biomedical Research Centre<br>Mohanaruban, Aruchuna; Imperial College Healthcare NHS Trust, St. Mary's Hospital, London, W2 1NY<br>Prechtl, Christina; Imperial College London, Department of Public Health, Imperial Clinical Trials Unit, Stadium House, 68 Wood Lane, London, W12 7RH<br>Goldstone, Anthony; Imperial College London, PsychoNeuroEndocrinology Research Group, Neuropsychopharmacology Unit, Centre for Psychiatry and Computational, Cognitive and Clinical Neuroimaging Laboratory, Division of Brain Sciences, Hammersmith Hospital<br>Miras , Alexander ; Imperial College Healthcare NHS Trust, Division of Diabetes, Endocrinology and Metabolic Medicine, 6th floor Commonwealth Building, Hammersmith Hospital, London W12 0NN<br>Lord, Joanne; University of Southampton, Southampton Health Technology Assessments Centre, University of Southampton, Alpha House, University of Southampton Science Park, Southampton SO16 7NS<br>Chhina, Navpreet; Imperial College Healthcare NHS Trust, Computational Cognitive and Clinical Neuroimaging Group, Burlington Danes Building, Hammersmith Hospital, Imperial College London, Du Cane Road, London, W12 0NN<br>Falaschetti, Emanuela; Imperial College London, Imperial Clinical Trials Unit, Imperial College London, Stadium House, 68 Wood Lane, London, W12 7RH.<br>Johnson, Nicholas; Imperial College London, School of Public Health, 68 Wood Lane, London, W12 7RH<br>Al-Najim, Werd ; Imperial College London, Department of Investigative Medicine, Imperial College London, London, United Kingdom; University College Dublin, Diabetes Complications Research Centre, Conway Institute, School of Medicine and Medical Sciences<br>Smith, Claire; Imperial College London, Imperial Clinical Trials Unit, 68 Wood Lane, London, W12 7RH<br>Li, Jia ; Imperial College London, Division of Computational and Systems Medicine, Department of Surgery and Cancer, Faculty of Medicine, Imperial College London, Alexander Fleming Building, Imperial College Road, South Kensington, London, SW7 2AZ<br>Patel, Mayank; University Hospital Southampton, Department of Diabetes and Endocrinology, Room CE145, Mailpoint 134, Southampton General Hospital, Tremona Road, Southampton, SO16 6DD

	Ahmed, Ahmed; Imperial College Healthcare NHS Trust, Consultant Upper GI and Bariatric Surgeon, Clinical Lead – Bariatric Surgery, St Mary’s Hospital, Imperial College Healthcare NHS Trust, Room 35, 4th Floor, Salton House, St Mary’s Hospital, Praed Street, London, W2 1NY.<br>Moore, Michael; University of Southampton Medical School, Primary Care Medical Group<br>Poulter, Neil; Imperial College London, Imperial Clinical Trials Unit, 1st Floor Stadium House, 68 Wood Lane, London W12 7TA<br>Bloom, Stephen; Imperial College London, Clinical Director, North West London Pathology, Head of Division of Diabetes, Endocrinology and Metabolism, Imperial College, 6th Floor Commonwealth Building, Hammersmith Hospital, Imperial College London, Du Cane Road, London, W12 0NN<br>Darzi, Ara; Imperial College London, Institute of Global Health Innovation<br>Le Roux, Carel; University College Dublin<br>Byrne, James; University Hospital Southampton, Division of Surgery, University Hospital Southampton NHS Foundation Trust, Tremona Road, Southampton, Hampshire, SO16 6YD<br>Teare, Julian; Imperial College Healthcare NHS Trust, Department of Gastroenterology, St. Mary's Hospital, Praed Street, London, W2 1NY
**Primary Subject Heading**:	Diabetes and endocrinology
Secondary Subject Heading:	Nutrition and metabolism, Surgery
Keywords:	Obesity, Type 2 diabetes mellitus, Endobarrier, Duodenal-jejunal bypass sleeve, Duodenal-jejunal bypass liner

SCHOLARONE™
Manuscripts

Study Protocol: A randomized controlled trial of a duodenal-jejunal bypass sleeve device (EndoBarrier®) compared with standard medical therapy for the management of obese subjects with type 2 diabetes mellitus.

Authors:

Mr Michael A Glaysher
michaelglaysher@me.com
Clinical Research Fellow and General Surgical Registrar, Level D, Laboratory & Pathology Block, SCBR - MP 218, University Hospital Southampton NHS Foundation Trust, Tremona Road, Southampton, Hampshire, SO16 6YD. Tel: 023 8120 5339 Mob: 07834897272
No funding or conflicts of interest to declare

Dr Aruchuna Mohanaruban
a.mohanaruban@imperial.ac.uk
Clinical research fellow and gastroenterology registrar, Imperial College Healthcare NHS Trust, St. Mary's Hospital, London, W2 1NY
No funding or conflicts of interest to declare

Dr Christina Gabriele Prechtl
c.prechtl@imperial.ac.uk
Imperial College London, Department of Public Health, Imperial Clinical Trials Unit, Stadium House, 68 Wood Lane, London, W12 7RH. Phone: +44 207 594 5946
Funding: National Institute for Health Research, No conflict of interest

Dr. Anthony P. Goldstone
tony.goldstone@imperial.ac.uk
PsychoNeuroEndocrinology Research Group, Neuropsychopharmacology Unit, Centre for Psychiatry and Computational, Cognitive and Clinical Neuroimaging Laboratory, Division of Brain Sciences, Imperial College London, Hammersmith Hospital, London, UK. Tel: + 44 20 7594 5989
No competing interests. Funding supported by UK Medical Research Council and Wellcome Trust

Dr Alexander Dimitri Miras
a.miras@nhs.net
Senior Clinical Lecturer in Endocrinology, Division of Diabetes, Endocrinology and Metabolic Medicine, 6th floor Commonwealth Building, Hammersmith Hospital, London W12 0NN. Tel: +44 (0)7958 377674. Fax: +44 (0)208 3838320
Funding: MRC, NIHR, Moulton Charitable Foundation, Fractyl, Novo Nordisk, Astra Zeneka

Professor Joanne Lord
j.lord@soton.ac.uk
Southampton HTA Centre, University of Southampton, Alpha House, University of Southampton Science Park, Southampton SO16 7NS. Tel: +44(0)23 8059 3749
Prof. Lord reports grants from Efficacy and Mechanism Evaluation (EME) Programme, an MRC and National Institute for Health Research (NIHR) partnership, and non-financial support from GI Dynamics Inc , during the conduct of the study.

Dr Navpreet Chhina
navpreet.chhina@imperial.ac.uk
Clinical Research Fellow in Endocrinology, Computational Cognitive and Clinical Neuroimaging Group, Burlington Danes Building, Hammersmith Hospital, Imperial College London, Du Cane Road, London, W12 0NN. Tel: 020 7594 5988
No funding or conflicts of interest to declare

For peer review only

Emanuela Falaschetti, CStat
e.falaschetti@imperial.ac.uk
Research Fellow in Clinical Trial Statistics, Imperial Clinical Trials Unit, Imperial College London, Stadium House, 68 Wood Lane, London, W12 7RH. Tel: 020 75941846
No funding or conflicts of interest to declare

Mr Nicholas Andrew Johnson
nicholas.johnson@imperial.ac.uk
Imperial College London, School of Public Health, 68 Wood Lane, London, W12 7RH. Tel: 020 7594 1751
No funding or conflicts of interest to declare

Dr Werd Al-Najim
w.al-najim@imperial.ac.uk
*Department of Investigative Medicine, Imperial College London, London, United Kingdom. Tel: 07590 427 587
*Diabetes Complications Research Centre, Conway Institute, School of Medicine and Medical Sciences, University College Dublin, Ireland
Dr. Al-Najim reports grants from Efficacy and Mechanism Evaluation (EME) Programme, an MRC and National Institute for Health Research (NIHR) partnership. Other support was provided from GI Dynamics.

Dr Claire Smith
claire.smith@imperial.ac.uk
Imperial College London, Imperial Clinical Trials Unit, 68 Wood Lane, London, W12 7RH. Tel: 020 7594 2101.
No funding or conflicts of interest to declare

Dr Jia V Li
jia.li@imperial.ac.uk
Division of Computational and Systems Medicine, Department of Surgery and Cancer, Faculty of Medicine, Imperial College London, Alexander Fleming Building, Imperial College Road, South Kensington, London, SW7 2AZ, UK. Phone: 02075943230
No funding or conflicts of interest to declare

Dr Mayank Patel
Mayank.Patel@uhs.nhs.uk
Consultant in Diabetes & Endocrinology, Room CE145, Mailpoint 134, Southampton General Hospital, Tremona Road, Southampton, SO16 6DD. Tel: 023 8120 4165
No funding or conflicts of interest to declare

Mr Ahmed R Ahmed
Ahmed.ahmed@imperial.nhs.uk
Consultant Upper GI and Bariatric Surgeon, Clinical Lead – Bariatric Surgery, St Mary's Hospital, Imperial College Healthcare NHS Trust, Room 35, 4th Floor, Salton House, St Mary's Hospital, Praed Street, London, W2 1NY. Tel: +44 20 8846 1081
No funding or conflicts of interest to declare

Professor Michael Moore
Mvm198@soton.ac.uk
Primary Care and Population Sciences, University of Southampton, Aldermoor Health Centre, Aldermoor Close, Southampton, Hampshire, SO16 5ST. Tel: 023 8059 1774
No funding or conflicts of interest to declare

Prof Neil Reginald Poulter

For peer review only

n.poulter@imperial.ac.uk
Imperial College London, Imperial Clinical Trials Unit, 1st Floor Stadium House, 68 Wood Lane, London W12 7TA. Tel: +44 (0)207 594 3446
Prof. Poulter's institution reports grants from HTA, during the conduct of the study.

Professor Sir Stephen Bloom
s.bloom@imperial.ac.uk
Clinical Director, North West London Pathology, Head of Division of Diabetes, Endocrinology and Metabolism, Imperial College, 6th Floor Commonwealth Building,, Hammersmith Hospital, Imperial College London, Du Cane Road, London, W12 0NN. Tel: 020 8383 3242.
No funding or conflicts of interest to declare

Professor Ara Darzi
a.darzi@imperial.ac.uk
Paul Hamlyn Chair of Surgery, Imperial College London, Division of Surgery, 10th Floor QEQM, St Mary's Hospital, South Wharf Road, London, W2 1NY. Tel: 020 3312 1310
No funding or conflicts of interest to declare

Prof Carel Le Roux
carel.leroux@ucd.ie
Imperial College London, Charing Cross Hospital, Fulham Palace Rd, London W6 8RF. Tel: +44 (0)7970 719 453
Dr. le Roux reports grants from Science Foundation Ireland, grants from Health Research Board, during the conduct of the study; other from NovoNordisk, other from GI Dynamics, personal fees from Eli Lilly, grants and personal fees from Johnson and Johnson, personal fees from Sanofi Aventis, personal fees from Astra Zeneca, personal fees from Janssen, personal fees from Bristol-Myers Squibb, personal fees from Boehringer-Ingelheim, outside the submitted work; .

Mr James P Byrne
James.byrne@uhs.nhs.uk
Division or Surgery, University Hospital Southampton NHS Foundation Trust, Tremona Road, Southampton, Hampshire, SO16 6YD. Tel: 023 8120 8535.
No funding or conflicts of interest to declare

Prof Julian Teare
j.teare@imperial.ac.uk
Imperial College Healthcare NHS Trust, Clarence Wing, St. Mary's Hospital, London, W2 1NY. Tel: +44 (0)20 3312 1072

For peer review only

Abstract

Introduction: The prevalence of obesity and obesity-related diseases, including type 2 diabetes mellitus (T2DM) is increasing. Exclusion of the foregut, as occurs in Roux-en-Y gastric bypass, has a key role in the metabolic improvements that occur following bariatric surgery, which are independent of weight-loss. Endoscopically-placed duodenal-jejunal bypass sleeve (DJBS) devices, such as the EndoBarrier® (GI Dynamics Inc, Lexington MA), have been designed to create an impermeable barrier between chyme exiting the stomach and the mucosa of the duodenum and proximal jejunum. The non-surgical and reversible nature of these devices represents an attractive therapeutic option for patients with obesity and T2DM by potentially improving glycaemic control and reducing their weight.

Methods and Analysis: In this multicentre, randomised, controlled, non-blinded trial, male and female patients aged 18–65 years with a BMI 30–50 kg/m^2 and inadequately controlled T2DM on oral anti-hyperglycaemic medications (HbA1C 58-97mmol/mol) will be randomised in a 1:1 ratio to receive either the EndoBarrier® device (n=80) for 12 months or conventional medical therapy, diet and exercise (n=80). The primary outcome measure will be a reduction in HbA1C by 20% at 12 months. Secondary outcome measures will include percentage weight loss, change in cardiovascular risk factors and medications, quality of life, cost, QALYs accrued and adverse events. Three additional sub-groups will investigate the mechanisms behind the effect of the EndoBarrier® device, looking at changes in: gut hormones, metabolites, bile acids, microbiome, food hedonics and preferences, taste, brain reward system responses to food, eating and addictive behaviours, body fat content, insulin sensitivity, and intestinal tissue gene expression.

Ethics and Dissemination: Research ethics approval was granted by Fulham Research Ethics Committee, London, (Reference 14/LO/0871) on 10th July 2014. All subjects will give informed written consent. Study findings will be disseminated through peer-reviewed journals, national and international conferences.

Strengths and Limitations of this study:

- This study will represent the largest randomised controlled trial of the EndoBarrier® device compared with conventional medical therapy, diet and exercise over a treatment period of 1 year and will also provide the longest follow-up data (1 year) of any trial to date.
- This study will provide: (1) unique data on the mechanism of action of the DJBS and the effect of foregut exclusion on an individual's metabolic profile, (2) a cost-effectiveness analysis, (3) quality of life assessment outcomes, and (4) extensive safety data.
- The unblinded design of this trial introduces the risk of bias.

Key words: Obesity, Type 2 diabetes mellitus, Endobarrier, Duodenal-jejunal bypass sleeve, Duodenal-jejunal bypass liner

Trial Registration: ISRCTN30845205, ClinicalTrials.gov Identifier NCT02459561

review only

Trial Sponsor:

Imperial College London
Faculty of Medicine Centre
Joint Research Office
Room 510B
5th Floor Lab Block
Charing Cross Hospital
Charing Cross Campus
Fulham Palace Road
W6 8RF
Tel: +44(0)203 311 0204
Fax: +44(0)203 311 0203

Contact person:
Mrs Ruth Nicholson
Tel: +44 (0)203 311 0212
Fax: +44(0)203 311 0203

Information concerning the study, patent applications, processes, scientific data or other pertinent information is confidential and remains the property of the Sponsor. Verbal or written discussion of results prior to study completion and full reporting should only be undertaken with written consent from the Sponsor.

Funding and Acknowledgements:

This project is funded by the Efficacy and Mechanism Evaluation (EME) Programme, an MRC and National Institute for Health Research (NIHR) partnership.

This paper presents independent research funded by the EME Programme and supported by the NIHR CRF and BRC at Imperial College Healthcare NHS Trust and University Hospital Southampton NHS Foundation Trust. The views expressed are those of the author(s) and not necessarily those of the EME Programme, the NHS, the NIHR or the Department of Health

This study is being executed with the support of GI Dynamics, Inc. and with kind support of Nutricia Advanced Medical Nutrition for providing oral nutritional supplements. Special thanks are given to Brunel University London and the Wolfson Centre for Translational Research, University of Surrey, for their support in sample analysis and to Professors Desmond Johnston, Jeremy Nicholson, and Elaine Holmes as co-applicants for the trial and for their valuable support in the set-up and design of this trial.

Competing interests statement:

Dr. Miras reports grants from Fractyl, personal fees from Novo Nordisk, and personal fees from Astra Zeneka outside the submitted work.

Dr. Goldstone reports funding supported by UK Medical Research Council and Wellcome Trust, outside of the submitted work.

Prof. Poulter's institution reports grants from HTA, during the conduct of the study.

Dr. le Roux reports grants from Science Foundation Ireland, grants from Health Research Board, during the conduct of the study; other from NovoNordisk, other from GI Dynamics, personal fees from Eli Lilly, grants and personal fees from Johnson and Johnson, personal fees from Sanofi Aventis, personal fees

For peer review only

from Astra Zeneca, personal fees from Janssen, personal fees from Bristol-Myers Squibb, personal fees from Boehringer-Ingelheim, outside the submitted work; .

Contributorship statement:

Mr Michael A Glaysher - Co-investigator at University Hospital Southampton. Corresponding author and primary author of manuscript.

Dr Aruchuna Mohanaruban - Co-investigator at Imperial College London. Contributed to writing manuscript and approved final version.

Dr Christina Gabriele Prechtl - Trial manager at Imperial College London, Imperial Clinical Trials Unit. Co-author of approved study protocol, contributed to writing manuscript and approved final version.

Dr. Anthony P. Goldstone - Co-investigator at Imperial College London and trial co-applicant. Co-author of approved study protocol, contributed to writing manuscript and approved final version.

Dr Alexander Dimitri Miras - Co-investigator at Imperial College London. Co-author of approved study protocol, contributed to writing manuscript and approved final version

Professor Joanne Lord - Director and Professorial Fellow in Health Economics. Co-author of approved study protocol, contributed to writing manuscript and approved final version

Dr Navpreet Chhina - Co-investigator at Imperial College London. Co-author of approved study protocol, provided critical appraisal of current manuscript and approved final version

Emanuela Falaschetti - Trial co-applicant. Trial statistician, contributed to writing manuscript and approved final version

Mr Nicholas Andrew Johnson - Trial statistician, contributed to writing manuscript and approved final version

Dr Werd Al-Najim - Co-investigator at Imperial College London. Co-author of approved study protocol, contributed to writing manuscript and approved final version

Dr Claire Smith - Operations and Business Development Manager, Imperial Clinical Trials Unit. Provided critical appraisal of current manuscript and approved final version

Dr Jia V Li - Contributed to approved study protocol, provided critical appraisal of manuscript and approved final version

Dr Mayank Patel - Co-investigator at University Hospital Southampton. Contributed to approved study protocol, provided critical appraisal of manuscript and approved final version

Mr Ahmed R Ahmed - Trial co-applicant. Contributed to trial set-up and design. Provided critical appraisal of manuscript and approved final version

Professor Michael Moore - Trial co-applicant. Contributed to trial set-up and design and was involved in the design of approved study protocol. Provided critical appraisal of manuscript and approved final version

Prof Neil Reginald Poulter - Trial co-applicant. Contributed to trial set-up and design. Provided critical appraisal of manuscript and approved final version

For peer review only

Professor Sir Stephen Bloom - Trial co-applicant. Contributed to trial set-up and design. Provided critical appraisal of manuscript and approved final version

Professor Ara Darzi - Trial co-applicant. Contributed to trial set-up and design. Provided critical appraisal of manuscript and approved final version

Prof Carel Le Roux - Trial co-applicant. Contributed to trial set-up and design. Provided critical appraisal of manuscript and approved final version

Mr James P Byrne - Principal investigator at University Hospital Southampton. Trial co-applicant, co-author for approved study protocol, provided critical appraisal of manuscript and approved final version

Prof Julian Teare - Chief investigator. Trial co-applicant, co-author for approved study protocol, provided critical appraisal of manuscript and approved final version

For peer review only

Protocol Version

Current version 4.0

Revision chronology:

- Minor amendment 3 29/09/16
  - Protocol V4.1 – number of randomised patients increased from 160 to 170
- Minor amendment 2 11/05/16
  - Extension of funding for 6 months
- Substantial amendment 10/08/15
  - Protocol V4.0 - Changes of inclusion (Section 4.2) and exclusion criteria (section 4.3)
  - Additional correction of minor transcription error in section 5.3
- Minor amendment 1 03/08/15
  - Protocol V3.1 - minor correction of inclusion criteria (section 4.2)
- Substantial amendment 16/03/15
  - Protocol V3.0 - Substantial amendment to protocol
  - Additional amendments to PIS, recruitment advert, business cards, news story.
- Substantial amendment 23/09/14
  - Protocol V2.0 - amendments to duration of liquid diet, questionnaires, blood sampling schedule, testing procedures and addition of biopsies.
  - Additional amendments to PIS, GP/consultant information sheets and letters, participant invitation letters, diet sheets, consent forms and questionnaires.
- Original submission 10/07/14
  - Protocol V1.0

Trial Steering Committee (TSC)

A Trial Steering committee (TSC) with an independent Chair will be appointed and will be responsible for overseeing the progress of the trial. A TSC Charter will be devised to list the roles and responsibilities of the TSC members. TSC will be convened biannually either in person or by teleconference. Two PPI representatives will sit on the Trial Steering Committee and will provide input from a patient perspective at trial meetings.

Trial Management Group (TMG)

The Trial Management Group will be set up by the Chief Investigator (CI). TMG will convene on a monthly basis and will discuss on the recruitment, and other practical aspects of the trial. The TMG will include the CI, Project Manager, ICTU representative and PI at the Southampton site as well as other site staff when appropriate. The day-to-day management of the trial will be co-ordinated through the Imperial Clinical Trials Unit via the Project Manager and the Chief Investigator.

Data Monitoring & Ethical Committee (DMEC)

An independent Data Monitoring and Ethical Committee (DMEC) will be set up to monitor progress, patient safety and any ethical issues involved in this trial. They will review trial progress, recruitment rates, event rates and safety data. A separate charter will be drawn up defining their exact remit and criteria for reporting to the trial steering committee. There will be 6-monthly meetings of the independent DMEC.

For peer review only

Background

Recent years have witnessed a global increase in obesity and obesity-related diseases. In 2014, it was estimated that 39% of the world population were overweight (clinically defined as a body mass index (BMI) of 25 – 30 kg/m^2) and 13% were obese (BMI ≥ 30 kg/m^2) and it has been projected that there will be an additional 11 million obese adults in the UK by 2030.[1] Being overweight or obese increases the risk of developing 'metabolic syndrome' and is the main modifiable risk factor for developing insulin resistance and type 2 diabetes mellitus (T2DM). Having a BMI of >25kg/m^2 increases the risk of developing T2DM by 5 times and 90% of adult patients with T2DM are obese or overweight.[2] The prevalence of T2DM has therefore also increased in recent years with an estimated 7.4% of the UK population currently affected and is projected to increase by a further 2.1% in the next 15 years.[3] Compared to the general population patients with T2DM are 87.6% more likely to be admitted to hospital for a myocardial infarction, 121.1% more likely to be admitted for heart failure, 59.1% for a stroke, and are 32% more likely to die prematurely.[4] This represents a significant socioeconomic burden for a largely preventable condition with combined healthcare costs for these conditions estimated to increase by up to 2 billion pounds each year in the UK.[5]

Adipose tissue is a highly active endocrine organ and acts to modulate metabolism by releasing pro-inflammatory cytokines (TNF-α, IL-6, MCP-1),[6] hormones (leptin and adiponectin), glycerol and, importantly, non-esterified fatty acids (NEFA).[7-11] In obesity, especially those with centrally placed adipose tissue, there is increased production of many of these mediators that leads to the development of insulin resistance and pancreatic beta cell dysfunction. T2DM occurs when an already insulin-resistant individual develops beta cell dysfunction and is therefore unable to produce the necessary amount of insulin that is required to maintain normoglycaemia and, as a result, hyperglycaemia predominates.

Dietary modification, exercise, and hypoglycaemic medication remain the mainstay of management for patients with T2DM. Unfortunately, these measures have generally sub-optimal and poorly sustained outcomes. Bariatric, or metabolic, surgery remains the most effective long-term means of treating these patients by producing usually profound and sustained weight loss and weight-loss independent improvements in insulin secretion and sensitivity, consequently ameliorating, or even eliminating, associated co-morbidities and reducing mortality. Roux-en-Y gastric bypass (RYGB) surgery can achieve approximately 23-35% weight loss and 72-90% of patients with T2DM undergoing RYGB are able to achieve sustained euglycaemia without oral hypoglycaemic agents.[12-19] There are several mechanisms by which these outcomes are achieved, namely: (1) gastric exclusion from food by producing a small gastric pouch, (2) exclusion of food from the duodenum and proximal jejunum, (3) early delivery of food to the terminal ileum, and (4) disrupted bile flow. Within the first few days and weeks following surgery, before weight loss has occurred, early improvements in glycaemic control occur through rapid modulation of hepatic insulin resistance (causing reduced hepatic glucose output). This is then followed by sustained long-term weight loss, via entero-neuro-hormonal mechanisms, with an associated reduction in peripheral insulin resistance.[17, 20]

Rubino et al. demonstrated in 2006 that the foregut plays a key role in the metabolic changes that occur following bariatric surgery.[21] They demonstrated that exclusion of the proximal small bowel, as occurs in RYGB and similar procedures such as biliopancreatic diversion (BPD) +/- duodenal switch (DS), results in improved glucose tolerance that occurs independently of effects from reductions in food intake and body weight, malabsorption, or nutrient delivery to the hindgut. These findings have further been substantiated in other studies.[21-24] The proposed mechanisms by which these changes occur include: decreased secretion of orexigenic hormones (ghrelin); increased secretion of glucose-dependent insulinotropic polypeptide (GIP) and cholecystokinin (CCK), increased anorexigenic and incretin hormone secretion (e.g. glucagon-like peptide 1 (GLP-1), peptide YY (PYY), oxyntomodulin), and increased circulating concentrations of plasma bile acids. Additionally, stimulation of vagal afferent nerves in the small bowel cause entero-neuro-endocrine modulation within the gut-liver-brain axis. The resulting net effects include: increased insulin secretion, decreased glucagon secretion, decreased hepatic glucose output, increased pancreatic beta cell mass (via increased proliferation and decreased apoptosis), increased insulin sensitivity, decreased hunger, early satiety and altered food preferences and hedonics and brain reward system responses away from high-energy foods.[20, 25-35]

For peer review only

Such observations have led to the development of novel, endoscopically-placed duodenal-jejunal bypass sleeves (DJBS) or liners. These create an impermeable barrier between chyme exiting the stomach and the intestinal mucosa of the duodenum and proximal jejunum, thus preventing absorption within the foregut. The non-surgical and reversible nature of these devices has sparked much interest in recent years due to the prospect of avoiding the associated surgical mortality and morbidity of bariatric procedures (RYGB: 1 year morbidity 14.9%, 30 day mortality 0.5%).[17] First described by Milone et al in animal models in 2006,[36] the effects of DJBS insertion on reducing weight and potentially improving glycaemic regulation, above that of control interventions, has been validated in five randomised controlled trials (RCTs) and numerous observational studies.[30, 37-49] In a recent systematic review and meta-analysis by Rohde et al.,[50] they concluded that subjects implanted with DJBS achieved an additional 12.6% weight loss compared to sham controls or dietary intervention alone, and a mean greater weight loss of 5.1kg. In the largest of the RCT (DJBS + diet n=38 vs. diet alone n=39) amongst the DJBS arm a significant reduction in HbA1C of -0.9% was found.[37] This finding however was not seen in the meta-analysis by Rohde et al. where the mean difference in HbA1C reduction of 0.8% was non-significant.[50] Finally, evidence exists for DJBS having positive effects on other metabolic parameters, including blood pressure and serum lipid profile.[37, 44, 45]

The EndoBarrier® DJBS (GI Dynamics Inc., Lexington, MA) is delivered endoscopically and comprises of a nitinol metal anchor, which is used to reversibly affix the device to the wall of the duodenal bulb, and an impermeable fluoropolymer sleeve that extends 60cm through the duodenum and into the jejunum (Figure 1). The implant is open at both ends to allow for passage of chyme from the stomach into the lower jejunum and prohibits nutrient absorption along its length by creating a barrier between the partially digested food and the absorptive surface of the small intestine. Whilst the chyme passes through the inside of the EndoBarrier® device, all bile and pancreatic secretions pass on the outside the liner and only mix with the food when they come into contact at the end of the sleeve.

Robust evidence for the clinical use of the DJBS is hence still lacking. The small number of published trials include small participant numbers with high degrees of inter-trial heterogeneity and the results are therefore not generalizable to routine clinical practice. Mechanistic data is also limited. There is therefore a call for more long-term, high quality trial data to validate the efficacy and mechanism of action of this device as a potential tool in the treatment of obesity and metabolic syndrome. In this paper, we describe the methodology for a government funded randomised controlled trial comparing DJBS against best practice medical therapy for the treatment of patients with obesity and T2DM with inadequate glycaemic control.

Methodology

Aims and Objectives

Primary Objective and Endpoint

The primary objective of this study is to evaluate the efficacy of DJBS compared with conventional medical therapy, diet and exercise on glycaemic control. As defined by the International Diabetes Federation (IDF) a substantial improvement in an individual's metabolic state occurs with an improvement in glycosylated haemoglobin (HbA1c) by 20%.[51] Our primary endpoint therefore is a:

i. Reduction in HbA1c by 20% after 12 months of treatment.

Secondary Objectives and Endpoints

The secondary objectives of this study are to evaluate the efficacy, acceptability and cost-effectiveness of DJBS compared against conventional medical therapy, diet and exercise. Secondary endpoints are:

i. HbA1C of < 6%, equivalent to 42 mmol/mol (this infers optimisation of the metabolic state as defined by the IDF)[51]
ii. Blood pressure < 135/85

For peer review only

iii. Weight loss > 15%
iv. Reduction in dose/number of medications
v. Cost of interventions and related health/social care
vi. Quality-adjusted life years (QALY) accrued (calculated from area under the EQ-5D-5L questionnaire curve)
vii. Incremental cost per QALY within the trial period and extrapolated through modelling.

Data will also be obtained to investigate the mechanism of action of the EndoBarrier® device via changes in:

i. Gut hormones
ii. Bile acids
iii. Microbiome
iv. Appetite
v. Food hedonics and preference
vi. Taste
vii. Eating behaviour
viii. Brain reward system responses to food evaluation and addictive behaviours using functional magnetic resonance imaging (MRI)
ix. Body fat content
x. Total body and tissue-specific insulin sensitivity
xi. Intestinal gene expression

Safety Objective

The safety of the EndoBarrier® DJBS will be evaluated during this trial and the type and frequency of adverse events shall be reported.

Research Approval

This study shall be conducted in full conformity with the 1964 Declaration of Helsinki and all subsequent revisions. Local research ethics approval was granted by Fulham Research Ethics Committee, London, (Reference 14/LO/0871) on 10th July 2014. All subjects will give informed written consent.

Study Design

This study is a randomised controlled trial of the EndoBarrier® DJBS compared with conventional medical therapy, diet and exercise for the management of subjects with both obesity and T2DM. Over a two year period (1 year of treatment and 1 year follow-up) the study will be performed over two investigational sites in the United Kingdom: Imperial College Healthcare NHS Trust in London and University Hospital Southampton NHS Foundation Trust. The overall schema for the trial is summarised in Figures 2a and 2b. To ensure that the study is adequately powered and allowing for drop-outs, n=80 patients will be randomised into each of the two treatment arms equally across the two sites (Table 1).

For peer review only

Table 1. Summary of treatment group

Treatment Group	Number of subjects	Treatment period 1	Follow-up period 2
EndoBarrier Device	80	12 months	12 months
Standard Medical Therapy	80	12 months	12 months
Total number of subjects	**160**		

In order to investigate the mechanism of the effect of the EndoBarrier® device, both treatment arms will be divided into three optional sub-groups, which will have the following additional assessments during the course of the trial:

- Sub-group 1: functional MRI of food reward and addictive behaviours, eating behaviour assessment and post-meal gut hormones.
- Sub-group 2: Euglycaemic, hyperinsulinaemic clamps (total body and tissue-specific insulin resistance).
- Sub-group 3: assessment of taste and food preference, eating behaviour assessment and post-meal gut hormones.

Table 2 summarises the visit schedule, the data to be collected across both study arms and supplementary data that will be collected from the three optional mechanistic sub-groups. In addition to routine follow-up visits, all patients will receive regular telephone counseling from a specialist dietitian to assess their wellbeing and motivation in the trial.

For peer review only

Table 2. Summary of study visit schedule

	Screening	Baseline		Treatment														Follow-up					
**Activities**	**V1**	**V2**	**V3**	**V4**	**T1**	**V5**	**V6**	**T2**	**V7**	**T3**	**V8**	**T4**	**V9**	**T5**	**V10**	**V11**	**T6**	**V12**	**T7**	**V13**	**T8**	**V14**	**V15**
Week/ Month/Day		- 4w ±7d	- 2w ±7d	- 0w ±3d	+ 5d± 3d	+ 10d ±3	+ 1m ±7d	+ 2m ±7d	+ 3m ±7d	+ 4.5m ±7d	+ 6m ±7d	+ 7.5m ±7d	+ 9m ±7d	+ 10.5m ±7d	+ 11.5m ±7d	+ 12m ±7d	+ 13.5m ±7d	+ 15m ±7d	+ 16.5m ±7d	+ 18m ±7d	+ 19.5m ±7d	+ 23m ±7d	+ 24m ±7d
Informed consent (5.1)	X																						
Inclusion & exclusion criteria (4.2, 4.3)	X																						
Demographics (5.1)	X																						
Medical history (including meds) (5.1)	X																						
Physical examination (5.5.4)	X																						
ECG (5.5.5)	X																						
Vital signs (5.5.7)	X	X	X	X		X	X		X		X		X		X	X		X		X		X	X
Body weight (5.5.8)	X	X	X	X		X	X		X		X		X		X	X		X		X		X	X
Height (5.5.8)	X																						
Waist circumference (5.5.9)	X	X	X	X		X	X		X		X		X		X	X		X		X		X	X
Routine blood tests (5.5.10)	X		X			X	X		X		X		X		X	X		X		X		X	X
Urine dipstick and female pregnancy test (5.5.6)	X																						
Changes in medical history/medication (5.5.4)		X	X	X		X	X		X		X		X		X	X		X		X		X	X
Randomisation (5.2)		X																					
Health Economic Questionnaires (5.5.12)			X			X	X		X		X				X							X	
Dietary counselling (5.5.3)		X		C																			
Dietitian follow up (5.5.3)							X		X				X			X		X		X			X
Urine albumin:creatinine ratio (5.5.11)			X			X					X				X							X	
Reporting of AEs (6)		X	X	X		X	X		X		X		X		X	X		X		X		X	X
DNA & RNA sampling (5.5.13)			X			X					X					X							X
Telephone counselling (5.5.3)					X			X		X		X		X			X		X		X		
Diabetologist Review (5.5.1 and 5.5.2)		X		C			X		X				X			C		X		X			X

For peer review only

Activities	Screening	Baseline		Treatment														Follow-up					
	V1	V2	V3	V4	T1	V5	V6	T2	V7	T3	V8	T4	V9	T5	V10	V11	T6	V12	T7	V13	T8	V14	V15
Week/ Month/Day		- 4w ±7d	- 2w ±7d	- 0w ±3d	+ 5d± 3d	+ 10d ±3	+ 1m ±7d	+ 2m ±7d	+ 3m ±7d	+ 4.5m ±7d	+ 6m ±7d	+ 7.5m ±7d	+ 9m ±7d	+ 10.5m ±7d	+ 11.5m ±7d	+ 12m ±7d	+ 13.5m ±7d	+ 15m ±7d	+ 16.5m ±7d	+ 18m ±7d	+ 19.5m ±7d	+ 23m ±7d	+ 24m ±7d
Metabolomics (5.5.14)			X			X					X				X							X	
Bioelectrical Impedance (5.5.15)			X			X					X				X							X	

EndoBarrier Group Only

Activities	V1	V2	V3	V4	T1	V5	V6	T2	V7	T3	V8	T4	V9	T5	V10	V11	T6	V12	T7	V13	T8	V14	V15
PPI and H. Pylori test (5.5.16)		X																					
Distribution of Proton Pump Inhibitors (5.5.2)		T																					
EndoBarrier Implant (5.5.2)				T																			
Preparation for EndoBarrier removal (5.5.2)													T										
EndoBarrier removal (5.5.2)																T							
Biopsies during Implant and Explant (5.5.2)				T												T							
Gastroenterologist appointment (5.5.2)		T					T		T				T			T		T		T*			**T**

Sub-groups

Activities	V1	V2	V3	V4	T1	V5	V6	T2	V7	T3	V8	T4	V9	T5	V10	V11	T6	V12	T7	V13	T8	V14	V15
Fixed/test meal and post-meal gut hormones and metabolites (Group 1 and 3) (5.6.4)			X			X					X				X								
Gut hormones and metabolites (Fasting only) (Group 1-3) (5.6.4)			X			X					X				X							X	
Food diaries (Groups 1-3) (5.6.2)			X			X					X				X							X	
Eating & Behaviour Questionnaires (Groups 1-3) (5.6.1)			X								X				X							X	
Appetite Visual Analogues Scales (Group 1-3) (5.6.3)			X			X					X				X							X	
Eating behaviour computerised tasks (Group 1 and 3) (5.6.5)			X								X				X							X	
Metal Check Form (Group 1) (5.1)	X																						
Handedness Questionnaire (Group 1) (5.1)	X																						

For peer review only

Activities	V1	V2	V3	V4	T1	V5	V6	T2	V7	T3	V8	T4	V9	T5	V10	V11	T6	V12	T7	V13	T8	V14	V15
Additional pregnancy tests			F								F												
	Screening	Baseline		Treatment														Follow-up					
Week/ Month/Day		- 4w ±7d	- 2w ±7d	- 0w ±3d	+ 5d± 3d	+ 10d ±3	+ 1m ±7d	+ 2m ±7d	+ 3m ±7d	+ 4.5m ±7d	+ 6m ±7d	+ 7.5m ±7d	+ 9m ±7d	+ 10.5m ±7d	+ 11.5m ±7d	+ 12m ±7d	+ 13.5m ±7d	+ 15m ±7d	+ 16.5m ±7d	+ 18m ±7d	+ 19.5m ±7d	+ 23m ±7d	+ 24m ±7d
DS-R disgust questionnaire (Group 1) (5.6.6)			X																				
Functional MRI (Group 1) (5.6.9)			X								X												
Insulin Clamps (Groups 2) (5.6.10)			X			X					X												
Cognitive assessment tasks (Group 1) (5.6.9)			X								X				X							X	
Food Preference / Taste Assessment(Group 3) (5.6.11)			X			X					X												
24hr Dietary Recall (Group 3) (5.6.11)			X			X					X				X							X	

X performed in all patients unless otherwise stated

F performed in Females only

C performed in Control arm (Standard medical therapy) only

T performed in Treatment arm (EndoBarrier) only

* optional (at request of the patient)

For peer review only

Study Population

Male and female patients, aged 18–65 years, with a BMI 30–50 kg/m^2 and confirmed diagnosis of T2DM for at least 1 year, who have inadequate glycaemic control and are on oral anti-hyperglycaemic medications. See Table 3 for complete inclusion and exclusion criteria.

Table 3. Study inclusion and exclusion criteria

Inclusion Criteria

1. Age 18–65 years (male or female)
2. Type 2 diabetes mellitus for at least one year
3. HbA1C 7.7–11.0% equivalent to 58 – 97 mmol/mol
4. On oral hypoglycaemic medications
5. BMI 30 – 50 kg/m^2

Exclusion Criteria

1. Language barrier, mental incapacity, unwillingness or inability to understand and be able to complete questionnaires.
2. Non-compliance with eligibility criteria.
3. Females of childbearing potential who are pregnant, breast-feeding or intend to become pregnant or are not using adequate or reliable contraceptive methods.
4. Evidence of absolute insulin deficiency as indicated by clinical assessment, a long duration of T2DM and a fasting plasma C-peptide of <333pmol/L.
5. Current use of insulin.
6. Previous diagnosis with type 1 DM or a history of ketoacidosis.
7. Requirement of NSAIDs (non-steroidal anti-inflammatory drugs) or prescription of anticoagulation therapy during the implant period.
8. Current iron deficiency and/or iron deficiency anaemia.
9. Symptomatic gallstones or kidney stones at the time of screening.
10. History of coagulopathy, upper gastro-intestinal bleeding conditions such as oesophageal or gastric varices, congenital or acquired intestinal telangiectasia.
11. Previous gastrointestinal surgery that could affect the ability to place the device or the function of the implant.
12. History or presence of active H. pylori (if subjects are randomised into the EndoBarrier® arm and have a history or presence of active H. pylori tested at study visit 2 they can receive appropriate treatment and then subsequently enrole into the study).
13. Family history of a known diagnosis or pre-existing symptoms of systemic lupus erythematosus, scleroderma or other autoimmune connective tissue disorder.
14. Severe liver impairment (i.e. AST, ALT or gGT >4 times upper limit of the reference range) or kidney impairment (i.e. estimated Glomerular Filtration Rate (GFR) < 45 ml/min/1.73m^2.
15. Severe depression, unstable emotional or psychological characteristics (including Beck Depression Inventory II score >28).
16. Poor dentition and inability to adequately chew food.
17. Planned holidays up to three months following the EndoBarrier Implant.

For peer review only

Study Recruitment

Participants will be identified from several areas across primary, secondary and tertiary healthcare and community settings:

i. Diabetes research registers (e.g. Diabetes Alliance for Research in England (DARE), REC 2002/7/118)
ii. Hospital or General Practice (GP) patient databases (Participant Identification Centres)
iii. Patients referred to diabetes and bariatric specialist clinics
iv. Other research studies within the Imperial College Healthcare NHS Trust and the Local Clinical Research Network (LCRN)
v. Study websites
vi. Local and national media – websites, radio, newspaper articles and adverts

vii. Posters
viii. Diabetes, Obesity and other support groups
ix. Social media websites

Potential patients who, after reading a summary Patient Information Sheet (PIS), would like to enter the trial will give their verbal consent for preliminary telephone screening to check basic inclusion and exclusion criteria. Written consent will then be taken from the patient to allow the study team to contact their GP for the purpose of obtaining additional information on the patient's medical history, current medical therapies and to identify any other clinical reasons as to why the patient should not participate. Patients who appear to meet eligibility criteria will be provided with a full trial PIS and then invited to a formal screening visit at one of the study centres. At this stage the patient will be fully informed of the nature of the study and given relevant information about the objectives of the research, benefits and possible adverse events, verbally and in writing. The patient will have the opportunity to ask questions about the trial and formal written consent will be taken for the patient to participate in the main study ± additional consent for one of the three optional mechanistic sub-groups. Once consent has been obtained then the subject's full eligibility will be checked against all inclusion and exclusion criteria (Table 3). Each patient will be informed of their eligibility for the trial once all results are available (usually within one week from obtaining consent).

Randomisation

Eligible patients will be randomised into one of the two trial arms using the InForm Integrated Trial Management system, a secure web-based data entry platform. This will be programmed with a randomisation schedule by an independent statistician and protect against bias in the randomisation process as group allocation will be concealed and automatic. The randomisation will be at a ratio of 1:1 and stratified by site and two BMI groups, 30–40 and 40–50 kg/m^2. Each patient will be informed of their randomisation allocation and will be assigned a unique study identification number. Only the subject number and initials will be recorded in the case report form (CRF). All other patient-identifiable data will be completely anonymised.

Trial Interventions

EndoBarrier® Gastrointestinal Liner

The EndoBarrier® Gastrointestinal liner device received CE Mark for 12 months implant duration on 11th December 2009 and is a single use, minimally invasive device, used to achieve weight loss and improve T2DM status in subjects who are obese (Figure 1).

At visit 2 (- 4 weeks), participants who have been randomised to receive the EndoBarrier® device will be tested for the presence of Helicobacter pylori, either by faecal antigen or urea breath testing. Those patients testing positive will be offered 1 week of triple-eradication therapy, as per guidance published within the British National Formulary, and will then be retested after a further 4 weeks to confirm complete eradication before continuing with implantation of the EndoBarrier® device. Subsequently, all patients will be prescribed a proton pump inhibtor (PPI) (Omeprazole 40mg twice daily) and instructed to commence this three days prior to the implant procedure. They will continue this for the duration of the implant period (12 months) and for a further two weeks following device removal.

At visit 4 (0 weeks), after an eight-hour fast, subjects will have the EndoBarrier® device implanted under a general anaesthetic. The implant is delivered endoscopically on a custom catheter and the anchor is sited in the duodenal bulb using a custom delivery system under fluoroscopic x-ray guidance (mean fluoroscopic x-ray time for insertion is 7 minutes, range 1-20 minutes). The 60cm sleeve is unfurled and then the final positioning plus patency is confirmed by assessing for the free flow of radio-opaque contrast through the device. Videos and photos of the fluoroscopy images are recorded to help the investigators make treatment decisions. During implantation eight gastric and small bowel biopsies will be taken using standard biopsy forceps. Four biopsies will be used for routine histology and four biopsies will be used for RNA extraction to perform genome-wide expression analysis. Participants will be discharged from hospital the same day with an implant information card, which describes the implant, identifies who to call in

For peer review only

the case of an emergency, and what symptoms to look for following the implant. Subjects will have their dose of sulphonylurea medication reduced by 50% at the time of EndoBarrier® implant to avoid potential hypoglycaemic episodes.

The device will be removed at visit 11 (after 12 months) under sedation or general anaesthetic. The gastroscope, which is fitted with a foreign body retrieval hood, is used to locate the implant and a custom grasper is passed through the working channel of the gastroscope to grab a polypropylene tether located on the proximal portion of the anchor. Pulling on this tether will collapse the proximal end of the anchor, which can then be pulled into the foreign body hood and removed by withdrawing the gastroscope through the subject's mouth. During this removal, eight further biopsies will be taken for histology and RNA extraction. Following removal of the EndoBarrier® device, patients will be followed up for a further 12 months.

Diabetes Review

Participants in both arms of the trial will have their T2DM managed in accordance with the guidelines of the American Diabetes Association (ADA).[52, 53] These guidelines have been chosen as they would adhere to the current best worldwide practice that would still be relevant when the results are published following study completion. Both treatment groups will have a review of their T2DM by a suitably trained physician at visits 2, 6, 7, 9, 12, 13 and 15. Additionally, the standard care arm of the trial will have an additional review at visits 4 and 11 in place of the EndoBarrier® implant and removal. Adjustments to a patient's oral anti-hyperglycaemic medication and escalation of therapy is at the investigators discretion and will comply with general recommendations laid out by the ADA.[53]

Dietary Counselling and Physical Activity

At visit 2, all patients historical and current eating behaviours will be assessed by a qualified dietitian using the following information: anthropometry; biochemistry; co-morbidities; activity levels; eating habits including previous diets; lifestyle including smoking, drug and alcohol misuse; weight history; psychiatric history; family history of obesity, diabetes, mental illness or eating disorders; available support network; work status; readiness and motivation for change. Patients will then receive dietary and physical activity counselling in accordance with local standards with the intention of providing each subject with lifestyle/behavioural modification information and good eating practices. In addition, subjects in the EndoBarrier® arm will receive written information on how their diet will change after implantation of the device and they will receive specialist guidance for eating with their EndoBarrier®.

All patients will be reviewed by a specialist dietitian at visits 2, 6, 7, 9, 12, 13 and 15. In addition, participants in the standard care arm of the trial will have an additional review at visits 4 and 11 in place of the EndoBarrier® implant and removal. During the course of the trial, participants will be recommended to consume 600 kcal less every day, depending on their age, gender, activity levels and body weight. Guidelines for daily amounts are between 1200 and 1500 kcal for women and between 1500 and 1800 kcal for men. In accordance with standard dietary practice, subjects will be advised: to eat regularly every day (5 times per day); to control their portion sizes and intake of carbohydrates/starchy foods; to increase their intake of low glycaemic index (GI) and high protein foods, as well as vegetables; and to reduce their intake of foods high in fat and sugar, and alcohol. Participants will be advised to include more physical activity in their daily routine and encouraged to do more activity in their leisure time. Their goal will be to include 150 minutes a week of moderate intensity, and 75 minutes a week of vigorous intensity aerobic activity and muscle strengthening activities, on more than 2 days a week. Changes in physical activity level will be monitored using the International Physical Activity Questionnaire (IPAQ).[54]

Liquid Diet

To avoid disruption of the device in the immediate period following implantation, patients will follow a liquid diet for the 7 days before and 13 days (± 3 days) after the intervention visit (visit 4). The liquid diet will be guided by the

For peer review only

specialist dietitian and will comprise of 125ml Fortisip Compact drinks (Nutricia, UK): 5 per day for males, 4 per day for females, containing per 100 mL: 240 kcal, 9.6g protein (16% total energy), 29.7 g carbohydrate (49%), 15g sugars, 9.3g fat (35%). Patients will also be allowed to consume sugar-free squashes, smooth/clear soup (1 medium bowl per day), tea or coffee without sugar, or unsweetened puree. To standardise both therapy groups, all patients across both arms will follow the liquid diet for this duration and period of the study.

Assessment of Objectives

Assessment of Primary Objective

Each study participant will have their IFCC (International Federation of Clinical Chemistry) HbA1C measured at screening and then subsequently at visits 5, 7, 8, 9, 10, 12, 13 and 15. Samples will be processed at the laboratory local to each study centre using standard methods. Results will be recorded on the InForm system.

Assessment of Secondary Objectives

Individuals in both study arms will be invited for regular medical check-ups (Figure 2), which will include routine anthropometric measurements (height, weight, waist circumference, pulse and blood pressure) and blood tests (Table 4). Any changes to the participants' health or medications will be carefully documented on the CRF and all adverse events will be reported in detail in line with standard principles of GCP.

Table 4. Summary of blood tests at each study visit

Blood test	V1	V3	V5	V6	V7	V8	V9	V10	V11	V12	V13	V14	V15
Haematology (Full blood count)	x	x	x	x	x	x	x	x	x	x	x	x	x
Routine Biochemistry (including Urea and Electrolytes)	x	x	x	x	x	x	x	x	x	x	x	x	x
Liver function tests	x	x	x	x	x	x	x	x	x	x	x	x	x
Fasting glucose	x	x	x	x	x	x	x	x	x	x	x	x	x
Creatinine	x	x	x	x	x	x	x	x	x	x	x	x	x
HbA1C	x		x		x	x	x	x		x	x		x
Fasting lipids (cholesterol, HDL, LDL, triglycerides)	x	x	x	x	x	x	x	x		x	x	x	x
C-peptide	x												
Insulin (Fasting)	x	x	x			x		x				x	
Vitamin D	x					x		x					
Iron studies	x					x		x					
Vitamin B12	x					x		x					
Serum Folate	x					x		x					
Free Thyroxine	x							x					x

For peer review only

TSH	x							x					x
Cortisol (Sub-group 1 only)		x				x							
Oestradiol (Sub-group 1 only)		x				x							
Progesterone (Sub-group 1 only)		x				x							
LH (Sub-group 1 only)		x				x							
FSH (Sub-group 1 only)		x				x							

Patients in both treatment arms will be asked to complete health economics questionnaires at visits 3, 5, 6, 7, 8, 10 and 14. These comprise of the EQ-5D-5L questionnaire to assess health-related quality of life and a bespoke questionnaire designed to collect information about patients' use of health and social care resources (for costing purposes).[55] The Resource Use questionnaire will be adapted from existing instruments and will include:[56, 57]

i. Medications for diabetes, weight loss, blood pressure, lipid control and cardiovascular disease
ii. Primary care consultations (with GP, nurse or other healthcare professional)
iii. Hospital outpatient clinic visits (by specialty)
iv. A&E attendances (admitted/not-admitted)
v. Inpatient stays and procedures
vi. Investigations
vii. Use of any other NHS-related community health and social services (e.g. chiropody).

Costs for private health and social care, out-of-pocket expenditure by patients, and 'indirect costs' per patient time will not be included.

In addition, data will be collected in the CRF from hospital information systems and case notes to cost the EndoBarrier® intervention and the diet/exercise intervention. Information to be collected will include:

i. Routine assessments required before implantation and removal of the EndoBarrier®
ii. Disposables and staff time for the insertion and removal procedures, including day case and overnight stay if required
iii. Treatment of any adverse events related to the procedures (additional inpatient stays, clinic visits and re-admissions)
iv. Dietitian time to deliver the diet and physical activity counselling and for telephone follow-up
v. Routine hospital follow-up and diabetes care
vi. Hospital treatment for cardiovascular events or other complications of diabetes

Mechanistic Study Groups

In addition to the routine data collected above, at visits 3, 5, 8, 10, and 14, mechanistic data will be gathered from all patients across both study arms:

i. Body fat mass (kg and % of body weight) measured by bio-electrical impedance analysis
ii. Collection of stool, urine and plasma for assessment of metabonomics using nuclear magnetic resonance (NMR) spectroscopy and mass spectrometry (MS), and microbiome analysis
iii. Measurement of insulin, gut hormones (ghrelin, GLP-1, PYY), bile acids, leptin and other adipocytokines, and markers of insulin resistance and inflammation from venous blood samples (fasting for all visits and then

For peer review only

following a meal in sub-groups 1 and 3 at visits 3, 5, 8 and 10)
iv. DNA and RNA from venous blood samples for examination of genetic variants that may predict weight loss, cause or contribute to obesity
v. Urinary albumin:creatinine ratio
vi. Comprehensive 3-day food diary

Sub-group 1: Functional MRI

Subjects in each study arm, at the London site only, will have fMRI scans to examine brain function related to food reward and addictive behaviours contributing to overeating at baseline (visit 3) and at 6 months (visit 8) after intervention. These will be supplemented by and correlated with psychological questionnaires, computerised tasks and test meals at these and other visits. The study visits will last up to 6-8 hours (scanning visits 3 and 8). Subjects will have structural and functional MRI brain scans lasting up to 90 minutes using a 3.0 Tesla Siemens Verio MR scanner after an overnight fast. Whilst in the scanner, subjects view a mirror reflecting a computer screen and can respond to instructions using a keypad held in their hand.

The following anatomical brain scans will be collected at both visits:

i. Anatomical T1 and T2-weighted MR scans to provide structural neuroimaging data and allow image registration to standard space.
ii. Diffusion tensor imaging to examine white matter tract integrity.

The following resting state functional MRI scans will be collected at both visits:

i. Arterial spin labelling to measure resting regional cerebral blood flow.[58, 59]
ii. Resting state blood oxygen level-dependent (BOLD) functional MRI to measure resting state functional connectivity.[60]

The following task-related functional MRI scans will be collected at both visits:

i. Food picture evaluation task: to assess reward system activation when subjects view a variety of different pictures (high-energy and low-energy foods, household objects, blurred pictures as a baseline) and simultaneously rate how 'appealing' the pictures are using the keypad as a measure of anticipatory food reward or food due reactivity.[32, 34, 61-63]
ii. Monetary incentive delay (MID) task: a game in which subjects need to press a button during a specific time window when given a cue on the computer screen in order to win or prevent the loss of hypothetical monetary prizes to assess anticipatory non-food reward responsivity.[64-66]
iii. Go-NoGo task: to assess motor response inhibitory control as a measure of compulsivity.[67-69] The task contrasts brain activation during responses to infrequent no-go signals (e.g. 'do not press' button when viewing one symbol) compared to an implicit go baseline (e.g. 'do press' button when viewing a different symbol).[66]
iv. Negative emotional reactivity task: to assess brain response during viewing of unpleasant, negatively valent pictures compared to neutral control pictures.[66]

Study visits without scanning at visits 5, 10 and 14 will last 3-6 hours. At visits 3, 8, 10 and/or 14, subjects in sub-group 1 will also complete several questionnaires and perform several computer-based tasks to assess eating and addictive behaviours and cognition, including:

i. WTAR word reading list: to document baseline intellectual status (visit 3 only)
ii. Kirby delay discounting task: to assess temporal impulsivity to a hypothetical non-food monetary rewards (visits 3, 8, 10 and 14).[70]
iii. Leeds Food Preference Questionnaire: to assess bias of food preference to foods high in fat and sugar and explicit and implicit liking of foods high/low in fat/sugar (visits 3, 8, 10 and 14).[71]
iv. Progressive ratio task: to measure breakpoint of effort that subjects are willing to spend by having to press a computer mouse an increasing number of times to receive a chocolate M&M™ sweet, to assess appetitive food reward and motivation (visits 3 and 8).[33, 34]

An *ad libitum* test meal will be performed at visits 3 and 8, in which subjects first taste and rate the palatability,

For peer review only

intensity and acceptability of different foods high or low in fat and sugar, and then eat as much of whichever foods they want, to assess food taste, preference and choice, total energy intake and macronutrient composition. Subjects will also complete visual analogue ratings of appetite, anxiety, stress and sleepiness, and measurement of fasting and post-meal hormones and metabolites over the study visit.[32, 63]

At visit 5, 10 and 14 subjects will consume a fixed mixed meal tolerance test with measurement of fasting and post-prandial hormones and metabolites.

Sub-group 2: Euglycaemic, hyperinsulinaemic clamp

On visit 3, 5, and 8, patients in each study arm, at the Southampton site only, will undergo a euglycaemic hyperinsulinaemic clamp with stable isotope infusion to determine overall insulin and compartment-specific insulin sensitivity (liver, muscle and adipose depot). Patients will be instructed to consume a standardised meal or meal replacement the evening prior to their study visit. A venous catheter will be inserted into a vein of each arm on the study morning. The first cannula will be used for infusions and the other for blood sampling. If blood glucose levels are higher than 6 mmol/L on arrival then a variable rate insulin infusion will be started to attain a stable glucose level (4.0 -6.0 mmol/L) prior to commencement of the hyperinsulinaemic euglycaemic clamp.

A primed continuous infusion of 6, 6-$^{2}H_2$-glucose, a stable isotope tracer, will be started and maintained for 7 hours. Two hours later a two-stage hyperinsulinaemic-euglycaemic clamp procedure will be started and continued for 5 hours. During stage 1 of the clamp procedure, in which hepatic insulin resistance is assessed, insulin will be infused at a low dose (0.3-0.5 mU/kg/min) for 2 hours. During stage 2 of the clamp procedure, in which peripheral insulin resistance is assessed, insulin will be increased to a higher dose (1.5 mU/kg/min) for 2 hours. Euglycaemia will be maintained by infusing 20% dextrose at a variable rate. Blood samples will be taken every 5 minutes to measure blood glucose concentration and the dextrose infusion will be adjusted accordingly. The exogenous glucose infusion will be enriched with 6, 6-$^{2}H_2$-glucose to prevent a fall in plasma tracer enrichment and underestimation of endogenous glucose production rate. Blood samples will be obtained before the start of the tracer infusions, every 10 min during the final 30 min of the basal period and stages 1 and 2 of the clamp procedure and every 30 minutes between these periods to determine glucose enrichment and concentration, free fatty acid, insulin, C-peptide, glucagon, gut hormones, and metabolite concentrations. At the same time points participants will be asked to complete appetite visual analogue scales.

The isotopic enrichment of plasma glucose will be determined by gas chromatography mass spectrometry (GCMS) at the Wolfson Centre for Translational Research, Postgraduate Medical School, University of Surrey, United Kingdom.

Sub-group 3: Taste and Food Preference Assessment

On visit 3, 5, 8, 10 and 14, patients in each study arm, at both the London and Southampton sites, will attend the research facility after an overnight fast. The total duration of these visits will be up to 7 hours (visits 3, 5, 8, 10) and 5 hours (visit 14). On the morning of those visits patients will perform two behavioural computerized tasks: the Kirby Delay Discounting Task to assess temporal impulsivity to non-food monetary reward (visits 3, 8, 10 and 14);[70] and the Leeds Food Preference Questionnaire to assess bias of food preference to foods high in fat and sugar and explicit and implicit liking of foods high/low in fat/sugar (visits 3, 8, 10 and 14).[71]

Sweet taste detection testing will be performed at visits 3, 5, 8 by following the method of constant stimuli in which seven ascending sucrose concentrations in solution will be used to determine sweet detection thresholds.[72] At the same visits consummatory taste reward will be assessed in which five ascending sucrose solutions will be used to test responses in intensity ratings and hedonic reward. To assess the appetitive behaviour towards a sweet and fatty food reward, a Progressive Ratio Task (PRT) will be performed 2 hours after the consumption of a fixed meal.[33]

Total caloric intake and macronutrient composition will be assessed using three-day food diaries and a 24-hour recall that is carried out by a trained dietitian/nutritionist on all visits. Patients will also complete the EPIC Food Frequency questionnaire at visit 3, 8, and 10. Finally, a fixed mixed meal tolerance test with measurement of post-meal hormones and metabolites will be performed.

For peer review only

Additional sub-group specific procedures and measurements

Across all three sub-groups only, the following additional data will also be collected during the mechanistic study visits:

i. Trait, state and symptom questionnaires: to evaluate aspects of eating behaviour (e.g. dietary restraint, emotional eating, disinhibition, hunger, external eating), reward sensitivity, mood, impulsivity, aversive symptoms, symptoms of dumping syndrome, including Dutch Eating Behaviour Questionnaire, Three Factor Eating Questionnaire, Yale Food Addiction Scale, Binge Eating Scale, Beck Depression Inventory II, Hospital Anxiety and Depression Scale (HADS), Barratt Impulsiveness Scale (BIS), UPPS-P Impulsive Behavior Scale.

ii. Visual analogue scale (VAS) ratings: to assess subjective feelings of hunger, nausea, fullness, sleepiness, stress and anxiety when fasted and during meal tests.

Power Calculation

Conservatively, it was estimated that 15% of patients in the control arm will achieve the target but we believe this to be an overestimate. The Steno study is the best quality randomised study (n=80 patients in each arm) into the effect of best medical therapy published to date and demonstrated over an average 7.8 years significant improvements in HbA1c amongst those having intensive medical therapy from 8.4 ± 1.6 to 7.7 ± 1.2, but no change in HbA1c amongst those continuing with standard medical therapy.[73] This study defines the very best that could realistically be achieved in the control arm, but expect there to be very little if any change in this group. The reporting of HbA1c as an outcome measure was not in accordance with the newly defined IDF criteria, but considering the small average reduction achieved in the Steno study, it will be assumed that a target of 15% of patients reaching the endpoint is a conservative estimate. Company data on the small number of patients who have reached a year with the device in place suggest that 40% will achieve this target.

According to our own experience with the device in a commercially sponsored study, up to 30% of patients in the treatment group may have the device removed early. Nevertheless other commercially sponsored (unpublished) studies of this device have achieved lower explant rates (J Tetreault – GI Dynamics). To allow for up to 30% early removal we have therefore diluted the treatment effect from 40% vs. 15% to 35% vs. 15% achieving the target of 20% reduction in HbA1c for treatment arm vs. standard arm. With these assumptions, n=73 patients per group will give 80% power with a two-sided alpha 0.05 to detect a significant effect. Adding 10% loss of follow-up increases the sample size to n=80 per group.

The dilution was calculated starting from the assumption that 40% of patients with the device will reach the target (this estimate is based on company data based on diabetic patients in the same range of BMI as in the present proposal). If 30% of patients in the treatment group need to remove the device early but remain available for follow-up, in the worst case scenario, the proportion reaching the target is the same as in the control group, bringing the estimate for the treatment group to 32.5%. However most of them will keep the device for some time, having some benefit, so it is plausible to assume that the estimate is higher than 32.5%. Dividing the main effect 15% vs. 40% in three parts we assume that in the 30% of patients with removal, for 1/3 the same effect will be achieved as in the control group (15% reaching the target), for 1/3 it will be increased (23% reach the target) and for 1/3 more increased (31% reach the target). Overall, this would give an estimate of 35% for the treatment group.

Statistical Analysis

Patient characteristics will be summarized. Summaries of continuous variables will be presented as means and standard deviations if normally distributed, and as medians and inter-quartile ranges for skewed data, whilst categorical variables will be presented as frequencies and percentages. The difference between the two study groups in the proportion of patients achieving substantial improvement in the metabolic syndrome both at 12 and 18 months will be analysed using logistic regression adjusting for the stratification variables (BMI groups and sites). Analysis of secondary outcomes will be conducted using standard statistical procedures applicable to categorical or continuous data as appropriate. For missing values we will explore the pattern and the extent of missingness and we

For peer review only

will carry out an appropriate form of multiple imputation if required. The analysis will be performed according to the intention to treat principle. All statistical tests will be two-tailed with a 5% significance level.

Metabonomics and Microbiome Analyses

Metabonomic datasets will be analysed using principal component analysis (PCA) and orthogonal partial least-squares analysis (O-PLS). The metabolic and microbial data will also be analysed in relation to response measurements such as BMI, gut hormone levels etc. using O-PLS regression analysis and Bayesian approaches. A range of statistical methods will be optimised and applied to the data to identify weight loss and T2DM-associated microbiota and metabolites.

Health Economics

The economic health analysis will be conducted following the NICE Reference Case, which includes the use of QALYs as the measure of health outcome, and adoption of an NHS and Personal Social Services perspective for costs (Guide to the methods of technology appraisal 2013, http://publications.nice.org.uk/pmg9).

Within trial analysis

EQ-5D-5L health states will be scored using the English value set to give a utility values at each time point (-2 weeks, 10 days, 1, 3, 6, 11.5, and 23 months).[74] QALYs will be estimated for each patient using an area-under-the curve approach.

The cost of the EndoBarrier® intervention, the lifestyle intervention, and other related health and social care will be estimated from resource use data. Unit costs for the included services will be obtained from standard national sources (BNF or Drug Tariff for drug prices, Department of Health Reference Costs for investigations, procedures and outpatient visits, PSSRU estimates for other primary and community health and social services). Total costs will be estimated for each patient over the 24-month trial period.

Patient-level cost and QALY estimates will be combined to estimate an Incremental Cost Effectiveness Ratio (ICER) for the EndoBarrier® device compared with standard medical therapy over the 24 month trial period. The analysis will combine multiple imputation to account for missing EQ-5D and resource use data with bootstrap regression to estimate mean cost and QALYs for the two patient groups. Missing data is often a particular problem for economic analysis, even in studies with good follow-up of primary endpoints, as area under the curve approach requires data from multiple time points. A bootstrap regression approach will be used to account for non-normal distributions of cost data, to adjust for baseline differences in utility or other patient characteristics, and to allow for correlations between costs and QALYs.

Cost-effectiveness modelling

A decision analytic model will be developed to estimate clinical outcomes, QALYs, and costs beyond 24 months. The time horizon for the modelling will be for the remainder of the patient's predicted lifetimes, as recommended in modelling guidelines.[75, 76] Before commencing this modelling exercise, a review of published economic decision models for weight loss interventions for people with T2DM will be conducted in order to identify possible model structures and sources of input parameters. The conceptual design of the model will be discussed and agreed amongst the research team before programming commences. It is anticipated that the model will take the form of an individual patient simulation, using either a discrete time or discrete event approach to simulate the onset of diabetes/obesity related complications, and hence QALYs and costs under alternative treatment strategies.

Data from the trial will be used to provide estimates of the effect of the EndoBarrier® compared with conventional management. In addition, published systematic reviews and meta-analyses of bariatric surgery for similar a patient population will be reviewed. If recent evidence of sufficient relevance and quality is available, we will extend our model to include indirect comparisons with these other interventions. Other model parameters will be sourced from targeted literature reviews and routine data sources. The choice of software for the model will be made after specification of the conceptual design. Before use, the model will be validated by an experienced health economist not involved in the development of the model. This will be done using a checklist developed by the Brunel Health

For peer review only

Economics Research Group, which includes a range of suggestions for checking that a model is free from errors (verification) and that it is consistent with internal and external data (validation).

Probabilistic sensitivity analysis (PSA) will be used to estimate the impact of uncertainty over model parameters, and value of information (VOI) analysis to estimate the value of conducting further research. In addition, deterministic sensitivity analysis will be used to examine the impact of uncertainties over the model structure.

Gut hormones, metabolites and bile acids

These will be measured in the fasted and/or postprandial state for each patient and compared within and between the groups using parametric/non-parametric repeated measures statistical testing.

Food hedonics and brain reward responses

Brain activation during fMRI paradigms and outcomes from behavioural measures of eating and addictive behaviours and questionnaires, will be compared between groups using a 2x2 ANOVA design including group (control vs. EndoBarrier®) as a between subject factor, time (baseline vs. follow-up visit) as a within subject factor, and group x time interaction to identify differential effects between groups. For fMRI studies, analysis will use region of interest (e.g. for food picture evaluation task: orbitofrontal cortex (OFC), amygdala, caudate, nucleus accumbens, anterior insula) and whole brain analyses to compare groups using statistical thresholds of voxel-wise correction false discovery rate (FDR) $P<0.05$ or cluster-wise family wise error (FWE) correction $P<0.05$. Correlations of BOLD signal will be made with other behavioural variables by linear regression analysis to examine the relevance of changes in brain activation.

Food Preference and Sweet Taste

Dietary energy intake, macronutrient composition, sweet taste detection thresholds, and visual analogue taste ratings will be quantified for each patient and compared within and between the groups at different time points using parametric/non-parametric repeated measures statistical testing. Regressions will be performed with clinical outcomes (e.g. weight loss, HbA1c) to identify predictive markers and generate mechanistic hypotheses.

Hyperinsulinaemic Euglycaemic Clamps

Overall and tissue specific insulin sensitivity will be quantified for each patient and compared within and between the groups at 3 time points using parametric/non-parametric repeated measures statistical testing.

In addition, linear regression will be performed to correlate mechanistic variables collected from each of the sub-groups 1-3 at baseline or during the intervention with clinical outcomes at 1 year e.g. weight loss and decreases in HbA1c, to generate predictive markers and generate mechanistic hypotheses.

Discussion

Experience of 3717 EndoBarrier® devices distributed worldwide has demonstrated a favourable risk-to-benefit ratio (GI Dynamics, February 2017) and their minimally invasive and reversible nature represents a very attractive treatment modality for patients with obesity and T2DM. Evidence already exists in the literature in support of the efficacy of DJBS by reducing weight and potentially improving glycaemic control.[37, 38, 40, 42, 44-46, 50] Nonetheless, it is reported that up to 100% of patients will experience a non-serious adverse event (predominantly abdominal discomfort and nausea immediately following implantation)[50] and 7.4% will suffer a serious adverse event (SAE) (GI Dynamics safety reporting 2008 to March 2017). The exact nature of these events are summarised in Table 5.

For peer review only

Table 5. Serious Adverse Events from the EndoBarrier® device

	2008	2009	2010	2011	2012	2013	2014	2015	2016	2017 (Jan-Mar)	TOTAL
Distributed (Denominator)	25	143	157	275	391	812	987	482	383	43	
Hepatic Abscess	0	0	0	0	1	8	12	9	4	2	36
Hepatic Abscess Rate											1.0%
Hepatic abscess with explant ≤ 12 months	0	0	0	0	1	5	9	7	1	2	25
Hepatic Abscess Rate											0.7%
Intolerance	0	5	4	12	5	10	11	4	18	0	69
Intolerance Rate											1.9%
Liner Obstruction	0	4	0	5	3	1	10	2	0	0	25
Liner Obstruction Rate											0.7%
GI Bleed	0	1	3	6	5	9	20	8	4	1	57
GI Bleed Rate											1.5%
Migration/ Movement	0	10	6	4	1	6	11	13	0	1	52
Migration/Movement Rate											1.4%
Pancreatitis	0	0	0	0	4	1	7	0	0	0	12
Pancreatitis Rate											0.3%
Perforation	0	0	1	0	0	5	2	2	1	0	11
Perforation Rate											0.3%
Surgical Removal	0	0	0	1	1	0	8	1	1	0	12
Surgical Removal Rate											0.3%
Total Incidences	0	20	14	28	20	40	81	39	28	4	274
Total Cumulative Rate											7.4%

GI dynamics safety reporting 2008 to March 20

Notably, the pivotal U.S ENDO trial (EndoBarrier® vs. Sham procedure) was terminated in July 2015 after only 325 subjects were randomised (n=216 EndoBarrier subjects) due to a higher than expected hepatic abscess (HA) rate of 3.5% (compared to a global incidence of 0.73%). This high incidence of HA is not the experience within Europe with 1.2% being reported in 1901 distributed devices (UK hepatic abscess rate in 523 cases is 1.34%). There have also been no deaths attributed to the EndoBarrier® and all patients experiencing an SAE have recovered without long-term sequelae.

Research to date therefore validates the EndoBarrier® DJBS as a potential treatment option for patients who are obese with or without T2DM. These studies however have been limited by their low participant numbers, short follow-up duration and wide inter-trial heterogeneity. Thus, there is a call for more robust clinical trial data to investigate its efficacy, safety and acceptability, and to establish where its use may fall within the treatment algorithm of such patients. This study will represent the largest randomised controlled trial of the EndoBarrier® device compared with conventional medical therapy, diet and exercise over a treatment period of 1 year and will also provide the longest follow-up data (1 year) of any trial to date. Additionally, this study will provide: (1) unique data on the mechanism of action of the DJBS and the effect of foregut exclusion on an individual's metabolic profile, (2) a cost-effectiveness analysis, (3) quality of life assessment outcomes, and (4) extensive safety data.

As this study is an open trial, in which the participants, clinicians and hospital staff will not be blinded to their treatment allocation, it is at risk of bias, particularly performance or observer bias. A control group undergoing a sham endoscopy would significantly reduce this bias but would expose a large number of patients to the risks of an unnecessary endoscopic procedure and general anaesthetic. Therefore, to reduce the effect of bias: (1) participants will undergo a concealed computer-generated randomisation process by an independent statistician, (2) multiple assessors across both study sites will follow structured assessment protocols and utilise validated measurement tools in order to minimise subjectivity from the data collection, (3) data collection will be monitored regularly to ensure adherence to the protocol and to perform source data verification, and (4), where possible, outcomes and results will be reported by an independent person who is unaware of the treatment allocation of the participant (e.g. the primary outcome measure of HbA1c and all other haematological or biochemical samples will be measured and reported by an independent laboratory technician at each hospital). Attrition bias will be minimised by performing regular scheduled follow-up visits across both treatment groups and regular telephone follow-ups will be performed in order to assess the patient's wellbeing and motivation on the trial. Patients selected for this trial will be a very motivated subset of the population of interest. The effects of this sampling bias will be minimized through effective randomization but will reduce the generalisability of any significant treatment effect identified.

To conclude, we hypothesize that exclusion of the foregut by means of an EndoBarrier® device will improve glycaemic control, above that of conventional medical therapy, diet and exercise via: (i) decreased hepatic insulin resistance and increased insulin production, that occurs independent of weight loss and caloric restriction, and (ii) through reduction in total body and tissue-specific insulin resistance as a result of consequent weight loss. We also hypothesize that this device will produce weight loss, above that of control patients, by reducing hunger, increasing satiety (therefore reducing food intake) and changing food preferences and hedonics away from high-energy sweet and fatty foods. If the EndoBarrier® is effective at achieving long-lasting weight loss and glycaemic control, there is an obvious potential for health benefit and savings on future health and social care; through the avoidance of T2DM and related complications.

Trial Status

The trial opened for recruitment at Imperial College Healthcare NHS Trust in London on 18th November 2014 and then in University Hospital Southampton NHS Foundation Trust on 5th June 2015. Recruitment was completed across both sites on 18th October 2016 and all EndoBarrier® devices were inserted by 23rd January 2017. Participant follow-up continues across both sites with the anticipated trial completion date being 23rd January 2019.

For peer review only

Figure 1. EndoBarrier Gastrointestinal Bypass Liner

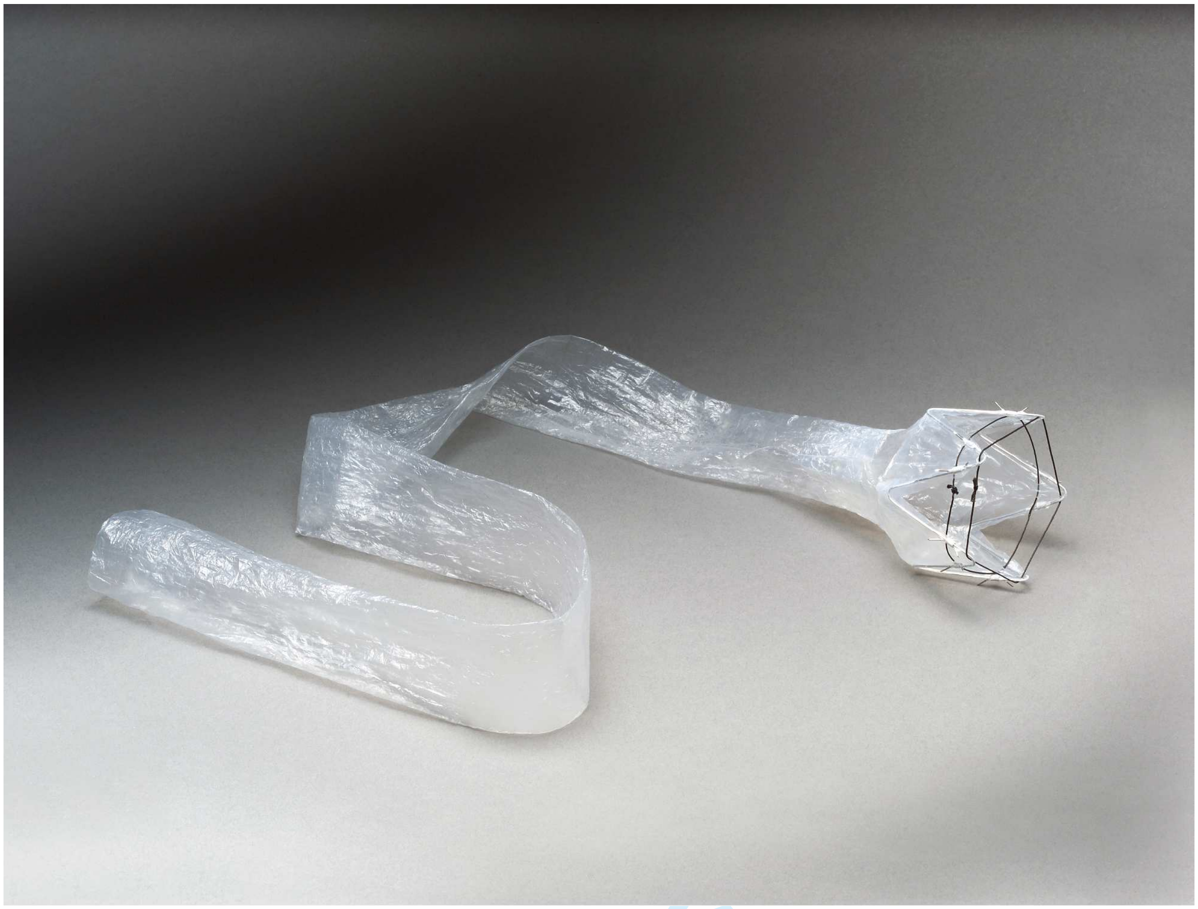

For peer review only

Figure 2a. Study interventions and follow-up schedule

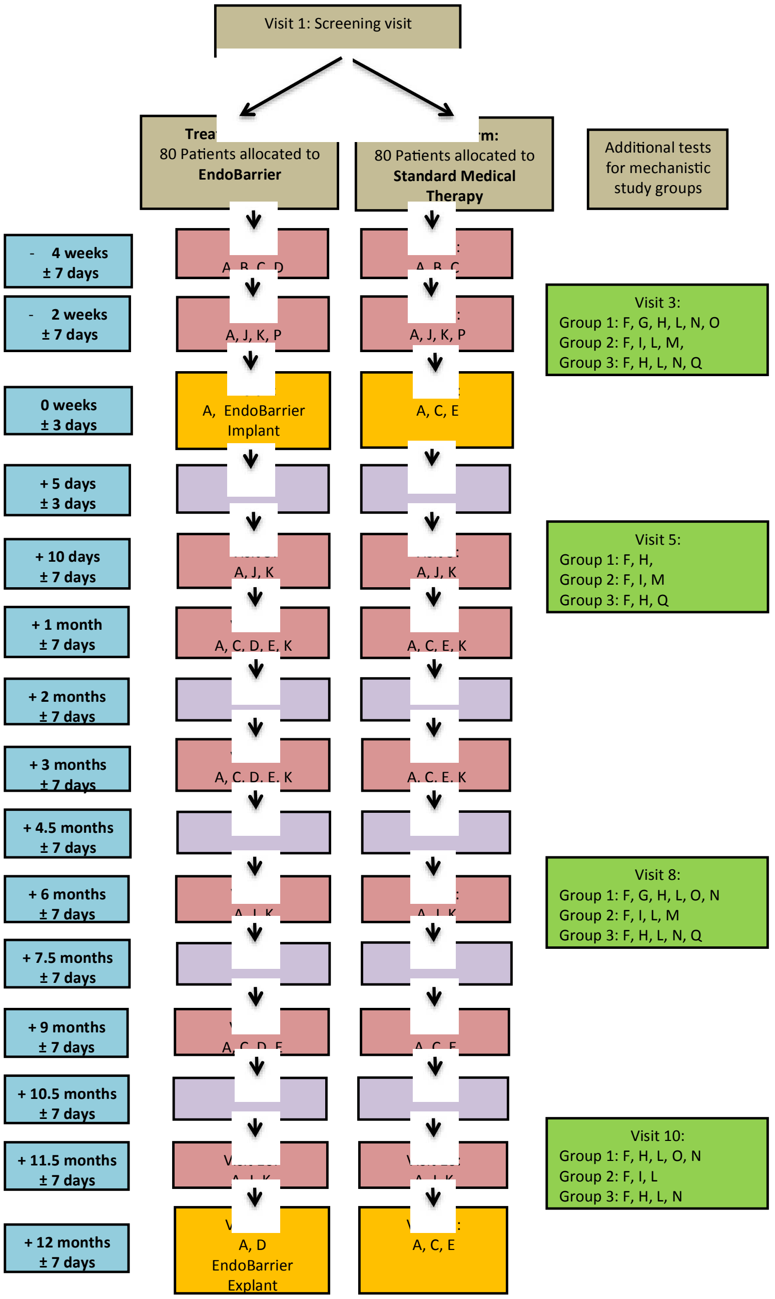

Figure 2b. Study interventions and follow-up schedule

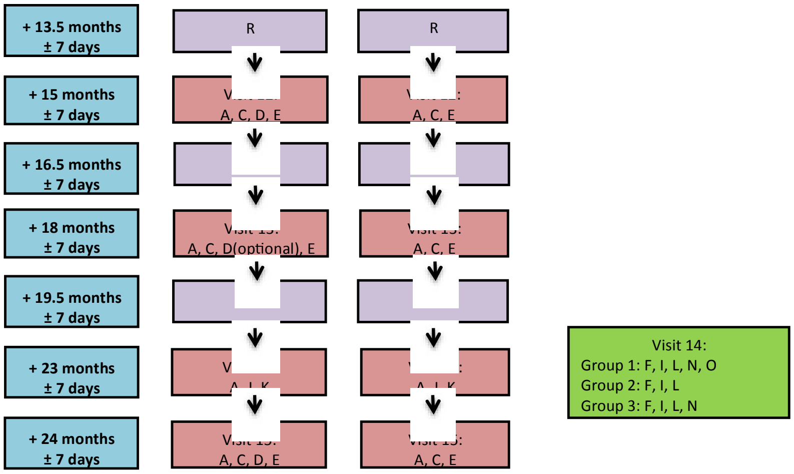

Legend:
A = Weight, waist, blood pressure, routine bloods, adverse events, changes in medication/medical history
B = Dietary Counselling
C = Medical Therapy (Diabetologist/Endocrinologist)
D = Gastroenterologist
E = Dietitian follow up
F= Bioelectrical impedance
G= fMRI
H = Gut hormones(fasting and post-meal profile)
I= Gut hormones(fasting only)
J = Metabolomics
K = Health Economics questionnaires
L = Eating and behaviour questionnaires
M = Insulin clamps
N= Eating behaviour computerised tasks
O= Cognitive assessment tasks
P= DNA Sample
Q= Food preference and taste assessment
R= Telephone counselling

only

References

1. World Health Organisation. *Obesity and overweight Fact sheet*: World Health Organisation; 2016. Available from: http://www.who.int/mediacentre/factsheets/fs311/en/. Accessed 2nd February 2017
2. Public Health England. *Adult obesity and type 2 diabetes.* PHE publications gateway number: 2014211. London: Public Health England; 2014.
3. The National Cardiovascular Intelligence Network. *Cardiovascular disease key facts. Fact sheet number 8.* PHE publications gateway number: 2013_237. London: Public Health England; 2013.
4. Health and Social Care Information Centre. *National Diabetes Audit 2012-2013, Report 2: Complications and Mortality.* Leeds: Health and Social Care Information Centre; 2015.
5. Wang YC, McPherson K, Marsh T, et al. Health and economic burden of the projected obesity trends in the USA and the UK. *The Lancet*. 2011;378(9793):815-25.
6. Wellen KE, Hotamisligil GS. Inflammation, stress, and diabetes. *J Clin Invest*. 2005;115(5):1111-9.
7. Kershaw EE, Flier JS. Adipose tissue as an endocrine organ. *J Clin Endocrinol Metab*. 2004;89(6):2548-56.
8. Kahn SE, Hull RL, Utzschneider KM. Mechanisms linking obesity to insulin resistance and type 2 diabetes. *Nature*. 2006;444(7121):840-6.
9. Boden G. Obesity, Insulin Resistance and Free Fatty Acids. *Curr Opin Endocrinol Diabetes Obes*. 2011;18(2):139 - 43.
10. Roden M, Price TB, Perseghin G, et al. Mechanism of Free Fatty Acid–induced Insulin Resistance in Humans. *J Clin Invest*. 1996;97(12):2859 - 65.
11. Karpe F, Dickmann JR, Frayn KN. Fatty Acids, Obesity, and Insulin Resistance: Time for a Reevaluation. *Diabetes*. 2011;60(10):2441 - 9
12. Mingrone G, Panunzi S, De Gaetano A, et al. Bariatric Surgery versus Conventional Medical Therapy for Type 2 Diabetes. *N Engl J Med*. 2012;366:1577 - 85.
13. Buchwald H, Estok R, Fahrbach K, et al. Weight and type 2 diabetes after bariatric surgery: systematic review and meta-analysis. *Am J Med*. 2009;122(3):248-56 e5.
14. Rubino F, Schauer PR, Kaplan LM, et al. Metabolic surgery to treat type 2 diabetes: clinical outcomes and mechanisms of action. *Annu Rev Med*. 2010;61:393-411.
15. Dixon JB. Obesity and diabetes: the impact of bariatric surgery on type-2 diabetes. *World J Surg*. 2009;33(10):2014-21.
16. Sjöström L, Lindroos A-K, Peltonen M, et al. Lifestyle, Diabetes, and Cardiovascular Risk Factors 10 Years after Bariatric Surgery. *N Engl J Med*. 2004;351:2683 - 93.
17. Dixon JB, le Roux CW, Rubino F, et al. Bariatric surgery for type 2 diabetes. *The Lancet*. 2012;379(9833):2300-11.
18. Schauer PR, Bhatt DL, Kirwan JP, et al. Bariatric Surgery versus Intensive Medical Therapy for Diabetes - 5-Year Outcomes. *N Engl J Med*. 2017;376(7):641-51.
19. Liang Z, Wu Q, Chen B, et al. Effect of laparoscopic Roux-en-Y gastric bypass surgery on type 2 diabetes mellitus with hypertension: a randomized controlled trial. *Diabetes Res Clin Pract*. 2013;101(1):50-6.
20. Gumbs AA, Modlin IM, Ballantyne GH. Changes in Insulin Resistance Following Bariatric Surgery: Role of Caloric Restriction and Weight Loss. *Obes Surg*. 2005;15(4):462 - 73.
21. Rubino F, Forgione A, Cummings DE, et al. The mechanism of diabetes control after gastrointestinal bypass surgery reveals a role of the proximal small intestine in the pathophysiology of type 2 diabetes. *Ann Surg*. 2006;244(5):741-9.
22. Cummings DE. Endocrine mechanisms mediating remission of diabetes after gastric bypass surgery. *Int J Obes (Lond)*. 2009;33 Suppl 1:S33-40.
23. Pacheco D, de Luis DA, Romero A, et al. The effects of duodenal-jejunal exclusion on hormonal regulation of glucose metabolism in Goto-Kakizaki rats. *Am J Surg*. 2007;194(2):221-4.
24. Cohen RV, Schiavon CA, Pinheiro JS, et al. Duodenal-jejunal bypass for the treatment of type 2 diabetes in patients with body mass index of 22-34 kg/m2: a report of 2 cases. *Surg Obes Relat Dis*. 2007;3(2):195-7.

For peer review only

25. Thaler JP, Cummings DE. Minireview: Hormonal and metabolic mechanisms of diabetes remission after gastrointestinal surgery. *Endocrinology*. 2009;150(6):2518-25.
26. Cummings DE, Overduin J, Shannon MH, et al. Hormonal mechanisms of weight loss and diabetes resolution after bariatric surgery. *Surg Obes Relat Dis*. 2005;1(3):358-68.
27. Shuang J, Zhang Y, Ma L, et al. Relief of diabetes by duodenal-jejunal bypass sleeve implantation in the high-fat diet and streptozotocin-induced diabetic rat model is associated with an increase in GLP-1 levels and the number of GLP-1-positive cells. *Exp Ther Med*. 2015;10(4):1355-63.
28. Knop FK. Resolution of type 2 diabetes following gastric bypass surgery: involvement of gut-derived glucagon and glucagonotropic signalling? *Diabetologia*. 2009;52(11):2270-6.
29. de Jonge C, Rensen SS, Verdam FJ, et al. Impact of Duodenal-Jejunal Exclusion on Satiety Hormones. *Obes Surg*. 2016;26(3):672-8.
30. de Jonge C, Rensen SS, Verdam FJ, et al. Endoscopic duodenal-jejunal bypass liner rapidly improves type 2 diabetes. *Obes Surg*. 2013;23(9):1354-60.
31. Munoz R, Escalona A. Duodenal-jejunal bypass liner to treat type 2 diabetes mellitus in morbidly obese patients. *Curr Cardiol Rep*. 2014;16(3):454.
32. Scholtz S, Miras AD, Chhina N, et al. Obese patients after gastric bypass surgery have lower brain-hedonic responses to food than after gastric banding. *Gut*. 2014;63(6):891-902.
33. Miras AD, Jackson RN, Jackson SN, et al. Gastric bypass surgery for obesity decreases the reward value of a sweet-fat stimulus as assessed in a progressive ratio task. *Am J Clin Nutr*. 2012;96(3):467-73.
34. Goldstone A, Miras A, Scholtz S, et al. Link Between Increased Satiety Gut Hormones and Reduced Food Reward After Gastric Bypass Surgery for Obesity. *J Clin Endocrinol Metab*. 2016;101(2):599 - 609.
35. Ochner CN, Stice E, Hutchins E, et al. Relation between changes in neural responsivity and reductions in desire to eat high-calorie foods following gastric bypass surgery. *Neuroscience*. 2012;209:128-35.
36. Milone L, Gagner M, Ueda K, et al. Effect of a Polyethylene Endoluminal Duodeno- Jejunal Tube (EDJT) on Weight Gain: A Feasibility Study in a Porcine Model. *Obes Surg*. 2006;16:620 - 6.
37. Koehestanie P, de Jonge C, Berends FJ, et al. The effect of the endoscopic duodenal-jejunal bypass liner on obesity and type 2 diabetes mellitus, a multicenter randomized controlled trial. *Ann Surg*. 2014;260(6):984-92.
38. Schouten R, Rijs CS, Bouvy ND, et al. A multicenter, randomized efficacy study of the EndoBarrier Gastrointestinal Liner for presurgical weight loss prior to bariatric surgery. *Ann Surg*. 2010;251(2):236-43.
39. Gersin KS, Rothstein RI, Rosenthal RJ, et al. Open-label, sham-controlled trial of an endoscopic duodenojejunal bypass liner for preoperative weight loss in bariatric surgery candidates. *Gastrointest Endosc*. 2010;71(6):976-82.
40. Tarnoff M, Rodriguez L, Escalona A, et al. Open label, prospective, randomized controlled trial of an endoscopic duodenal-jejunal bypass sleeve versus low calorie diet for pre-operative weight loss in bariatric surgery. *Surg Endosc*. 2009;23(3):650-6.
41. Rodriguez L, Reyes E, Fagalde P, et al. Pilot clinical study of an endoscopic, removable duodenal-jejunal bypass liner for the treatment of type 2 diabetes. *Diabetes Technol Ther*. 2009;11(11):725 - 32.
42. Rodriguez-Grunert L, Galvao Neto MP, Alamo M, et al. First human experience with endoscopically delivered and retrieved duodenal-jejunal bypass sleeve. *Surg Obes Relat Dis*. 2008;4(1):55-9.
43. Escalona A, Yanez R, Pimentel F, et al. Initial human experience with restrictive duodenal-jejunal bypass liner for treatment of morbid obesity. *Surg Obes Relat Dis*. 2010;6(2):126-31.
44. de Moura EGH, Martins BC, Lopes GS, et al. Metabolic improvements in obese type 2 diabetes subjects implanted for 1 year with an endoscopically deployed duodenal-jejunal bypass liner. *Diabetes Technol Ther*. 2012;14(2):183 - 9.
45. Escalona A, Pimentel F, Sharp A, et al. Weight loss and metabolic improvement in morbidly obese subjects implanted for 1 year with an endoscopic duodenal-jejunal bypass liner. *Ann Surg*. 2012;255(6):1080-5.

For peer review only

46. Cohen R, le Roux CW, Papamargaritis D, et al. Role of proximal gut exclusion from food on glucose homeostasis in patients with Type 2 diabetes. *Diabet Med*. 2013;30(12):1482-6.
47. Koehestanie P, Betzel B, Dogan K, et al. The feasibility of delivering a duodenal-jejunal bypass liner (EndoBarrier) endoscopically with patients under conscious sedation. *Surg Endosc*. 2014;28(1):325-30.
48. Munoz R, Dominguez A, Munoz F, et al. Baseline glycated hemoglobin levels are associated with duodenal-jejunal bypass liner-induced weight loss in obese patients. *Surg Endosc*. 2014;28(4):1056-62.
49. Force ABET, Committee AT, Abu Dayyeh BK, et al. ASGE Bariatric Endoscopy Task Force systematic review and meta-analysis assessing the ASGE PIVI thresholds for adopting endoscopic bariatric therapies. *Gastrointest Endosc*. 2015;82(3):425-38 e5.
50. Rohde U, Hedback N, Gluud LL, et al. Effect of the EndoBarrier Gastrointestinal Liner on obesity and type 2 diabetes: a systematic review and meta-analysis. *Diabetes Obes Metab*. 2016;18(3):300-5.
51. Dixon JB, Zimmet P, Alberti K, et al. Bariatric Surgery: an IDF statement for obese Type 2 diabetes. *Diabet Med*. 2011;28:628 - 42.
52. Association AD. *Standards of Medical Care in Diabetes - 2016.* 2016.
53. Inzucchi S, Bergenstal R, Buse J, et al. Management of Hyperglycemia in Type 2 Diabetes: A Patient-Centered Approach. *Diabetes Care*. 2012;35(6):1364-79.
54. Craig CL, Marshall AL, Sjostrom M, et al. International physical activity questionnaire: 12-country reliability and validity. *Med Sci Sports Exerc*. 2003;35(8):1381-95.
55. Clarke P, Gray A, Holman R. Estimating Utility Values for Health States of Type 2 Diabetic Patients Using the EQ-5D (UKPDS 62). *Med Decis Making*. 2002;22(4):340 - 9.
56. Williams JG, Alam MF, Alrubaiy L, et al. Infliximab versus ciclosporin for steroid-resistant acute severe ulcerative colitis (CONSTRUCT): a mixed methods, open-label, pragmatic randomised trial. *The Lancet Gastroenterology & Hepatology*. 2016;1(1):15-24.
57. Chernyak N, Ernsting C, Icks A. Pre-test of questions on health-related resource use and expenditure, using behaviour coding and cognitive interviewing techniques. *BMC Health Serv Res*. 2012;12:303.
58. Petersen E, Zimine I, Ho Y, et al. Non-invasive measurement of perfusion: a critical review of arterial spin labelling techniques. *Br J Radiol*. 2006;79(944):688 - 701.
59. Paiva FF, Tannus A, Silva AC. Measurement of cerebral perfusion territories using arterial spin labelling. *NMR Biomed*. 2007;20(7):633-42.
60. Damoiseaux JS, Rombouts SA, Barkhof F, et al. Consistent resting-state networks across healthy subjects. *Proc Natl Acad Sci U S A*. 2006;103(37):13848-53.
61. Goldstone A, Prechtl de Hernandez C, Beaver J, et al. Fasting biases brain reward systems towards high-calorie foods. *Eur J Neurosci*. 2009;30(8):1625 - 35.
62. Byrne CS, Chambers ES, Alhabeeb H, et al. Increased colonic propionate reduces anticipatory reward responses in the human striatum to high-energy foods. *Am J Clin Nutr*. 2016;104(1):5-14.
63. Goldstone AP, Prechtl CG, Scholtz S, et al. Ghrelin mimics fasting to enhance human hedonic, orbitofrontal cortex, and hippocampal responses to food. *Am J Clin Nutr*. 2014;99(6):1319-30.
64. Knutson B, Adams C, Fong G, et al. Anticipation of Increasing Monetary Reward Selectively Recruits Nucleus Accumbens. *J Neurosci*. 2001;21(16):RC159.
65. Knutson B, Westdorp A, Kaiser E, et al. FMRI visualization of brain activity during a monetary incentive delay task. *Neuroimage*. 2000;12(1):20-7.
66. McGonigle J, Murphy A, Paterson LM, et al. The ICCAM platform study: An experimental medicine platform for evaluating new drugs for relapse prevention in addiction. Part B: fMRI description. *J Psychopharmacol*. 2017;31(1):3-16.
67. Perry JL, Carroll ME. The role of impulsive behavior in drug abuse. *Psychopharmacology (Berl)*. 2008;200(1):1-26.
68. Horn NR, Dolan M, Elliott R, et al. Response inhibition and impulsivity: an fMRI study. *Neuropsychologia*. 2003;41(14):1959-66.
69. Li CS, Yan P, Chao HH, et al. Error-specific medial cortical and subcortical activity during the stop signal task: a functional magnetic resonance imaging study. *Neuroscience*. 2008;155(4):1142-51.

For peer review only

70. Kirby K, Maraković N. Delay-discounting probabilistic rewards: Rates decrease as amounts increase. *Psychonomic Bulletin & Review*. 1996;3(1):100 - 4.
71. Finlayson G, King N, Blundell J. The role of implicit wanting in relation to explicit liking and wanting for food: implications for appetite control. *Appetite*. 2008;50(1):120-7.
72. Bueter M, Miras AD, Chichger H, et al. Alterations of sucrose preference after Roux-en-Y gastric bypass. *Physiol Behav*. 2011;104(5):709-21.
73. Gæde P, Lund-Andersen H, Parving H, et al. Effect of a Multifactorial Intervention on Mortality in Type 2 Diabetes. *N Engl J Med*. 2008;358:580 - 91.
74. Devlin N, Shah K, Feng Y, et al. Economics OoH. *Valuing Health-Related Quality of Life: An EQ-5D-5L Value Set for England.* . 2016.
75. Philips Z, Ginnelly L, Sculpher M, et al. Review of guidelines for good practice in decision-analytic modelling in health technology assessment. *Health technology assessment*. 2004;8(36):iii-iv, ix-xi, 1-158.
76. Caro JJ, Briggs AH, Siebert U, et al. Modeling good research practices--overview: a report of the ISPOR-SMDM Modeling Good Research Practices Task Force-1. *Medical decision making : an international journal of the Society for Medical Decision Making*. 2012;32(5):667-77.

For peer review only

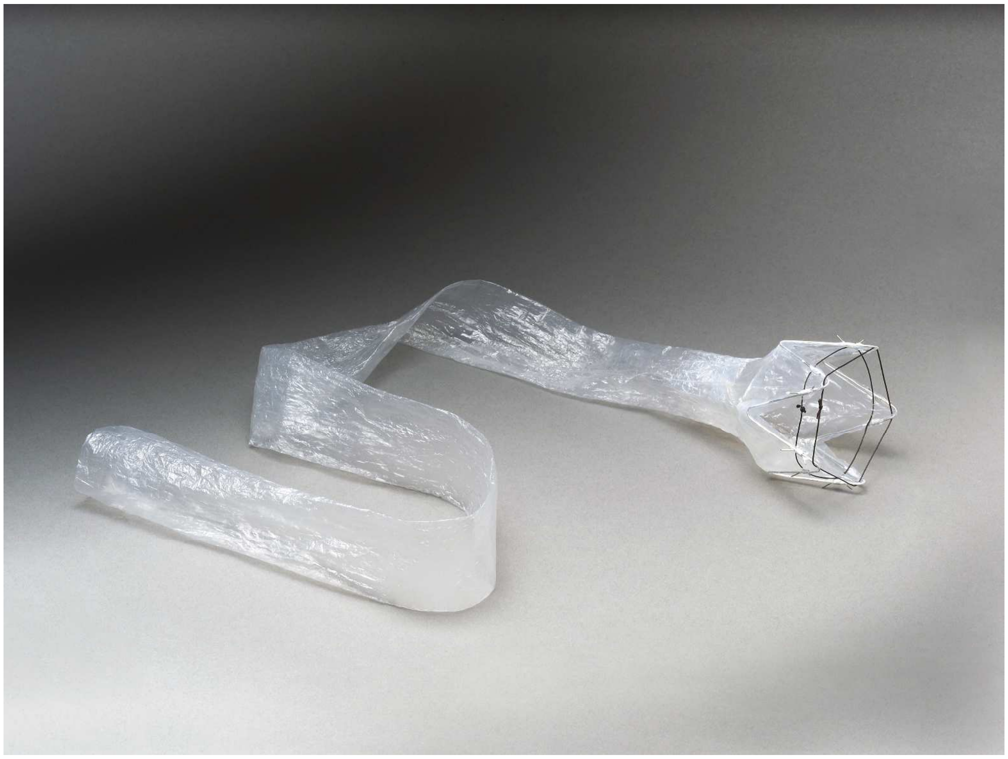

Figure 1. EndoBarrier Gastrointestinal Bypass Liner

344x258mm (300 x 300 DPI)

For peer review only - http://bmjopen.bmj.com/site/about/guidelines.xhtml

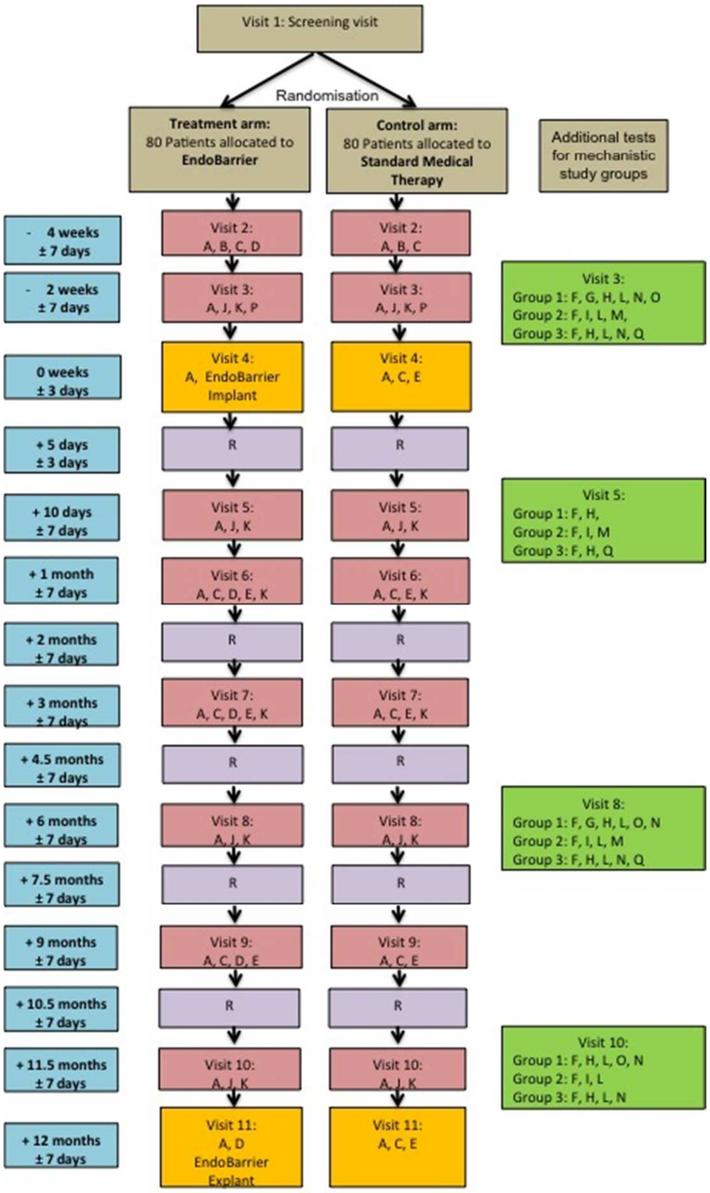

Figure 2a. Study interventions and follow-up schedule

161x275mm (72 x 72 DPI)

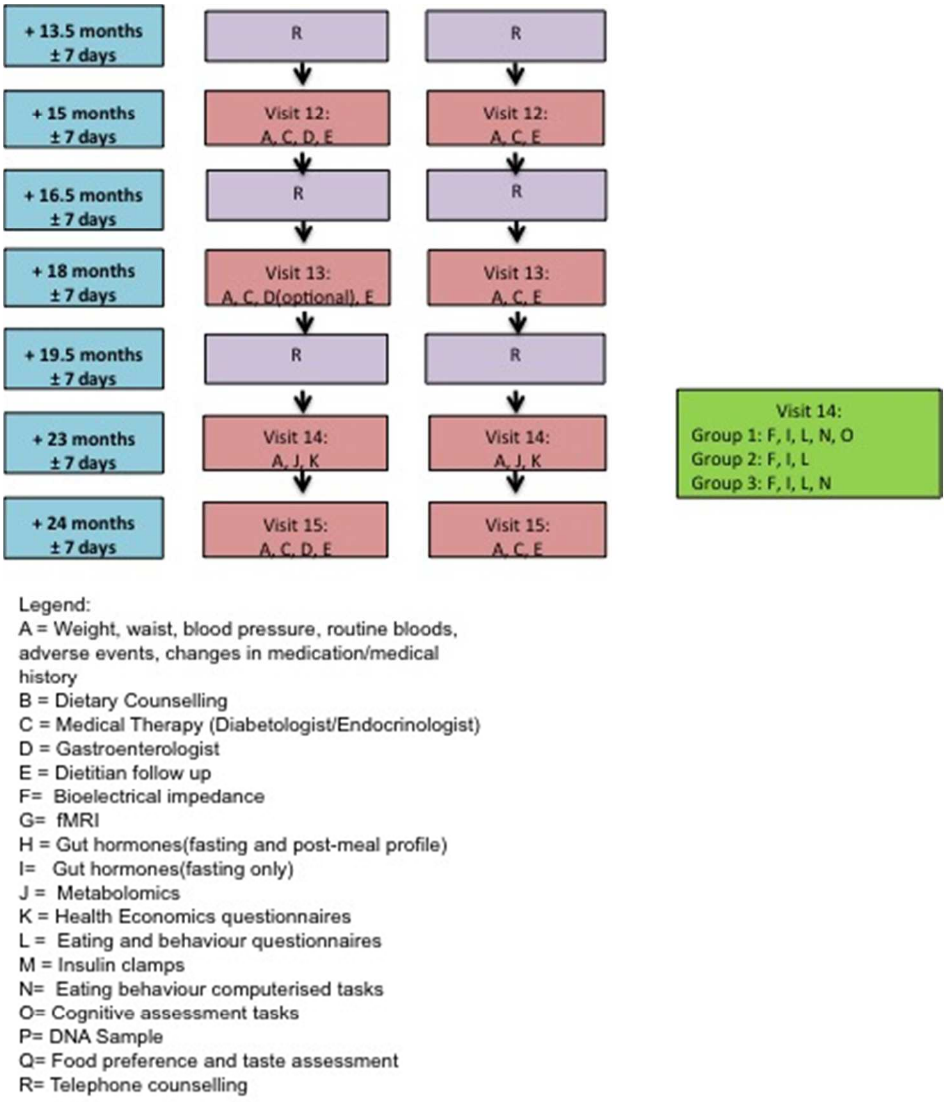

Figure 2b. Study interventions and follow-up schedule

165x197mm (72 x 72 DPI)

SPIRIT

STANDARD PROTOCOL ITEMS: RECOMMENDATIONS FOR INTERVENTIONAL TRIALS

SPIRIT 2013 Checklist: Recommended items to address in a clinical trial protocol and related documents*

Section/item	Item No	Description	Addressed on page number
**Administrative information**			
Title	1	Descriptive title identifying the study design, population, interventions, and, if applicable, trial acronym	______1_______
Trial registration	2a	Trial identifier and registry name. If not yet registered, name of intended registry	______4_______
	2b	All items from the World Health Organization Trial Registration Data Set	https://clinicaltrials.gov/ct2/show/record/NCT02459561
Protocol version	3	Date and version identifier	______8_______
Funding	4	Sources and types of financial, material, and other support	______5______
Roles and responsibilities	5a	Names, affiliations, and roles of protocol contributors	_____1-3______
	5b	Name and contact information for the trial sponsor	______5______
	5c	Role of study sponsor and funders, if any, in study design; collection, management, analysis, and interpretation of data; writing of the report; and the decision to submit the report for publication, including whether they will have ultimate authority over any of these activities	______5_______

For peer review only - http://bmjopen.bmj.com/site/about/guidelines.xhtml

	5d	Composition, roles, and responsibilities of the coordinating centre, steering committee, endpoint adjudication committee, data management team, and other individuals or groups overseeing the trial, if applicable (see Item 21a for data monitoring committee)	______8_______
**Introduction**			
Background and rationale	6a	Description of research question and justification for undertaking the trial, including summary of relevant studies (published and unpublished) examining benefits and harms for each intervention	____9-10______
	6b	Explanation for choice of comparators	___ 9-12______
Objectives	7	Specific objectives or hypotheses	____10-11______
Trial design	8	Description of trial design including type of trial (eg, parallel group, crossover, factorial, single group), allocation ratio, and framework (eg, superiority, equivalence, noninferiority, exploratory)	_____11________
**Methods: Participants, interventions, and outcomes**			
Study setting	9	Description of study settings (eg, community clinic, academic hospital) and list of countries where data will be collected. Reference to where list of study sites can be obtained	__11, 16-17____
Eligibility criteria	10	Inclusion and exclusion criteria for participants. If applicable, eligibility criteria for study centres and individuals who will perform the interventions (eg, surgeons, psychotherapists)	_____16_______
Interventions	11a	Interventions for each group with sufficient detail to allow replication, including how and when they will be administered	___17-19______
	11b	Criteria for discontinuing or modifying allocated interventions for a given trial participant (eg, drug dose change in response to harms, participant request, or improving/worsening disease)	____17-19_____ Main protocol Section 7
	11c	Strategies to improve adherence to intervention protocols, and any procedures for monitoring adherence (eg, drug tablet return, laboratory tests)	_____27______

For peer review only - http://bmjopen.bmj.com/site/about/guidelines.xhtml

	11d	Relevant concomitant care and interventions that are permitted or prohibited during the trial	_____16______
Outcomes	12	Primary, secondary, and other outcomes, including the specific measurement variable (eg, systolic blood pressure), analysis metric (eg, change from baseline, final value, time to event), method of aggregation (eg, median, proportion), and time point for each outcome. Explanation of the clinical relevance of chosen efficacy and harm outcomes is strongly recommended	__10-11, 19-23___
Participant timeline	13	Time schedule of enrolment, interventions (including any run-ins and washouts), assessments, and visits for participants. A schematic diagram is highly recommended (see Figure)	__13-15, 29-30__
Sample size	14	Estimated number of participants needed to achieve study objectives and how it was determined, including clinical and statistical assumptions supporting any sample size calculations	_____23_______
Recruitment	15	Strategies for achieving adequate participant enrolment to reach target sample size	____16-17____
**Methods: Assignment of interventions (for controlled trials)**			
Allocation:			
Sequence generation	16a	Method of generating the allocation sequence (eg, computer-generated random numbers), and list of any factors for stratification. To reduce predictability of a random sequence, details of any planned restriction (eg, blocking) should be provided in a separate document that is unavailable to those who enrol participants or assign interventions	_____17______
Allocation concealment mechanism	16b	Mechanism of implementing the allocation sequence (eg, central telephone; sequentially numbered, opaque, sealed envelopes), describing any steps to conceal the sequence until interventions are assigned	_____17______
Implementation	16c	Who will generate the allocation sequence, who will enrol participants, and who will assign participants to interventions	_____17______
Blinding (masking)	17a	Who will be blinded after assignment to interventions (eg, trial participants, care providers, outcome assessors, data analysts), and how	_____N/A______
	17b	If blinded, circumstances under which unblinding is permissible, and procedure for revealing a participant's allocated intervention during the trial	_____N/A______

Methods: Data collection, management, and analysis

Data collection methods	18a	Plans for assessment and collection of outcome, baseline, and other trial data, including any related processes to promote data quality (eg, duplicate measurements, training of assessors) and a description of study instruments (eg, questionnaires, laboratory tests) along with their reliability and validity, if known. Reference to where data collection forms can be found, if not in the protocol	___19-23____
	18b	Plans to promote participant retention and complete follow-up, including list of any outcome data to be collected for participants who discontinue or deviate from intervention protocols	___23-24, 27____
Data management	19	Plans for data entry, coding, security, and storage, including any related processes to promote data quality (eg, double data entry; range checks for data values). Reference to where details of data management procedures can be found, if not in the protocol	____19, 27_____ Main protocol Section 9, 11.9 & 12
Statistical methods	20a	Statistical methods for analysing primary and secondary outcomes. Reference to where other details of the statistical analysis plan can be found, if not in the protocol	___23-25____
	20b	Methods for any additional analyses (eg, subgroup and adjusted analyses)	____23-25___
	20c	Definition of analysis population relating to protocol non-adherence (eg, as randomised analysis), and any statistical methods to handle missing data (eg, multiple imputation)	___23-25____

Methods: Monitoring

Data monitoring	21a	Composition of data monitoring committee (DMC); summary of its role and reporting structure; statement of whether it is independent from the sponsor and competing interests; and reference to where further details about its charter can be found, if not in the protocol. Alternatively, an explanation of why a DMC is not needed	_____8_____ Main protocol Section 12
	21b	Description of any interim analyses and stopping guidelines, including who will have access to these interim results and make the final decision to terminate the trial	____N/A______
Harms	22	Plans for collecting, assessing, reporting, and managing solicited and spontaneously reported adverse events and other unintended effects of trial interventions or trial conduct	____19_____ Main protocol section 6

For peer review only - http://bmjopen.bmj.com/site/about/guidelines.xhtml

Auditing	23	Frequency and procedures for auditing trial conduct, if any, and whether the process will be independent from investigators and the sponsor	____8, 27____ Main protocol section 12
**Ethics and dissemination**			
Research ethics approval	24	Plans for seeking research ethics committee/institutional review board (REC/IRB) approval	_____N/A_____
Protocol amendments	25	Plans for communicating important protocol modifications (eg, changes to eligibility criteria, outcomes, analyses) to relevant parties (eg, investigators, REC/IRBs, trial participants, trial registries, journals, regulators)	____N/A_____
Consent or assent	26a	Who will obtain informed consent or assent from potential trial participants or authorised surrogates, and how (see Item 32)	_____17_____ Main protocol section 11.5
	26b	Additional consent provisions for collection and use of participant data and biological specimens in ancillary studies, if applicable	_____17_____ Main protocol section 11.5
Confidentiality	27	How personal information about potential and enrolled participants will be collected, shared, and maintained in order to protect confidentiality before, during, and after the trial	_____5______ Main protocol section 11
Declaration of interests	28	Financial and other competing interests for principal investigators for the overall trial and each study site	_____5-6______
Access to data	29	Statement of who will have access to the final trial dataset, and disclosure of contractual agreements that limit such access for investigators	_____5______ Main protocol section 12
Ancillary and post-trial care	30	Provisions, if any, for ancillary and post-trial care, and for compensation to those who suffer harm from trial participation	____N/A_____

For peer review only

For peer review only - http://bmjopen.bmj.com/site/about/guidelines.xhtml

Dissemination policy	31a	Plans for investigators and sponsor to communicate trial results to participants, healthcare professionals, the public, and other relevant groups (eg, via publication, reporting in results databases, or other data sharing arrangements), including any publication restrictions	____N/A_____ Main protocol section 12.8
	31b	Authorship eligibility guidelines and any intended use of professional writers	____N/A______
	31c	Plans, if any, for granting public access to the full protocol, participant-level dataset, and statistical code	____N/A_____
**Appendices**			
Informed consent materials	32	Model consent form and other related documentation given to participants and authorised surrogates	_____N/A_____
Biological specimens	33	Plans for collection, laboratory evaluation, and storage of biological specimens for genetic or molecular analysis in the current trial and for future use in ancillary studies, if applicable	_____N/A_____

*It is strongly recommended that this checklist be read in conjunction with the SPIRIT 2013 Explanation & Elaboration for important clarification on the items. Amendments to the protocol should be tracked and dated. The SPIRIT checklist is copyrighted by the SPIRIT Group under the Creative Commons "Attribution-NonCommercial-NoDerivs 3.0 Unported" license.

For peer review only

BMJ Open

Study Protocol: A randomized controlled trial of a duodenal-jejunal bypass sleeve device (EndoBarrier®) compared with standard medical therapy for the management of obese subjects with type 2 diabetes mellitus.

Journal:	*BMJ Open*
Manuscript ID	bmjopen-2017-018598.R1
Article Type:	Protocol
Date Submitted by the Author:	18-Sep-2017

Complete List of Authors:	Glaysher, Michael; University Hospital Southampton, Southamptom Biomedical Research Centre<br>Mohanaruban, Aruchuna; Imperial College Healthcare NHS Trust, St. Mary's Hospital, London, W2 1NY<br>Prechtl, Christina; Imperial College London, Department of Public Health, Imperial Clinical Trials Unit, Stadium House, 68 Wood Lane, London, W12 7RH<br>Goldstone, Anthony; Imperial College London, PsychoNeuroEndocrinology Research Group, Neuropsychopharmacology Unit, Centre for Psychiatry and Computational, Cognitive and Clinical Neuroimaging Laboratory, Division of Brain Sciences, Hammersmith Hospital<br>Miras , Alexander ; Imperial College Healthcare NHS Trust, Division of Diabetes, Endocrinology and Metabolic Medicine, 6th floor Commonwealth Building, Hammersmith Hospital, London W12 0NN<br>Lord, Joanne; University of Southampton, Southampton Health Technology Assessments Centre, University of Southampton, Alpha House, University of Southampton Science Park, Southampton SO16 7NS<br>Chhina, Navpreet; Imperial College Healthcare NHS Trust, Computational Cognitive and Clinical Neuroimaging Group, Burlington Danes Building, Hammersmith Hospital, Imperial College London, Du Cane Road, London, W12 0NN<br>Falaschetti, Emanuela; Imperial College London, Imperial Clinical Trials Unit, Imperial College London, Stadium House, 68 Wood Lane, London, W12 7RH.<br>Johnson, Nicholas; Imperial College London, School of Public Health, 68 Wood Lane, London, W12 7RH<br>Al-Najim, Werd ; Imperial College London, Department of Investigative Medicine, Imperial College London, London, United Kingdom; University College Dublin, Diabetes Complications Research Centre, Conway Institute, School of Medicine and Medical Sciences<br>Smith, Claire; Imperial College London, Imperial Clinical Trials Unit, 68 Wood Lane, London, W12 7RH<br>Li, Jia ; Imperial College London, Division of Computational and Systems Medicine, Department of Surgery and Cancer, Faculty of Medicine, Imperial College London, Alexander Fleming Building, Imperial College Road, South Kensington, London, SW7 2AZ<br>Patel, Mayank; University Hospital Southampton, Department of Diabetes and Endocrinology, Room CE145, Mailpoint 134, Southampton General Hospital, Tremona Road, Southampton, SO16 6DD

For peer review only - http://bmjopen.bmj.com/site/about/guidelines.xhtml

Ahmed, Ahmed; Imperial College Healthcare NHS Trust, Consultant Upper GI and Bariatric Surgeon, Clinical Lead – Bariatric Surgery, St Mary’s Hospital, Imperial College Healthcare NHS Trust, Room 35, 4th Floor, Salton House, St Mary’s Hospital, Praed Street, London, W2 1NY.
Moore, Michael; University of Southampton Medical School, Primary Care Medical Group
Poulter, Neil; Imperial College London, Imperial Clinical Trials Unit, 1st Floor Stadium House, 68 Wood Lane, London W12 7TA
Bloom, Stephen; Imperial College London, Clinical Director, North West London Pathology, Head of Division of Diabetes, Endocrinology and Metabolism, Imperial College, 6th Floor Commonwealth Building, Hammersmith Hospital, Imperial College London, Du Cane Road, London, W12 0NN
Darzi, Ara; Imperial College London, Institute of Global Health Innovation
Le Roux, Carel; University College Dublin
Byrne, James; University Hospital Southampton, Division of Surgery, University Hospital Southampton NHS Foundation Trust, Tremona Road, Southampton, Hampshire, SO16 6YD
Teare, Julian; Imperial College Healthcare NHS Trust, Department of Gastroenterology, St. Mary's Hospital, Praed Street, London, W2 1NY

**Primary Subject Heading**:	Diabetes and endocrinology
Secondary Subject Heading:	Nutrition and metabolism, Surgery
Keywords:	Obesity, Type 2 diabetes mellitus, Endobarrier, Duodenal-jejunal bypass sleeve, Duodenal-jejunal bypass liner

SCHOLARONE™
Manuscripts

Study Protocol: A randomized controlled trial of a duodenal-jejunal bypass sleeve device (EndoBarrier®) compared with standard medical therapy for the management of obese subjects with type 2 diabetes mellitus.

Authors:

Mr Michael A Glaysher
michaelglaysher@me.com
Clinical Research Fellow and General Surgical Registrar, Level D, Laboratory & Pathology Block, SCBR - MP 218, University Hospital Southampton NHS Foundation Trust, Tremona Road, Southampton, Hampshire, SO16 6YD. Tel: 023 8120 5339 Mob: 07834897272
No funding or conflicts of interest to declare

Dr Aruchuna Mohanaruban
a.mohanaruban@imperial.ac.uk
Clinical research fellow and gastroenterology registrar, Imperial College Healthcare NHS Trust, St. Mary's Hospital, London, W2 1NY
No funding or conflicts of interest to declare

Dr Christina Gabriele Prechtl
c.prechtl@imperial.ac.uk
Imperial College London, Department of Public Health, Imperial Clinical Trials Unit, Stadium House, 68 Wood Lane, London, W12 7RH. Phone: +44 207 594 5946
Funding: National Institute for Health Research, No conflict of interest

Dr. Anthony P. Goldstone
tony.goldstone@imperial.ac.uk
PsychoNeuroEndocrinology Research Group, Neuropsychopharmacology Unit, Centre for Psychiatry and Computational, Cognitive and Clinical Neuroimaging Laboratory, Division of Brain Sciences, Imperial College London, Hammersmith Hospital, London, UK. Tel: + 44 20 7594 5989
No competing interests. Funding supported by UK Medical Research Council and Wellcome Trust

Dr Alexander Dimitri Miras
a.miras@nhs.net
Senior Clinical Lecturer in Endocrinology, Division of Diabetes, Endocrinology and Metabolic Medicine, 6th floor Commonwealth Building, Hammersmith Hospital, London W12 0NN. Tel: +44 (0)7958 377674. Fax: +44 (0)208 3838320
Funding: MRC, NIHR, Moulton Charitable Foundation, Fractyl, Novo Nordisk, Astra Zeneka

Professor Joanne Lord
j.lord@soton.ac.uk
Southampton HTA Centre, University of Southampton, Alpha House, University of Southampton Science Park, Southampton SO16 7NS. Tel: +44(0)23 8059 3749
Prof. Lord reports grants from Efficacy and Mechanism Evaluation (EME) Programme, an MRC and National Institute for Health Research (NIHR) partnership, and non-financial support from GI Dynamics Inc , during the conduct of the study.

Dr Navpreet Chhina
navpreet.chhina@imperial.ac.uk
Clinical Research Fellow in Endocrinology, Computational Cognitive and Clinical Neuroimaging Group, Burlington Danes Building, Hammersmith Hospital, Imperial College London, Du Cane Road, London, W12 0NN. Tel: 020 7594 5988
No funding or conflicts of interest to declare

For peer review only

Emanuela Falaschetti, CStat
e.falaschetti@imperial.ac.uk
Research Fellow in Clinical Trial Statistics, Imperial Clinical Trials Unit, Imperial College London, Stadium House, 68 Wood Lane, London, W12 7RH. Tel: 020 75941846
No funding or conflicts of interest to declare

Mr Nicholas Andrew Johnson
nicholas.johnson@imperial.ac.uk
Imperial College London, School of Public Health, 68 Wood Lane, London, W12 7RH. Tel: 020 7594 1751
No funding or conflicts of interest to declare

Dr Werd Al-Najim
w.al-najim@imperial.ac.uk
*Department of Investigative Medicine, Imperial College London, London, United Kingdom. Tel: 07590 427 587
*Diabetes Complications Research Centre, Conway Institute, School of Medicine and Medical Sciences, University College Dublin, Ireland
Dr. Al-Najim reports grants from Efficacy and Mechanism Evaluation (EME) Programme, an MRC and National Institute for Health Research (NIHR) partnership. Other support was provided from GI Dynamics.

Dr Claire Smith
claire.smith@imperial.ac.uk
Imperial College London, Imperial Clinical Trials Unit, 68 Wood Lane, London, W12 7RH. Tel: 020 7594 2101.
No funding or conflicts of interest to declare

Dr Jia V Li
jia.li@imperial.ac.uk
Division of Computational and Systems Medicine, Department of Surgery and Cancer, Faculty of Medicine, Imperial College London, Alexander Fleming Building, Imperial College Road, South Kensington, London, SW7 2AZ, UK. Phone: 02075943230
No funding or conflicts of interest to declare

Dr Mayank Patel
Mayank.Patel@uhs.nhs.uk
Consultant in Diabetes & Endocrinology, Room CE145, Mailpoint 134, Southampton General Hospital, Tremona Road, Southampton, SO16 6DD. Tel: 023 8120 4165
No funding or conflicts of interest to declare

Mr Ahmed R Ahmed
Ahmed.ahmed@imperial.nhs.uk
Consultant Upper GI and Bariatric Surgeon, Clinical Lead – Bariatric Surgery, St Mary's Hospital, Imperial College Healthcare NHS Trust, Room 35, 4th Floor, Salton House, St Mary's Hospital, Praed Street, London, W2 1NY. Tel: +44 20 8846 1081
No funding or conflicts of interest to declare

Professor Michael Moore
Mvm198@soton.ac.uk
Primary Care and Population Sciences, University of Southampton, Aldermoor Health Centre, Aldermoor Close, Southampton, Hampshire, SO16 5ST. Tel: 023 8059 1774
No funding or conflicts of interest to declare

Prof Neil Reginald Poulter

For peer review only

n.poulter@imperial.ac.uk
Imperial College London, Imperial Clinical Trials Unit, 1st Floor Stadium House, 68 Wood Lane, London W12 7TA. Tel: +44 (0)207 594 3446
Prof. Poulter's institution reports grants from HTA, during the conduct of the study.

Professor Sir Stephen Bloom
s.bloom@imperial.ac.uk
Clinical Director, North West London Pathology, Head of Division of Diabetes, Endocrinology and Metabolism, Imperial College, 6th Floor Commonwealth Building,, Hammersmith Hospital, Imperial College London, Du Cane Road, London, W12 0NN. Tel: 020 8383 3242.
No funding or conflicts of interest to declare

Professor Ara Darzi
a.darzi@imperial.ac.uk
Paul Hamlyn Chair of Surgery, Imperial College London, Division of Surgery, 10th Floor QEQM, St Mary's Hospital, South Wharf Road, London, W2 1NY. Tel: 020 3312 1310
No funding or conflicts of interest to declare

Prof Carel Le Roux
carel.leroux@ucd.ie
Imperial College London, Charing Cross Hospital, Fulham Palace Rd, London W6 8RF. Tel: +44 (0)7970 719 453
Dr. le Roux reports grants from Science Foundation Ireland, grants from Health Research Board, during the conduct of the study; other from NovoNordisk, other from GI Dynamics, personal fees from Eli Lilly, grants and personal fees from Johnson and Johnson, personal fees from Sanofi Aventis, personal fees from Astra Zeneca, personal fees from Janssen, personal fees from Bristol-Myers Squibb, personal fees from Boehringer-Ingelheim, outside the submitted work; .

Mr James P Byrne
James.byrne@uhs.nhs.uk
Division or Surgery, University Hospital Southampton NHS Foundation Trust, Tremona Road, Southampton, Hampshire, SO16 6YD. Tel: 023 8120 8535.
No funding or conflicts of interest to declare

Prof Julian Teare
j.teare@imperial.ac.uk
Imperial College Healthcare NHS Trust, Clarence Wing, St. Mary's Hospital, London, W2 1NY. Tel: +44 (0)20 3312 1072

For peer review only

Abstract

Introduction: The prevalence of obesity and obesity-related diseases, including type 2 diabetes mellitus (T2DM) is increasing. Exclusion of the foregut, as occurs in Roux-en-Y gastric bypass, has a key role in the metabolic improvements that occur following bariatric surgery, which are independent of weight-loss. Endoscopically-placed duodenal-jejunal bypass sleeve (DJBS) devices, such as the EndoBarrier® (GI Dynamics Inc, Lexington MA), have been designed to create an impermeable barrier between chyme exiting the stomach and the mucosa of the duodenum and proximal jejunum. The non-surgical and reversible nature of these devices represents an attractive therapeutic option for patients with obesity and T2DM by potentially improving glycaemic control and reducing their weight.

Methods and Analysis: In this multicentre, randomised, controlled, non-blinded trial, male and female patients aged 18–65 years with a BMI 30–50 kg/m^2 and inadequately controlled T2DM on oral anti-hyperglycaemic medications (HbA1C 58-97mmol/mol) will be randomised in a 1:1 ratio to receive either the EndoBarrier® device (n=80) for 12 months or conventional medical therapy, diet and exercise (n=80). The primary outcome measure will be a reduction in HbA1C by 20% at 12 months. Secondary outcome measures will include percentage weight loss, change in cardiovascular risk factors and medications, quality of life, cost, QALYs accrued and adverse events. Three additional sub-groups will investigate the mechanisms behind the effect of the EndoBarrier® device, looking at changes in: gut hormones, metabolites, bile acids, microbiome, food hedonics and preferences, taste, brain reward system responses to food, eating and addictive behaviours, body fat content, insulin sensitivity, and intestinal tissue gene expression.

Ethics and Dissemination: Research ethics approval was granted by Fulham Research Ethics Committee, London, (Reference 14/LO/0871) on 10th July 2014. All subjects will give informed written consent. Study findings will be disseminated through peer-reviewed journals, national and international conferences.

Strengths and Limitations of this study:

- This study will represent the largest randomised controlled trial of the EndoBarrier® device compared with conventional medical therapy, diet and exercise over a treatment period of 1 year and will also provide the longest follow-up data (1 year) of any trial to date.
- This study will provide: (1) unique data on the mechanism of action of the DJBS and the effect of foregut exclusion on an individual's metabolic profile, (2) a cost-effectiveness analysis, (3) quality of life assessment outcomes, and (4) extensive safety data.
- The unblinded design of this trial introduces the risk of bias.

Key words: Obesity, Type 2 diabetes mellitus, Endobarrier, Duodenal-jejunal bypass sleeve, Duodenal-jejunal bypass liner

Trial Registration: ISRCTN30845205, ClinicalTrials.gov Identifier NCT02459561

view only

Trial Sponsor:

Imperial College London
Faculty of Medicine Centre
Joint Research Office
Room 510B
5th Floor Lab Block
Charing Cross Hospital
Charing Cross Campus
Fulham Palace Road
W6 8RF
Tel: +44(0)203 311 0204
Fax: +44(0)203 311 0203

Contact person:
Mrs Ruth Nicholson
Tel: +44 (0)203 311 0212
Fax: +44(0)203 311 0203

Information concerning the study, patent applications, processes, scientific data or other pertinent information is confidential and remains the property of the Sponsor. Verbal or written discussion of results prior to study completion and full reporting should only be undertaken with written consent from the Sponsor.

Funding and Acknowledgements:

This project is funded by the Efficacy and Mechanism Evaluation (EME) Programme, an MRC and National Institute for Health Research (NIHR) partnership.

This paper presents independent research funded by the EME Programme and supported by the NIHR CRF and BRC at Imperial College Healthcare NHS Trust and University Hospital Southampton NHS Foundation Trust. The views expressed are those of the author(s) and not necessarily those of the EME Programme, the NHS, the NIHR or the Department of Health

This study is being executed with the support of GI Dynamics, Inc. and with kind support of Nutricia Advanced Medical Nutrition for providing oral nutritional supplements. Special thanks are given to Brunel University London and the Wolfson Centre for Translational Research, University of Surrey, for their support in sample analysis and to Professors Desmond Johnston, Jeremy Nicholson, and Elaine Holmes as co-applicants for the trial and for their valuable support in the set-up and design of this trial.

Competing interests statement:

Dr. Miras reports grants from Fractyl, personal fees from Novo Nordisk, and personal fees from Astra Zeneka outside the submitted work.

Dr. Goldstone reports funding supported by UK Medical Research Council and Wellcome Trust, outside of the submitted work.

Prof. Poulter's institution reports grants from HTA, during the conduct of the study.

Dr. le Roux reports grants from Science Foundation Ireland, grants from Health Research Board, during the conduct of the study; other from NovoNordisk, other from GI Dynamics, personal fees from Eli Lilly, grants and personal fees from Johnson and Johnson, personal fees from Sanofi Aventis, personal fees

For peer review only

from Astra Zeneca, personal fees from Janssen, personal fees from Bristol-Myers Squibb, personal fees from Boehringer-Ingelheim, outside the submitted work; .

Contributorship statement:

Mr Michael A Glaysher - Co-investigator at University Hospital Southampton. Corresponding author and primary author of manuscript.

Dr Aruchuna Mohanaruban - Co-investigator at Imperial College London. Contributed to writing manuscript and approved final version.

Dr Christina Gabriele Prechtl - Trial manager at Imperial College London, Imperial Clinical Trials Unit. Co-author of approved study protocol, contributed to writing manuscript and approved final version.

Dr. Anthony P. Goldstone - Co-investigator at Imperial College London and trial co-applicant. Co-author of approved study protocol, contributed to writing manuscript and approved final version.

Dr Alexander Dimitri Miras - Co-investigator at Imperial College London. Co-author of approved study protocol, contributed to writing manuscript and approved final version

Professor Joanne Lord - Director and Professorial Fellow in Health Economics. Co-author of approved study protocol, contributed to writing manuscript and approved final version

Dr Navpreet Chhina - Co-investigator at Imperial College London. Co-author of approved study protocol, provided critical appraisal of current manuscript and approved final version

Emanuela Falaschetti - Trial co-applicant. Trial statistician, contributed to writing manuscript and approved final version

Mr Nicholas Andrew Johnson - Trial statistician, contributed to writing manuscript and approved final version

Dr Werd Al-Najim - Co-investigator at Imperial College London. Co-author of approved study protocol, contributed to writing manuscript and approved final version

Dr Claire Smith - Operations and Business Development Manager, Imperial Clinical Trials Unit. Provided critical appraisal of current manuscript and approved final version

Dr Jia V Li - Contributed to approved study protocol, provided critical appraisal of manuscript and approved final version

Dr Mayank Patel - Co-investigator at University Hospital Southampton. Contributed to approved study protocol, provided critical appraisal of manuscript and approved final version

Mr Ahmed R Ahmed - Trial co-applicant. Contributed to trial set-up and design. Provided critical appraisal of manuscript and approved final version

Professor Michael Moore - Trial co-applicant. Contributed to trial set-up and design and was involved in the design of approved study protocol. Provided critical appraisal of manuscript and approved final version

Prof Neil Reginald Poulter - Trial co-applicant. Contributed to trial set-up and design. Provided critical appraisal of manuscript and approved final version

For peer review only

Professor Sir Stephen Bloom - Trial co-applicant. Contributed to trial set-up and design. Provided critical appraisal of manuscript and approved final version

Professor Ara Darzi - Trial co-applicant. Contributed to trial set-up and design. Provided critical appraisal of manuscript and approved final version

Prof Carel Le Roux - Trial co-applicant. Contributed to trial set-up and design. Provided critical appraisal of manuscript and approved final version

Mr James P Byrne - Principal investigator at University Hospital Southampton. Trial co-applicant, co-author for approved study protocol, provided critical appraisal of manuscript and approved final version

Prof Julian Teare - Chief investigator. Trial co-applicant, co-author for approved study protocol, provided critical appraisal of manuscript and approved final version

For peer review only

Protocol Version

Current version 4.0

Revision chronology:

- Minor amendment 3 29/09/16
  - Protocol V4.1 – number of randomised patients increased from 160 to 170
- Minor amendment 2 11/05/16
  - Extension of funding for 6 months
- Substantial amendment 10/08/15
  - Protocol V4.0 - Changes of inclusion (Section 4.2) and exclusion criteria (section 4.3)
  - Additional correction of minor transcription error in section 5.3
- Minor amendment 1 03/08/15
  - Protocol V3.1 - minor correction of inclusion criteria (section 4.2)
- Substantial amendment 16/03/15
  - Protocol V3.0 - Substantial amendment to protocol
  - Additional amendments to PIS, recruitment advert, business cards, news story.
- Substantial amendment 23/09/14
  - Protocol V2.0 - amendments to duration of liquid diet, questionnaires, blood sampling schedule, testing procedures and addition of biopsies.
  - Additional amendments to PIS, GP/consultant information sheets and letters, participant invitation letters, diet sheets, consent forms and questionnaires.
- Original submission 10/07/14
  - Protocol V1.0

Trial Steering Committee (TSC)
A Trial Steering committee (TSC) with an independent Chair will be appointed and will be responsible for overseeing the progress of the trial. A TSC Charter will be devised to list the roles and responsibilities of the TSC members. TSC will be convened biannually either in person or by teleconference. Two PPI representatives will sit on the Trial Steering Committee and will provide input from a patient perspective at trial meetings.

Trial Management Group (TMG)
The Trial Management Group will be set up by the Chief Investigator (CI). TMG will convene on a monthly basis and will discuss on the recruitment, and other practical aspects of the trial. The TMG will include the CI, Project Manager, ICTU representative and PI at the Southampton site as well as other site staff when appropriate. The day-to-day management of the trial will be co-ordinated through the Imperial Clinical Trials Unit via the Project Manager and the Chief Investigator.

Data Monitoring & Ethical Committee (DMEC)
An independent Data Monitoring and Ethical Committee (DMEC) will be set up to monitor progress, patient safety and any ethical issues involved in this trial. They will review trial progress, recruitment rates, event rates and safety data. A separate charter will be drawn up defining their exact remit and criteria for reporting to the trial steering committee. There will be 6-monthly meetings of the independent DMEC.

For peer review only

Background

Recent years have witnessed a global increase in obesity and obesity-related diseases. In 2014, it was estimated that 39% of the world population were overweight (clinically defined as a body mass index (BMI) of 25 – 30 kg/m^2) and 13% were obese (BMI ≥ 30 kg/m^2) and it has been projected that there will be an additional 11 million obese adults in the UK by 2030.[1] Being overweight or obese increases the risk of developing 'metabolic syndrome' and is the main modifiable risk factor for developing insulin resistance and type 2 diabetes mellitus (T2DM). Having a BMI of >25kg/m^2 increases the risk of developing T2DM by 5 times and 90% of adult patients with T2DM are obese or overweight.[2] The prevalence of T2DM has therefore also increased in recent years with an estimated 7.4% of the UK population currently affected and is projected to increase by a further 2.1% in the next 15 years.[3] Compared to the general population patients with T2DM are 87.6% more likely to be admitted to hospital for a myocardial infarction, 121.1% more likely to be admitted for heart failure, 59.1% for a stroke, and are 32% more likely to die prematurely.[4] This represents a significant socioeconomic burden for a largely preventable condition with combined healthcare costs for these conditions estimated to increase by up to 2 billion pounds each year in the UK.[5]

Adipose tissue is a highly active endocrine organ and acts to modulate metabolism by releasing pro-inflammatory cytokines (TNF-α, IL-6, MCP-1),[6] hormones (leptin and adiponectin), glycerol and, importantly, non-esterified fatty acids (NEFA).[7-11] In obesity, especially those with centrally placed adipose tissue, there is increased production of many of these mediators that leads to the development of insulin resistance and pancreatic beta cell dysfunction. T2DM occurs when an already insulin-resistant individual develops beta cell dysfunction and is therefore unable to produce the necessary amount of insulin that is required to maintain normoglycaemia and, as a result, hyperglycaemia predominates.

Dietary modification, exercise, and hypoglycaemic medication remain the mainstay of management for patients with T2DM. Unfortunately, these measures have generally sub-optimal and poorly sustained outcomes. Bariatric, or metabolic, surgery remains the most effective long-term means of treating these patients by producing usually profound and sustained weight loss and weight-loss independent improvements in insulin secretion and sensitivity, consequently ameliorating, or even eliminating, associated co-morbidities and reducing mortality. Roux-en-Y gastric bypass (RYGB) surgery can achieve approximately 23-35% weight loss and 72-90% of patients with T2DM undergoing RYGB are able to achieve sustained euglycaemia without oral hypoglycaemic agents.[12-19] There are several mechanisms by which these outcomes are achieved, namely: (1) gastric exclusion from food by producing a small gastric pouch, (2) exclusion of food from the duodenum and proximal jejunum, (3) early delivery of food to the terminal ileum, and (4) disrupted bile flow. Within the first few days and weeks following surgery, before weight loss has occurred, early improvements in glycaemic control occur through rapid modulation of hepatic insulin resistance (causing reduced hepatic glucose output). This is then followed by sustained long-term weight loss, via entero-neuro-hormonal mechanisms, with an associated reduction in peripheral insulin resistance.[17, 20]

Rubino et al. demonstrated in 2006 that the foregut plays a key role in the metabolic changes that occur following bariatric surgery.[21] They demonstrated that exclusion of the proximal small bowel, as occurs in RYGB and similar procedures such as biliopancreatic diversion (BPD) +/- duodenal switch (DS), results in improved glucose tolerance that occurs independently of effects from reductions in food intake and body weight, malabsorption, or nutrient delivery to the hindgut. These findings have further been substantiated in other studies.[21-24] The proposed mechanisms by which these changes occur include: decreased secretion of orexigenic hormones (ghrelin); increased secretion of glucose-dependent insulinotropic polypeptide (GIP) and cholecystokinin (CCK), increased anorexigenic and incretin hormone secretion (e.g. glucagon-like peptide 1 (GLP-1), peptide YY (PYY), oxyntomodulin), and increased circulating concentrations of plasma bile acids. Additionally, stimulation of vagal afferent nerves in the small bowel cause entero-neuro-endocrine modulation within the gut-liver-brain axis. The resulting net effects include: increased insulin secretion, decreased glucagon secretion, decreased hepatic glucose output, increased pancreatic beta cell mass (via increased proliferation and decreased apoptosis), increased insulin sensitivity, decreased hunger, early satiety and altered food preferences and hedonics and brain reward system responses away from high-energy foods.[20, 25-35]

For peer review only

Such observations have led to the development of novel, endoscopically-placed duodenal-jejunal bypass sleeves (DJBS) or liners. These create an impermeable barrier between chyme exiting the stomach and the intestinal mucosa of the duodenum and proximal jejunum, thus preventing absorption within the foregut. The non-surgical and reversible nature of these devices has sparked much interest in recent years due to the prospect of avoiding the associated surgical mortality and morbidity of bariatric procedures (RYGB: 1 year morbidity 14.9%, 30 day mortality 0.5%).[17] First described by Milone et al in animal models in 2006,[36] the effects of DJBS insertion on reducing weight and potentially improving glycaemic regulation, above that of control interventions, has been validated in five randomised controlled trials (RCTs) and numerous observational studies.[30, 37-49] In a recent systematic review and meta-analysis by Rohde et al.,[50] they concluded that subjects implanted with DJBS achieved an additional 12.6% weight loss compared to sham controls or dietary intervention alone, and a mean greater weight loss of 5.1kg. In the largest of the RCT (DJBS + diet n=38 vs. diet alone n=39) amongst the DJBS arm a significant reduction in HbA1C of -0.9% was found.[37] This finding however was not seen in the meta-analysis by Rohde et al. where the mean difference in HbA1C reduction of 0.8% was non-significant.[50] Finally, evidence exists for DJBS having positive effects on other metabolic parameters, including blood pressure and serum lipid profile.[37, 44, 45]

The EndoBarrier® DJBS (GI Dynamics Inc., Lexington, MA) is delivered endoscopically and comprises of a nitinol metal anchor, which is used to reversibly affix the device to the wall of the duodenal bulb, and an impermeable fluoropolymer sleeve that extends 60cm through the duodenum and into the jejunum (Figure 1). The implant is open at both ends to allow for passage of chyme from the stomach into the lower jejunum and prohibits nutrient absorption along its length by creating a barrier between the partially digested food and the absorptive surface of the small intestine. Whilst the chyme passes through the inside of the EndoBarrier® device, all bile and pancreatic secretions pass on the outside the liner and only mix with the food when they come into contact at the end of the sleeve.

Robust evidence for the clinical use of the DJBS is hence still lacking. The small number of published trials include small participant numbers with high degrees of inter-trial heterogeneity and the results are therefore not generalizable to routine clinical practice. Mechanistic data is also limited. There is therefore a call for more long-term, high quality trial data to validate the efficacy and mechanism of action of this device as a potential tool in the treatment of obesity and metabolic syndrome. In this paper, we describe the methodology for a government funded randomised controlled trial comparing DJBS against best practice medical therapy for the treatment of patients with obesity and T2DM with inadequate glycaemic control.

Methodology

Aims and Objectives

Primary Objective and Endpoint

The primary objective of this study is to evaluate the efficacy of DJBS compared with conventional medical therapy, diet and exercise on glycaemic control. As defined by the International Diabetes Federation (IDF) a substantial improvement in an individual's metabolic state occurs with an improvement in glycosylated haemoglobin (HbA1c) by 20%.[51] Our primary endpoint therefore is a:

i. Reduction in HbA1c by 20% after 12 months of treatment.

Secondary Objectives and Endpoints

The secondary objectives of this study are to evaluate the efficacy, acceptability and cost-effectiveness of DJBS compared against conventional medical therapy, diet and exercise. Secondary endpoints are:

i. HbA1C of < 6%, equivalent to 42 mmol/mol (this infers optimisation of the metabolic state as defined by the IDF)[51]
ii. Blood pressure < 135/85

For peer review only

iii. Weight loss > 15%
iv. Reduction in dose/number of medications
v. Cost of interventions and related health/social care
vi. Quality-adjusted life years (QALY) accrued (calculated from area under the EQ-5D-5L questionnaire curve)
vii. Incremental cost per QALY within the trial period and extrapolated through modelling.

Data will also be obtained to investigate the mechanism of action of the EndoBarrier® device via changes in:

i. Gut hormones
ii. Bile acids
iii. Microbiome
iv. Appetite
v. Food hedonics and preference
vi. Taste
vii. Eating behaviour
viii. Brain reward system responses to food evaluation and addictive behaviours using functional magnetic resonance imaging (MRI)
ix. Body fat content
x. Total body and tissue-specific insulin sensitivity
xi. Intestinal gene expression

Safety Objective

The safety of the EndoBarrier® DJBS will be evaluated during this trial and the type and frequency of adverse events shall be reported.

Research Approval

This study shall be conducted in full conformity with the 1964 Declaration of Helsinki and all subsequent revisions. Local research ethics approval was granted by Fulham Research Ethics Committee, London, (Reference 14/LO/0871) on 10th July 2014. All subjects will give informed written consent.

Study Design

This study is a randomised controlled trial of the EndoBarrier® DJBS compared with conventional medical therapy, diet and exercise for the management of subjects with both obesity and T2DM. Over a two year period (1 year of treatment and 1 year follow-up) the study will be performed over two investigational sites in the United Kingdom: Imperial College Healthcare NHS Trust in London and University Hospital Southampton NHS Foundation Trust. The overall schema for the trial is summarised in Figure 2. To ensure that the study is adequately powered and allowing for drop-outs, n=80 patients will be randomised into each of the two treatment arms equally across the two sites (Table 1).

For peer review only

Table 1. Summary of treatment group

Treatment Group	Number of subjects	Treatment period 1	Follow-up period 2
EndoBarrier Device	80	12 months	12 months
Standard Medical Therapy	80	12 months	12 months
Total number of subjects	**160**		

In order to investigate the mechanism of the effect of the EndoBarrier® device, both treatment arms will be divided into three optional sub-groups, which will have the following additional assessments during the course of the trial:

- Sub-group 1: functional MRI of food reward and addictive behaviours, eating behaviour assessment and post-meal gut hormones.
- Sub-group 2: Euglycaemic, hyperinsulinaemic clamps (total body and tissue-specific insulin resistance).
- Sub-group 3: assessment of taste and food preference, eating behaviour assessment and post-meal gut hormones.

Table 2 summarises the visit schedule, the data to be collected across both study arms and supplementary data that will be collected from the three optional mechanistic sub-groups. In addition to routine follow-up visits, all patients will receive regular telephone counseling from a specialist dietitian to assess their wellbeing and motivation in the trial.

For peer review only

Table 2. Summary of study visit schedule

	Screening	Baseline		Treatment														Follow-up					
**Activities**	**V1**	**V2**	**V3**	**V4**	**T1**	**V5**	**V6**	**T2**	**V7**	**T3**	**V8**	**T4**	**V9**	**T5**	**V10**	**V11**	**T6**	**V12**	**T7**	**V13**	**T8**	**V14**	**V15**
Week/ Month/Day		- 4w ±7d	- 2w ±7d	- 0w ±3d	+ 5d± 3d	+ 10d ±3	+ 1m ±7d	+ 2m ±7d	+ 3m ±7d	+ 4.5m ±7d	+ 6m ±7d	+ 7.5m ±7d	+ 9m ±7d	+ 10.5m ±7d	+ 11.5m ±7d	+ 12m ±7d	+ 13.5m ±7d	+ 15m ±7d	+ 16.5m ±7d	+ 18m ±7d	+ 19.5m ±7d	+ 23m ±7d	+ 24m ±7d
Informed consent (5.1)	X																						
Inclusion & exclusion criteria (4.2, 4.3)	X																						
Demographics (5.1)	X																						
Medical history (including meds) (5.1)	X																						
Physical examination (5.5.4)	X																						
ECG (5.5.5)	X																						
Vital signs (5.5.7)	X	X	X	X		X	X		X		X		X		X	X		X		X		X	X
Body weight (5.5.8)	X	X	X	X		X	X		X		X		X		X	X		X		X		X	X
Height (5.5.8)	X																						
Waist circumference (5.5.9)	X	X	X	X		X	X		X		X		X		X	X		X		X		X	X
Routine blood tests (5.5.10)	X		X			X	X		X		X		X		X	X		X		X		X	X
Urine dipstick and female pregnancy test (5.5.6)	X																						
Changes in medical history/medication (5.5.4)		X	X	X		X	X		X		X		X		X	X		X		X		X	X
Randomisation (5.2)		X																					
Health Economic Questionnaires (5.5.12)			X			X	X		X		X				X							X	
Dietary counselling (5.5.3)		X		C																			
Dietitian follow up (5.5.3)							X		X				X			X		X		X			X
Urine albumin:creatinine ratio (5.5.11)			X			X					X				X							X	
Reporting of AEs (6)		X	X	X		X	X		X		X		X		X	X		X		X		X	X
DNA & RNA sampling (5.5.13)			X			X					X					X							X
Telephone counselling (5.5.3)					X			X		X		X		X			X		X		X		
Diabetologist Review (5.5.1 and 5.5.2)		X		C			X		X				X			C		X		X			X

For peer review only

Activities	Screening	Baseline		Treatment														Follow-up					
	V1	V2	V3	V4	T1	V5	V6	T2	V7	T3	V8	T4	V9	T5	V10	V11	T6	V12	T7	V13	T8	V14	V15
Week/ Month/Day		- 4w ±7d	- 2w ±7d	- 0w ±3d	+ 5d± 3d	+ 10d ±3	+ 1m ±7d	+ 2m ±7d	+ 3m ±7d	+ 4.5m ±7d	+ 6m ±7d	+ 7.5m ±7d	+ 9m ±7d	+ 10.5m ±7d	+ 11.5m ±7d	+ 12m ±7d	+ 13.5m ±7d	+ 15m ±7d	+ 16.5m ±7d	+ 18m ±7d	+ 19.5m ±7d	+ 23m ±7d	+ 24m ±7d
Metabolomics (5.5.14)			X			X					X				X							X	
Bioelectrical Impedance (5.5.15)			X			X					X				X							X	

EndoBarrier Group Only

Activities	V1	V2	V3	V4	T1	V5	V6	T2	V7	T3	V8	T4	V9	T5	V10	V11	T6	V12	T7	V13	T8	V14	V15
PPI and H. Pylori test (5.5.16)		X																					
Distribution of Proton Pump Inhibitors (5.5.2)		T																					
EndoBarrier Implant (5.5.2)				T																			
Preparation for EndoBarrier removal (5.5.2)													T										
EndoBarrier removal (5.5.2)																T							
Biopsies during Implant and Explant (5.5.2)				T												T							
Gastroenterologist appointment (5.5.2)		T					T		T				T			T		T		T*			**T**

Sub-groups

Activities	V1	V2	V3	V4	T1	V5	V6	T2	V7	T3	V8	T4	V9	T5	V10	V11	T6	V12	T7	V13	T8	V14	V15
Fixed/test meal and post-meal gut hormones and metabolites (Group 1 and 3) (5.6.4)			X			X					X				X								
Gut hormones and metabolites (Fasting only) (Group 1-3) (5.6.4)			X			X					X				X							X	
Food diaries (Groups 1-3) (5.6.2)			X			X					X				X							X	
Eating & Behaviour Questionnaires (Groups 1-3) (5.6.1)			X								X				X							X	
Appetite Visual Analogues Scales (Group 1-3) (5.6.3)			X			X					X				X							X	
Eating behaviour computerised tasks (Group 1 and 3) (5.6.5)			X								X				X							X	
Metal Check Form (Group 1) (5.1)	X																						
Handedness Questionnaire (Group 1) (5.1)	X																						

For peer review only

Activities	V1	V2	V3	V4	T1	V5	V6	T2	V7	T3	V8	T4	V9	T5	V10	V11	T6	V12	T7	V13	T8	V14	V15
Additional pregnancy tests			F								F												
	Screening	Baseline		Treatment														Follow-up					
Week/ Month/Day		- 4w ±7d	- 2w ±7d	- 0w ±3d	+ 5d± 3d	+ 10d ±3	+ 1m ±7d	+ 2m ±7d	+ 3m ±7d	+ 4.5m ±7d	+ 6m ±7d	+ 7.5m ±7d	+ 9m ±7d	+ 10.5m ±7d	+ 11.5m ±7d	+ 12m ±7d	+ 13.5m ±7d	+ 15m ±7d	+ 16.5m ±7d	+ 18m ±7d	+ 19.5m ±7d	+ 23m ±7d	+ 24m ±7d
DS-R disgust questionnaire (Group 1) (5.6.6)			X																				
Functional MRI (Group 1) (5.6.9)			X								X												
Insulin Clamps (Groups 2) (5.6.10)			X			X					X												
Cognitive assessment tasks (Group 1) (5.6.9)			X								X				X							X	
Food Preference / Taste Assessment(Group 3) (5.6.11)			X			X					X												
24hr Dietary Recall (Group 3) (5.6.11)			X			X					X				X							X	

X performed in all patients unless otherwise stated

F performed in Females only

C performed in Control arm (Standard medical therapy) only

T performed in Treatment arm (EndoBarrier) only

* optional (at request of the patient)

For peer review only

Study Population

Male and female patients, aged 18–65 years, with a BMI 30–50 kg/m^2 and confirmed diagnosis of T2DM for at least 1 year, who have inadequate glycaemic control and are on oral anti-hyperglycaemic medications. See Table 3 for complete inclusion and exclusion criteria.

Table 3. Study inclusion and exclusion criteria

Inclusion Criteria

1. Age 18–65 years (male or female)
2. Type 2 diabetes mellitus for at least one year
3. HbA1C 7.7–11.0% equivalent to 58 – 97 mmol/mol
4. On oral hypoglycaemic medications
5. BMI 30 – 50 kg/m^2

Exclusion Criteria

1. Language barrier, mental incapacity, unwillingness or inability to understand and be able to complete questionnaires.
2. Non-compliance with eligibility criteria.
3. Females of childbearing potential who are pregnant, breast-feeding or intend to become pregnant or are not using adequate or reliable contraceptive methods.
4. Evidence of absolute insulin deficiency as indicated by clinical assessment, a long duration of T2DM and a fasting plasma C-peptide of <333pmol/L.
5. Current use of insulin.
6. Previous diagnosis with type 1 DM or a history of ketoacidosis.
7. Requirement of NSAIDs (non-steroidal anti-inflammatory drugs) or prescription of anticoagulation therapy during the implant period.
8. Current iron deficiency and/or iron deficiency anaemia.
9. Symptomatic gallstones or kidney stones at the time of screening.
10. History of coagulopathy, upper gastro-intestinal bleeding conditions such as oesophageal or gastric varices, congenital or acquired intestinal telangiectasia.
11. Previous gastrointestinal surgery that could affect the ability to place the device or the function of the implant.
12. History or presence of active H. pylori (if subjects are randomised into the EndoBarrier® arm and have a history or presence of active H. pylori tested at study visit 2 they can receive appropriate treatment and then subsequently enrole into the study).
13. Family history of a known diagnosis or pre-existing symptoms of systemic lupus erythematosus, scleroderma or other autoimmune connective tissue disorder.
14. Severe liver impairment (i.e. AST, ALT or gGT >4 times upper limit of the reference range) or kidney impairment (i.e. estimated Glomerular Filtration Rate (GFR) < 45 ml/min/1.73m^2.
15. Severe depression, unstable emotional or psychological characteristics (including Beck Depression Inventory II score >28).
16. Poor dentition and inability to adequately chew food.
17. Planned holidays up to three months following the EndoBarrier Implant.

For peer review only

Study Recruitment

Participants will be identified from several areas across primary, secondary and tertiary healthcare and community settings:

i. Diabetes research registers (e.g. Diabetes Alliance for Research in England (DARE), REC 2002/7/118)
ii. Hospital or General Practice (GP) patient databases (Participant Identification Centres)
iii. Patients referred to diabetes and bariatric specialist clinics
iv. Other research studies within the Imperial College Healthcare NHS Trust and the Local Clinical Research Network (LCRN)
v. Study websites
vi. Local and national media – websites, radio, newspaper articles and adverts

vii. Posters
viii. Diabetes, Obesity and other support groups
ix. Social media websites

Potential patients who, after reading a summary Patient Information Sheet (PIS), would like to enter the trial will give their verbal consent for preliminary telephone screening to check basic inclusion and exclusion criteria. Written consent will then be taken from the patient to allow the study team to contact their GP for the purpose of obtaining additional information on the patient's medical history, current medical therapies and to identify any other clinical reasons as to why the patient should not participate. Patients who appear to meet eligibility criteria will be provided with a full trial PIS and then invited to a formal screening visit at one of the study centres. At this stage the patient will be fully informed of the nature of the study and given relevant information about the objectives of the research, benefits and possible adverse events, verbally and in writing. The patient will have the opportunity to ask questions about the trial and formal written consent will be taken for the patient to participate in the main study ± additional consent for one of the three optional mechanistic sub-groups. Once consent has been obtained then the subject's full eligibility will be checked against all inclusion and exclusion criteria (Table 3). Each patient will be informed of their eligibility for the trial once all results are available (usually within one week from obtaining consent).

Randomisation

Eligible patients will be randomised into one of the two trial arms using the InForm Integrated Trial Management system, a secure web-based data entry platform. This will be programmed with a randomisation schedule by an independent statistician and protect against bias in the randomisation process as group allocation will be concealed and automatic. The randomisation will be at a ratio of 1:1 and stratified by site and two BMI groups, 30–40 and 40–50 kg/m^2. Each patient will be informed of their randomisation allocation and will be assigned a unique study identification number. Only the subject number and initials will be recorded in the case report form (CRF). All other patient-identifiable data will be completely anonymised.

Trial Interventions

EndoBarrier® Gastrointestinal Liner

The EndoBarrier® Gastrointestinal liner device received CE Mark for 12 months implant duration on 11th December 2009 and is a single use, minimally invasive device, used to achieve weight loss and improve T2DM status in subjects who are obese (Figure 1).

At visit 2 (- 4 weeks), participants who have been randomised to receive the EndoBarrier® device will be tested for the presence of Helicobacter pylori, either by faecal antigen or urea breath testing. Those patients testing positive will be offered 1 week of triple-eradication therapy, as per guidance published within the British National Formulary, and will then be retested after a further 4 weeks to confirm complete eradication before continuing with implantation of the EndoBarrier® device. Subsequently, all patients will be prescribed a proton pump inhibtor (PPI) (Omeprazole 40mg twice daily) and instructed to commence this three days prior to the implant procedure. They will continue this for the duration of the implant period (12 months) and for a further two weeks following device removal.

At visit 4 (0 weeks), after an eight-hour fast, subjects will have the EndoBarrier® device implanted under a general anaesthetic. The implant is delivered endoscopically on a custom catheter and the anchor is sited in the duodenal bulb using a custom delivery system under fluoroscopic x-ray guidance (mean fluoroscopic x-ray time for insertion is 7 minutes, range 1-20 minutes). The 60cm sleeve is unfurled and then the final positioning plus patency is confirmed by assessing for the free flow of radio-opaque contrast through the device. Videos and photos of the fluoroscopy images are recorded to help the investigators make treatment decisions. During implantation eight gastric and small bowel biopsies will be taken using standard biopsy forceps. Four biopsies will be used for routine histology and four biopsies will be used for RNA extraction to perform genome-wide expression analysis. Participants will be discharged from hospital the same day with an implant information card, which describes the implant, identifies who to call in

For peer review only

the case of an emergency, and what symptoms to look for following the implant. Subjects will have their dose of sulphonylurea medication reduced by 50% at the time of EndoBarrier® implant to avoid potential hypoglycaemic episodes.

The device will be removed at visit 11 (after 12 months) under sedation or general anaesthetic. The gastroscope, which is fitted with a foreign body retrieval hood, is used to locate the implant and a custom grasper is passed through the working channel of the gastroscope to grab a polypropylene tether located on the proximal portion of the anchor. Pulling on this tether will collapse the proximal end of the anchor, which can then be pulled into the foreign body hood and removed by withdrawing the gastroscope through the subject's mouth. During this removal, eight further biopsies will be taken for histology and RNA extraction. Following removal of the EndoBarrier® device, patients will be followed up for a further 12 months.

Diabetes Review

Participants in both arms of the trial will have their T2DM managed in accordance with the guidelines of the American Diabetes Association (ADA).[52, 53] These guidelines have been chosen as they would adhere to the current best worldwide practice that would still be relevant when the results are published following study completion. Both treatment groups will have a review of their T2DM by a suitably trained physician at visits 2, 6, 7, 9, 12, 13 and 15. Additionally, the standard care arm of the trial will have an additional review at visits 4 and 11 in place of the EndoBarrier® implant and removal. Adjustments to a patient's oral anti-hyperglycaemic medication and escalation of therapy is at the investigators discretion and will comply with general recommendations laid out by the ADA.[53]

Dietary Counselling and Physical Activity

At visit 2, all patients historical and current eating behaviours will be assessed by a qualified dietitian using the following information: anthropometry; biochemistry; co-morbidities; activity levels; eating habits including previous diets; lifestyle including smoking, drug and alcohol misuse; weight history; psychiatric history; family history of obesity, diabetes, mental illness or eating disorders; available support network; work status; readiness and motivation for change. Patients will then receive dietary and physical activity counselling in accordance with local standards with the intention of providing each subject with lifestyle/behavioural modification information and good eating practices. In addition, subjects in the EndoBarrier® arm will receive written information on how their diet will change after implantation of the device and they will receive specialist guidance for eating with their EndoBarrier®.

All patients will be reviewed by a specialist dietitian at visits 2, 6, 7, 9, 12, 13 and 15. In addition, participants in the standard care arm of the trial will have an additional review at visits 4 and 11 in place of the EndoBarrier® implant and removal. During the course of the trial, participants will be recommended to consume 600 kcal less every day, depending on their age, gender, activity levels and body weight. Guidelines for daily amounts are between 1200 and 1500 kcal for women and between 1500 and 1800 kcal for men. In accordance with standard dietary practice, subjects will be advised: to eat regularly every day (5 times per day); to control their portion sizes and intake of carbohydrates/starchy foods; to increase their intake of low glycaemic index (GI) and high protein foods, as well as vegetables; and to reduce their intake of foods high in fat and sugar, and alcohol. Participants will be advised to include more physical activity in their daily routine and encouraged to do more activity in their leisure time. Their goal will be to include 150 minutes a week of moderate intensity, and 75 minutes a week of vigorous intensity aerobic activity and muscle strengthening activities, on more than 2 days a week. Changes in physical activity level will be monitored using the International Physical Activity Questionnaire (IPAQ).[54]

Liquid Diet

To avoid disruption of the device in the immediate period following implantation, patients will follow a liquid diet for the 7 days before and 13 days (± 3 days) after the intervention visit (visit 4). The liquid diet will be guided by the

For peer review only

specialist dietitian and will comprise of 125ml Fortisip Compact drinks (Nutricia, UK): 5 per day for males, 4 per day for females, containing per 100 mL: 240 kcal, 9.6g protein (16% total energy), 29.7 g carbohydrate (49%), 15g sugars, 9.3g fat (35%). Patients will also be allowed to consume sugar-free squashes, smooth/clear soup (1 medium bowl per day), tea or coffee without sugar, or unsweetened puree. To standardise both therapy groups, all patients across both arms will follow the liquid diet for this duration and period of the study.

Assessment of Objectives

Assessment of Primary Objective

Each study participant will have their IFCC (International Federation of Clinical Chemistry) HbA1C measured at screening and then subsequently at visits 5, 7, 8, 9, 10, 12, 13 and 15. Samples will be processed at the laboratory local to each study centre using standard methods. Results will be recorded on the InForm system.

Assessment of Secondary Objectives

Individuals in both study arms will be invited for regular medical check-ups (Figure 2), which will include routine anthropometric measurements (height, weight, waist circumference, pulse and blood pressure) and blood tests (Table 4). Any changes to the participants' health or medications will be carefully documented on the CRF and all adverse events will be reported in detail in line with standard principles of GCP.

Table 4. Summary of blood tests at each study visit

Blood test	V1	V3	V5	V6	V7	V8	V9	V10	V11	V12	V13	V14	V15
Haematology (Full blood count)	x	x	x	x	x	x	x	x	x	x	x	x	x
Routine Biochemistry (including Urea and Electrolytes)	x	x	x	x	x	x	x	x	x	x	x	x	x
Liver function tests	x	x	x	x	x	x	x	x	x	x	x	x	x
Fasting glucose	x	x	x	x	x	x	x	x	x	x	x	x	x
Creatinine	x	x	x	x	x	x	x	x	x	x	x	x	x
HbA1C	x		x		x	x	x	x		x	x		x
Fasting lipids (cholesterol, HDL, LDL, triglycerides)	x	x	x	x	x	x	x	x		x	x	x	x
C-peptide	x												
Insulin (Fasting)	x	x	x			x		x				x	
Vitamin D	x					x		x					
Iron studies	x					x		x					
Vitamin B12	x					x		x					
Serum Folate	x					x		x					
Free Thyroxine	x							x					x

For peer review only

TSH	x							x					x
Cortisol (Sub-group 1 only)		x				x							
Oestradiol (Sub-group 1 only)		x				x							
Progesterone (Sub-group 1 only)		x				x							
LH (Sub-group 1 only)		x				x							
FSH (Sub-group 1 only)		x				x							

Patients in both treatment arms will be asked to complete health economics questionnaires at visits 3, 5, 6, 7, 8, 10 and 14. These comprise of the EQ-5D-5L questionnaire to assess health-related quality of life and a bespoke questionnaire designed to collect information about patients' use of health and social care resources (for costing purposes).[55] The Resource Use questionnaire will be adapted from existing instruments and will include:[56, 57]

i. Medications for diabetes, weight loss, blood pressure, lipid control and cardiovascular disease
ii. Primary care consultations (with GP, nurse or other healthcare professional)
iii. Hospital outpatient clinic visits (by specialty)
iv. A&E attendances (admitted/not-admitted)
v. Inpatient stays and procedures
vi. Investigations
vii. Use of any other NHS-related community health and social services (e.g. chiropody).

Costs for private health and social care, out-of-pocket expenditure by patients, and 'indirect costs' per patient time will not be included.

In addition, data will be collected in the CRF from hospital information systems and case notes to cost the EndoBarrier® intervention and the diet/exercise intervention. Information to be collected will include:

i. Routine assessments required before implantation and removal of the EndoBarrier®
ii. Disposables and staff time for the insertion and removal procedures, including day case and overnight stay if required
iii. Treatment of any adverse events related to the procedures (additional inpatient stays, clinic visits and re-admissions)
iv. Dietitian time to deliver the diet and physical activity counselling and for telephone follow-up
v. Routine hospital follow-up and diabetes care
vi. Hospital treatment for cardiovascular events or other complications of diabetes

Mechanistic Study Groups

In addition to the routine data collected above, at visits 3, 5, 8, 10, and 14, mechanistic data will be gathered from all patients across both study arms:

i. Body fat mass (kg and % of body weight) measured by bio-electrical impedance analysis
ii. Collection of stool, urine and plasma for assessment of metabonomics using nuclear magnetic resonance (NMR) spectroscopy and mass spectrometry (MS), and microbiome analysis
iii. Measurement of insulin, gut hormones (ghrelin, GLP-1, PYY), bile acids, leptin and other adipocytokines, and markers of insulin resistance and inflammation from venous blood samples (fasting for all visits and then

For peer review only

following a meal in sub-groups 1 and 3 at visits 3, 5, 8 and 10)

iv. DNA and RNA from venous blood samples for examination of genetic variants that may predict weight loss, cause or contribute to obesity

v. Urinary albumin:creatinine ratio

vi. Comprehensive 3-day food diary

Sub-group 1: Functional MRI

Subjects in each study arm, at the London site only, will have fMRI scans to examine brain function related to food reward and addictive behaviours contributing to overeating at baseline (visit 3) and at 6 months (visit 8) after intervention. These will be supplemented by and correlated with psychological questionnaires, computerised tasks and test meals at these and other visits. The study visits will last up to 6-8 hours (scanning visits 3 and 8). Subjects will have structural and functional MRI brain scans lasting up to 90 minutes using a 3.0 Tesla Siemens Verio MR scanner after an overnight fast. Whilst in the scanner, subjects view a mirror reflecting a computer screen and can respond to instructions using a keypad held in their hand.

The following anatomical brain scans will be collected at both visits:

i. Anatomical T1 and T2-weighted MR scans to provide structural neuroimaging data and allow image registration to standard space.

ii. Diffusion tensor imaging to examine white matter tract integrity.

The following resting state functional MRI scans will be collected at both visits:

i. Arterial spin labelling to measure resting regional cerebral blood flow.[58, 59]

ii. Resting state blood oxygen level-dependent (BOLD) functional MRI to measure resting state functional connectivity.[60]

The following task-related functional MRI scans will be collected at both visits:

i. Food picture evaluation task: to assess reward system activation when subjects view a variety of different pictures (high-energy and low-energy foods, household objects, blurred pictures as a baseline) and simultaneously rate how 'appealing' the pictures are using the keypad as a measure of anticipatory food reward or food due reactivity.[32, 34, 61-63]

ii. Monetary incentive delay (MID) task: a game in which subjects need to press a button during a specific time window when given a cue on the computer screen in order to win or prevent the loss of hypothetical monetary prizes to assess anticipatory non-food reward responsivity.[64-66]

iii. Go-NoGo task: to assess motor response inhibitory control as a measure of compulsivity.[67-69] The task contrasts brain activation during responses to infrequent no-go signals (e.g. ‘do not press’ button when viewing one symbol) compared to an implicit go baseline (e.g. ‘do press’ button when viewing a different symbol).[66]

iv. Negative emotional reactivity task: to assess brain response during viewing of unpleasant, negatively valent pictures compared to neutral control pictures.[66]

Study visits without scanning at visits 5, 10 and 14 will last 3-6 hours. At visits 3, 8, 10 and/or 14, subjects in sub-group 1 will also complete several questionnaires and perform several computer-based tasks to assess eating and addictive behaviours and cognition, including:

i. WTAR word reading list: to document baseline intellectual status (visit 3 only)

ii. Kirby delay discounting task: to assess temporal impulsivity to a hypothetical non-food monetary rewards (visits 3, 8, 10 and 14).[70]

iii. Leeds Food Preference Questionnaire: to assess bias of food preference to foods high in fat and sugar and explicit and implicit liking of foods high/low in fat/sugar (visits 3, 8, 10 and 14).[71]

iv. Progressive ratio task: to measure breakpoint of effort that subjects are willing to spend by having to press a computer mouse an increasing number of times to receive a chocolate M&M™ sweet, to assess appetitive food reward and motivation (visits 3 and 8).[33, 34]

An *ad libitum* test meal will be performed at visits 3 and 8, in which subjects first taste and rate the palatability,

For peer review only

intensity and acceptability of different foods high or low in fat and sugar, and then eat as much of whichever foods they want, to assess food taste, preference and choice, total energy intake and macronutrient composition. Subjects will also complete visual analogue ratings of appetite, anxiety, stress and sleepiness, and measurement of fasting and post-meal hormones and metabolites over the study visit.[32, 63]

At visit 5, 10 and 14 subjects will consume a fixed mixed meal tolerance test with measurement of fasting and post-prandial hormones and metabolites.

Sub-group 2: Euglycaemic, hyperinsulinaemic clamp

On visit 3, 5, and 8, patients in each study arm, at the Southampton site only, will undergo a euglycaemic hyperinsulinaemic clamp with stable isotope infusion to determine overall insulin and compartment-specific insulin sensitivity (liver, muscle and adipose depot). Patients will be instructed to consume a standardised meal or meal replacement the evening prior to their study visit. A venous catheter will be inserted into a vein of each arm on the study morning. The first cannula will be used for infusions and the other for blood sampling. If blood glucose levels are higher than 6 mmol/L on arrival then a variable rate insulin infusion will be started to attain a stable glucose level (4.0 -6.0 mmol/L) prior to commencement of the hyperinsulinaemic euglycaemic clamp.

A primed continuous infusion of 6, 6-$^2H_2$-glucose, a stable isotope tracer, will be started and maintained for 7 hours. Two hours later a two-stage hyperinsulinaemic-euglycaemic clamp procedure will be started and continued for 5 hours. During stage 1 of the clamp procedure, in which hepatic insulin resistance is assessed, insulin will be infused at a low dose (0.3-0.5 mU/kg/min) for 2 hours. During stage 2 of the clamp procedure, in which peripheral insulin resistance is assessed, insulin will be increased to a higher dose (1.5 mU/kg/min) for 2 hours. Euglycaemia will be maintained by infusing 20% dextrose at a variable rate. Blood samples will be taken every 5 minutes to measure blood glucose concentration and the dextrose infusion will be adjusted accordingly. The exogenous glucose infusion will be enriched with 6, 6-$^2H_2$-glucose to prevent a fall in plasma tracer enrichment and underestimation of endogenous glucose production rate. Blood samples will be obtained before the start of the tracer infusions, every 10 min during the final 30 min of the basal period and stages 1 and 2 of the clamp procedure and every 30 minutes between these periods to determine glucose enrichment and concentration, free fatty acid, insulin, C-peptide, glucagon, gut hormones, and metabolite concentrations. At the same time points participants will be asked to complete appetite visual analogue scales.

The isotopic enrichment of plasma glucose will be determined by gas chromatography mass spectrometry (GCMS) at the Wolfson Centre for Translational Research, Postgraduate Medical School, University of Surrey, United Kingdom.

Sub-group 3: Taste and Food Preference Assessment

On visit 3, 5, 8, 10 and 14, patients in each study arm, at both the London and Southampton sites, will attend the research facility after an overnight fast. The total duration of these visits will be up to 7 hours (visits 3, 5, 8, 10) and 5 hours (visit 14). On the morning of those visits patients will perform two behavioural computerized tasks: the Kirby Delay Discounting Task to assess temporal impulsivity to non-food monetary reward (visits 3, 8, 10 and 14);[70] and the Leeds Food Preference Questionnaire to assess bias of food preference to foods high in fat and sugar and explicit and implicit liking of foods high/low in fat/sugar (visits 3, 8, 10 and 14).[71]

Sweet taste detection testing will be performed at visits 3, 5, 8 by following the method of constant stimuli in which seven ascending sucrose concentrations in solution will be used to determine sweet detection thresholds.[72] At the same visits consummatory taste reward will be assessed in which five ascending sucrose solutions will be used to test responses in intensity ratings and hedonic reward. To assess the appetitive behaviour towards a sweet and fatty food reward, a Progressive Ratio Task (PRT) will be performed 2 hours after the consumption of a fixed meal.[33]

Total caloric intake and macronutrient composition will be assessed using three-day food diaries and a 24-hour recall that is carried out by a trained dietitian/nutritionist on all visits. Patients will also complete the EPIC Food Frequency questionnaire at visit 3, 8, and 10. Finally, a fixed mixed meal tolerance test with measurement of post-meal hormones and metabolites will be performed.

For peer review only

Additional sub-group specific procedures and measurements

Across all three sub-groups only, the following additional data will also be collected during the mechanistic study visits:

i. Trait, state and symptom questionnaires: to evaluate aspects of eating behaviour (e.g. dietary restraint, emotional eating, disinhibition, hunger, external eating), reward sensitivity, mood, impulsivity, aversive symptoms, symptoms of dumping syndrome, including Dutch Eating Behaviour Questionnaire, Three Factor Eating Questionnaire, Yale Food Addiction Scale, Binge Eating Scale, Beck Depression Inventory II, Hospital Anxiety and Depression Scale (HADS), Barratt Impulsiveness Scale (BIS), UPPS-P Impulsive Behavior Scale.
ii. Visual analogue scale (VAS) ratings: to assess subjective feelings of hunger, nausea, fullness, sleepiness, stress and anxiety when fasted and during meal tests.

Power Calculation

Conservatively, it was estimated that 15% of patients in the control arm will achieve the target but we believe this to be an overestimate. The Steno study is the best quality randomised study (n=80 patients in each arm) into the effect of best medical therapy published to date and demonstrated over an average 7.8 years significant improvements in HbA1c amongst those having intensive medical therapy from 8.4 ± 1.6 to 7.7 ± 1.2, but no change in HbA1c amongst those continuing with standard medical therapy.[73] This study defines the very best that could realistically be achieved in the control arm, but expect there to be very little if any change in this group. The reporting of HbA1c as an outcome measure was not in accordance with the newly defined IDF criteria, but considering the small average reduction achieved in the Steno study, it will be assumed that a target of 15% of patients reaching the endpoint is a conservative estimate. Company data on the small number of patients who have reached a year with the device in place suggest that 40% will achieve this target.

According to our own experience with the device in a commercially sponsored study, up to 30% of patients in the treatment group may have the device removed early. Nevertheless other commercially sponsored (unpublished) studies of this device have achieved lower explant rates (J Tetreault – GI Dynamics). To allow for up to 30% early removal we have therefore diluted the treatment effect from 40% vs. 15% to 35% vs. 15% achieving the target of 20% reduction in HbA1c for treatment arm vs. standard arm. With these assumptions, n=73 patients per group will give 80% power with a two-sided alpha 0.05 to detect a significant effect. Adding 10% loss of follow-up increases the sample size to n=80 per group.

The dilution was calculated starting from the assumption that 40% of patients with the device will reach the target (this estimate is based on company data based on diabetic patients in the same range of BMI as in the present proposal). If 30% of patients in the treatment group need to remove the device early but remain available for follow-up, in the worst case scenario, the proportion reaching the target is the same as in the control group, bringing the estimate for the treatment group to 32.5%. However most of them will keep the device for some time, having some benefit, so it is plausible to assume that the estimate is higher than 32.5%. Dividing the main effect 15% vs. 40% in three parts we assume that in the 30% of patients with removal, for 1/3 the same effect will be achieved as in the control group (15% reaching the target), for 1/3 it will be increased (23% reach the target) and for 1/3 more increased (31% reach the target). Overall, this would give an estimate of 35% for the treatment group.

Statistical Analysis

Patient characteristics will be summarized. Summaries of continuous variables will be presented as means and standard deviations if normally distributed, and as medians and inter-quartile ranges for skewed data, whilst categorical variables will be presented as frequencies and percentages. The difference between the two study groups in the proportion of patients achieving substantial improvement in the metabolic syndrome both at 12 and 18 months will be analysed using logistic regression adjusting for the stratification variables (BMI groups and sites). Analysis of secondary outcomes will be conducted using standard statistical procedures applicable to categorical or continuous data as appropriate. For missing values we will explore the pattern and the extent of missingness and we

For peer review only

will carry out an appropriate form of multiple imputation if required. The analysis will be performed according to the intention to treat principle. All statistical tests will be two-tailed with a 5% significance level.

Metabonomics and Microbiome Analyses

Metabonomic datasets will be analysed using principal component analysis (PCA) and orthogonal partial least-squares analysis (O-PLS). The metabolic and microbial data will also be analysed in relation to response measurements such as BMI, gut hormone levels etc. using O-PLS regression analysis and Bayesian approaches. A range of statistical methods will be optimised and applied to the data to identify weight loss and T2DM-associated microbiota and metabolites.

Health Economics

The economic health analysis will be conducted following the NICE Reference Case, which includes the use of QALYs as the measure of health outcome, and adoption of an NHS and Personal Social Services perspective for costs (Guide to the methods of technology appraisal 2013, http://publications.nice.org.uk/pmg9).

Within trial analysis

EQ-5D-5L health states will be scored using the English value set to give a utility values at each time point (-2 weeks, 10 days, 1, 3, 6, 11.5, and 23 months).[74] QALYs will be estimated for each patient using an area-under-the curve approach.

The cost of the EndoBarrier® intervention, the lifestyle intervention, and other related health and social care will be estimated from resource use data. Unit costs for the included services will be obtained from standard national sources (BNF or Drug Tariff for drug prices, Department of Health Reference Costs for investigations, procedures and outpatient visits, PSSRU estimates for other primary and community health and social services). Total costs will be estimated for each patient over the 24-month trial period.

Patient-level cost and QALY estimates will be combined to estimate an Incremental Cost Effectiveness Ratio (ICER) for the EndoBarrier® device compared with standard medical therapy over the 24 month trial period. The analysis will combine multiple imputation to account for missing EQ-5D and resource use data with bootstrap regression to estimate mean cost and QALYs for the two patient groups. Missing data is often a particular problem for economic analysis, even in studies with good follow-up of primary endpoints, as area under the curve approach requires data from multiple time points. A bootstrap regression approach will be used to account for non-normal distributions of cost data, to adjust for baseline differences in utility or other patient characteristics, and to allow for correlations between costs and QALYs.

Cost-effectiveness modelling

A decision analytic model will be developed to estimate clinical outcomes, QALYs, and costs beyond 24 months. The time horizon for the modelling will be for the remainder of the patient's predicted lifetimes, as recommended in modelling guidelines.[75, 76] Before commencing this modelling exercise, a review of published economic decision models for weight loss interventions for people with T2DM will be conducted in order to identify possible model structures and sources of input parameters. The conceptual design of the model will be discussed and agreed amongst the research team before programming commences. It is anticipated that the model will take the form of an individual patient simulation, using either a discrete time or discrete event approach to simulate the onset of diabetes/obesity related complications, and hence QALYs and costs under alternative treatment strategies.

Data from the trial will be used to provide estimates of the effect of the EndoBarrier® compared with conventional management. In addition, published systematic reviews and meta-analyses of bariatric surgery for similar a patient population will be reviewed. If recent evidence of sufficient relevance and quality is available, we will extend our model to include indirect comparisons with these other interventions. Other model parameters will be sourced from targeted literature reviews and routine data sources. The choice of software for the model will be made after specification of the conceptual design. Before use, the model will be validated by an experienced health economist not involved in the development of the model. This will be done using a checklist developed by the Brunel Health

For peer review only

Economics Research Group, which includes a range of suggestions for checking that a model is free from errors (verification) and that it is consistent with internal and external data (validation).

Probabilistic sensitivity analysis (PSA) will be used to estimate the impact of uncertainty over model parameters, and value of information (VOI) analysis to estimate the value of conducting further research. In addition, deterministic sensitivity analysis will be used to examine the impact of uncertainties over the model structure.

Gut hormones, metabolites and bile acids

These will be measured in the fasted and/or postprandial state for each patient and compared within and between the groups using parametric/non-parametric repeated measures statistical testing.

Food hedonics and brain reward responses

Brain activation during fMRI paradigms and outcomes from behavioural measures of eating and addictive behaviours and questionnaires, will be compared between groups using a 2x2 ANOVA design including group (control vs. EndoBarrier®) as a between subject factor, time (baseline vs. follow-up visit) as a within subject factor, and group x time interaction to identify differential effects between groups. For fMRI studies, analysis will use region of interest (e.g. for food picture evaluation task: orbitofrontal cortex (OFC), amygdala, caudate, nucleus accumbens, anterior insula) and whole brain analyses to compare groups using statistical thresholds of voxel-wise correction false discovery rate (FDR) $P<0.05$ or cluster-wise family wise error (FWE) correction $P<0.05$. Correlations of BOLD signal will be made with other behavioural variables by linear regression analysis to examine the relevance of changes in brain activation.

Food Preference and Sweet Taste

Dietary energy intake, macronutrient composition, sweet taste detection thresholds, and visual analogue taste ratings will be quantified for each patient and compared within and between the groups at different time points using parametric/non-parametric repeated measures statistical testing. Regressions will be performed with clinical outcomes (e.g. weight loss, HbA1c) to identify predictive markers and generate mechanistic hypotheses.

Hyperinsulinaemic Euglycaemic Clamps

Overall and tissue specific insulin sensitivity will be quantified for each patient and compared within and between the groups at 3 time points using parametric/non-parametric repeated measures statistical testing.

In addition, linear regression will be performed to correlate mechanistic variables collected from each of the sub-groups 1-3 at baseline or during the intervention with clinical outcomes at 1 year e.g. weight loss and decreases in HbA1c, to generate predictive markers and generate mechanistic hypotheses.

Discussion

Experience of 3717 EndoBarrier® devices distributed worldwide has demonstrated a favourable risk-to-benefit ratio (GI Dynamics, February 2017) and their minimally invasive and reversible nature represents a very attractive treatment modality for patients with obesity and T2DM. Evidence already exists in the literature in support of the efficacy of DJBS by reducing weight and potentially improving glycaemic control.[37, 38, 40, 42, 44-46, 50] Nonetheless, it is reported that up to 100% of patients will experience a non-serious adverse event (predominantly abdominal discomfort and nausea immediately following implantation)[50] and 7.4% will suffer a serious adverse event (SAE) (GI Dynamics safety reporting 2008 to March 2017). The exact nature of these events are summarised in Table 5.

For peer review only

Table 5. Serious Adverse Events from the EndoBarrier® device

	2008	2009	2010	2011	2012	2013	2014	2015	2016	2017 (Jan – March)	Total
**Distributed devices**	**25**	**143**	**157**	**275**	**391**	**812**	**987**	**482**	**383**	**43**	
Hepatic Abscess	0	0	0	0	1	8	12	9	4	2	36
Hepatic Abscess Rate											1.0%
Hepatic Abscess with explant ≤ 12 months	0	0	0	0	1	5	9	7	1	2	25
Hepatic Abscess Rate											0.7%
Intolerance	0	5	4	12	5	10	11	4	18	0	69
Intolerance Rate											1.9%
Liner Obstruction	0	4	0	5	3	1	10	2	0	0	25
Liner Obstruction Rate											0.7%
GI Bleed	0	1	3	6	5	9	20	8	4	1	57
GI Bleed Rate											1.5%
Migration/Movement	0	10	6	4	1	6	11	13	0	1	52
Migration/Movement Rate											1.4%
Pancreatitis	0	0	0	0	4	1	7	0	0	0	12
Pancreatitis Rate											0.3%
Perforation	0	0	1	0	0	5	2	2	1	0	11
Perforation Rate											0.3%
Surgical Removal	0	0	0	1	1	0	8	1	1	0	12
Surgical Removal Rate											0.3%
**Total Incidences**	**0**	**20**	**14**	**28**	**20**	**40**	**81**	**39**	**28**	**4**	**274**
**Total Cumulative Rate**											**7.4%**

GI dynamics safety reporting 2008 to March 2017

For peer review only

Notably, the pivotal U.S ENDO trial (EndoBarrier® vs. Sham procedure) was terminated in July 2015 after only 325 subjects were randomised (n=216 EndoBarrier subjects) due to a higher than expected hepatic abscess (HA) rate of 3.5% (compared to a global incidence of 0.73%). This high incidence of HA is not the experience within Europe with 1.2% being reported in 1901 distributed devices (UK hepatic abscess rate in 523 cases is 1.34%). There have also been no deaths attributed to the EndoBarrier® and all patients experiencing an SAE have recovered without long-term sequelae.

Research to date therefore validates the EndoBarrier® DJBS as a potential treatment option for patients who are obese with or without T2DM. These studies however have been limited by their low participant numbers, short follow-up duration and wide inter-trial heterogeneity. Thus, there is a call for more robust clinical trial data to investigate its efficacy, safety and acceptability, and to establish where its use may fall within the treatment algorithm of such patients. This study will represent the largest randomised controlled trial of the EndoBarrier® device compared with conventional medical therapy, diet and exercise over a treatment period of 1 year and will also provide the longest follow-up data (1 year) of any trial to date. Additionally, this study will provide: (1) unique data on the mechanism of action of the DJBS and the effect of foregut exclusion on an individual's metabolic profile, (2) a cost-effectiveness analysis, (3) quality of life assessment outcomes, and (4) extensive safety data.

As this study is an open trial, in which the participants, clinicians and hospital staff will not be blinded to their treatment allocation, it is at risk of bias, particularly performance or observer bias. A control group undergoing a sham endoscopy would significantly reduce this bias but would expose a large number of patients to the risks of an unnecessary endoscopic procedure and general anaesthetic. Therefore, to reduce the effect of bias: (1) participants will undergo a concealed computer-generated randomisation process by an independent statistician, (2) multiple assessors across both study sites will follow structured assessment protocols and utilise validated measurement tools in order to minimise subjectivity from the data collection, (3) data collection will be monitored regularly to ensure adherence to the protocol and to perform source data verification, and (4), where possible, outcomes and results will be reported by an independent person who is unaware of the treatment allocation of the participant (e.g. the primary outcome measure of HbA1c and all other haematological or biochemical samples will be measured and reported by an independent laboratory technician at each hospital). Attrition bias will be minimised by performing regular scheduled follow-up visits across both treatment groups and regular telephone follow-ups will be performed in order to assess the patient's wellbeing and motivation on the trial. Patients selected for this trial will be a very motivated subset of the population of interest. The effects of this sampling bias will be minimized through effective randomization but will reduce the generalisability of any significant treatment effect identified.

To conclude, we hypothesize that exclusion of the foregut by means of an EndoBarrier® device will improve glycaemic control, above that of conventional medical therapy, diet and exercise via: (i) decreased hepatic insulin resistance and increased insulin production, that occurs independent of weight loss and caloric restriction, and (ii) through reduction in total body and tissue-specific insulin resistance as a result of consequent weight loss. We also hypothesize that this device will produce weight loss, above that of control patients, by reducing hunger, increasing satiety (therefore reducing food intake) and changing food preferences and hedonics away from high-energy sweet and fatty foods. If the EndoBarrier® is effective at achieving long-lasting weight loss and glycaemic control, there is an obvious potential for health benefit and savings on future health and social care; through the avoidance of T2DM and related complications.

Trial Status

The trial opened for recruitment at Imperial College Healthcare NHS Trust in London on 18th November 2014 and then in University Hospital Southampton NHS Foundation Trust on 5th June 2015. Recruitment was completed across both sites on 18th October 2016 and all EndoBarrier® devices were inserted by 23rd January 2017. Participant follow-up continues across both sites with the anticipated trial completion date being 23rd January 2019.

For peer review only

References

1. World Health Organisation. *Obesity and overweight Fact sheet*: World Health Organisation; 2016. Available from: http://www.who.int/mediacentre/factsheets/fs311/en/. Accessed 2nd February 2017
2. Public Health England. *Adult obesity and type 2 diabetes.* PHE publications gateway number: 2014211. London: Public Health England; 2014.
3. The National Cardiovascular Intelligence Network. *Cardiovascular disease key facts. Fact sheet number 8.* PHE publications gateway number: 2013_237. London: Public Health England; 2013.
4. Health and Social Care Information Centre. *National Diabetes Audit 2012-2013, Report 2: Complications and Mortality.* Leeds: Health and Social Care Information Centre; 2015.
5. Wang YC, McPherson K, Marsh T, et al. Health and economic burden of the projected obesity trends in the USA and the UK. *The Lancet*. 2011;378(9793):815-25.
6. Wellen KE, Hotamisligil GS. Inflammation, stress, and diabetes. *J Clin Invest*. 2005;115(5):1111-9.
7. Kershaw EE, Flier JS. Adipose tissue as an endocrine organ. *J Clin Endocrinol Metab*. 2004;89(6):2548-56.
8. Kahn SE, Hull RL, Utzschneider KM. Mechanisms linking obesity to insulin resistance and type 2 diabetes. *Nature*. 2006;444(7121):840-6.
9. Boden G. Obesity, Insulin Resistance and Free Fatty Acids. *Curr Opin Endocrinol Diabetes Obes*. 2011;18(2):139 - 43.
10. Roden M, Price TB, Perseghin G, et al. Mechanism of Free Fatty Acid–induced Insulin Resistance in Humans. *J Clin Invest*. 1996;97(12):2859 - 65.
11. Karpe F, Dickmann JR, Frayn KN. Fatty Acids, Obesity, and Insulin Resistance: Time for a Reevaluation. *Diabetes*. 2011;60(10):2441 - 9
12. Mingrone G, Panunzi S, De Gaetano A, et al. Bariatric Surgery versus Conventional Medical Therapy for Type 2 Diabetes. *N Engl J Med*. 2012;366:1577 - 85.
13. Buchwald H, Estok R, Fahrbach K, et al. Weight and type 2 diabetes after bariatric surgery: systematic review and meta-analysis. *Am J Med*. 2009;122(3):248-56 e5.
14. Rubino F, Schauer PR, Kaplan LM, et al. Metabolic surgery to treat type 2 diabetes: clinical outcomes and mechanisms of action. *Annu Rev Med*. 2010;61:393-411.
15. Dixon JB. Obesity and diabetes: the impact of bariatric surgery on type-2 diabetes. *World J Surg*. 2009;33(10):2014-21.
16. Sjöström L, Lindroos A-K, Peltonen M, et al. Lifestyle, Diabetes, and Cardiovascular Risk Factors 10 Years after Bariatric Surgery. *N Engl J Med*. 2004;351:2683 - 93.
17. Dixon JB, le Roux CW, Rubino F, et al. Bariatric surgery for type 2 diabetes. *The Lancet*. 2012;379(9833):2300-11.
18. Schauer PR, Bhatt DL, Kirwan JP, et al. Bariatric Surgery versus Intensive Medical Therapy for Diabetes - 5-Year Outcomes. *N Engl J Med*. 2017;376(7):641-51.
19. Liang Z, Wu Q, Chen B, et al. Effect of laparoscopic Roux-en-Y gastric bypass surgery on type 2 diabetes mellitus with hypertension: a randomized controlled trial. *Diabetes Res Clin Pract*. 2013;101(1):50-6.
20. Gumbs AA, Modlin IM, Ballantyne GH. Changes in Insulin Resistance Following Bariatric Surgery: Role of Caloric Restriction and Weight Loss. *Obes Surg*. 2005;15(4):462 - 73.
21. Rubino F, Forgione A, Cummings DE, et al. The mechanism of diabetes control after gastrointestinal bypass surgery reveals a role of the proximal small intestine in the pathophysiology of type 2 diabetes. *Ann Surg*. 2006;244(5):741-9.
22. Cummings DE. Endocrine mechanisms mediating remission of diabetes after gastric bypass surgery. *Int J Obes (Lond)*. 2009;33 Suppl 1:S33-40.
23. Pacheco D, de Luis DA, Romero A, et al. The effects of duodenal-jejunal exclusion on hormonal regulation of glucose metabolism in Goto-Kakizaki rats. *Am J Surg*. 2007;194(2):221-4.
24. Cohen RV, Schiavon CA, Pinheiro JS, et al. Duodenal-jejunal bypass for the treatment of type 2 diabetes in patients with body mass index of 22-34 kg/m2: a report of 2 cases. *Surg Obes Relat Dis*. 2007;3(2):195-7.

For peer review only

25. Thaler JP, Cummings DE. Minireview: Hormonal and metabolic mechanisms of diabetes remission after gastrointestinal surgery. *Endocrinology*. 2009;150(6):2518-25.
26. Cummings DE, Overduin J, Shannon MH, et al. Hormonal mechanisms of weight loss and diabetes resolution after bariatric surgery. *Surg Obes Relat Dis*. 2005;1(3):358-68.
27. Shuang J, Zhang Y, Ma L, et al. Relief of diabetes by duodenal-jejunal bypass sleeve implantation in the high-fat diet and streptozotocin-induced diabetic rat model is associated with an increase in GLP-1 levels and the number of GLP-1-positive cells. *Exp Ther Med*. 2015;10(4):1355-63.
28. Knop FK. Resolution of type 2 diabetes following gastric bypass surgery: involvement of gut-derived glucagon and glucagonotropic signalling? *Diabetologia*. 2009;52(11):2270-6.
29. de Jonge C, Rensen SS, Verdam FJ, et al. Impact of Duodenal-Jejunal Exclusion on Satiety Hormones. *Obes Surg*. 2016;26(3):672-8.
30. de Jonge C, Rensen SS, Verdam FJ, et al. Endoscopic duodenal-jejunal bypass liner rapidly improves type 2 diabetes. *Obes Surg*. 2013;23(9):1354-60.
31. Munoz R, Escalona A. Duodenal-jejunal bypass liner to treat type 2 diabetes mellitus in morbidly obese patients. *Curr Cardiol Rep*. 2014;16(3):454.
32. Scholtz S, Miras AD, Chhina N, et al. Obese patients after gastric bypass surgery have lower brain-hedonic responses to food than after gastric banding. *Gut*. 2014;63(6):891-902.
33. Miras AD, Jackson RN, Jackson SN, et al. Gastric bypass surgery for obesity decreases the reward value of a sweet-fat stimulus as assessed in a progressive ratio task. *Am J Clin Nutr*. 2012;96(3):467-73.
34. Goldstone A, Miras A, Scholtz S, et al. Link Between Increased Satiety Gut Hormones and Reduced Food Reward After Gastric Bypass Surgery for Obesity. *J Clin Endocrinol Metab*. 2016;101(2):599 - 609.
35. Ochner CN, Stice E, Hutchins E, et al. Relation between changes in neural responsivity and reductions in desire to eat high-calorie foods following gastric bypass surgery. *Neuroscience*. 2012;209:128-35.
36. Milone L, Gagner M, Ueda K, et al. Effect of a Polyethylene Endoluminal Duodeno- Jejunal Tube (EDJT) on Weight Gain: A Feasibility Study in a Porcine Model. *Obes Surg*. 2006;16:620 - 6.
37. Koehestanie P, de Jonge C, Berends FJ, et al. The effect of the endoscopic duodenal-jejunal bypass liner on obesity and type 2 diabetes mellitus, a multicenter randomized controlled trial. *Ann Surg*. 2014;260(6):984-92.
38. Schouten R, Rijs CS, Bouvy ND, et al. A multicenter, randomized efficacy study of the EndoBarrier Gastrointestinal Liner for presurgical weight loss prior to bariatric surgery. *Ann Surg*. 2010;251(2):236-43.
39. Gersin KS, Rothstein RI, Rosenthal RJ, et al. Open-label, sham-controlled trial of an endoscopic duodenojejunal bypass liner for preoperative weight loss in bariatric surgery candidates. *Gastrointest Endosc*. 2010;71(6):976-82.
40. Tarnoff M, Rodriguez L, Escalona A, et al. Open label, prospective, randomized controlled trial of an endoscopic duodenal-jejunal bypass sleeve versus low calorie diet for pre-operative weight loss in bariatric surgery. *Surg Endosc*. 2009;23(3):650-6.
41. Rodriguez L, Reyes E, Fagalde P, et al. Pilot clinical study of an endoscopic, removable duodenal-jejunal bypass liner for the treatment of type 2 diabetes. *Diabetes Technol Ther*. 2009;11(11):725 - 32.
42. Rodriguez-Grunert L, Galvao Neto MP, Alamo M, et al. First human experience with endoscopically delivered and retrieved duodenal-jejunal bypass sleeve. *Surg Obes Relat Dis*. 2008;4(1):55-9.
43. Escalona A, Yanez R, Pimentel F, et al. Initial human experience with restrictive duodenal-jejunal bypass liner for treatment of morbid obesity. *Surg Obes Relat Dis*. 2010;6(2):126-31.
44. de Moura EGH, Martins BC, Lopes GS, et al. Metabolic improvements in obese type 2 diabetes subjects implanted for 1 year with an endoscopically deployed duodenal-jejunal bypass liner. *Diabetes Technol Ther*. 2012;14(2):183 - 9.
45. Escalona A, Pimentel F, Sharp A, et al. Weight loss and metabolic improvement in morbidly obese subjects implanted for 1 year with an endoscopic duodenal-jejunal bypass liner. *Ann Surg*. 2012;255(6):1080-5.

For peer review only

46. Cohen R, le Roux CW, Papamargaritis D, et al. Role of proximal gut exclusion from food on glucose homeostasis in patients with Type 2 diabetes. *Diabet Med*. 2013;30(12):1482-6.
47. Koehestanie P, Betzel B, Dogan K, et al. The feasibility of delivering a duodenal-jejunal bypass liner (EndoBarrier) endoscopically with patients under conscious sedation. *Surg Endosc*. 2014;28(1):325-30.
48. Munoz R, Dominguez A, Munoz F, et al. Baseline glycated hemoglobin levels are associated with duodenal-jejunal bypass liner-induced weight loss in obese patients. *Surg Endosc*. 2014;28(4):1056-62.
49. Force ABET, Committee AT, Abu Dayyeh BK, et al. ASGE Bariatric Endoscopy Task Force systematic review and meta-analysis assessing the ASGE PIVI thresholds for adopting endoscopic bariatric therapies. *Gastrointest Endosc*. 2015;82(3):425-38 e5.
50. Rohde U, Hedback N, Gluud LL, et al. Effect of the EndoBarrier Gastrointestinal Liner on obesity and type 2 diabetes: a systematic review and meta-analysis. *Diabetes Obes Metab*. 2016;18(3):300-5.
51. Dixon JB, Zimmet P, Alberti K, et al. Bariatric Surgery: an IDF statement for obese Type 2 diabetes. *Diabet Med*. 2011;28:628 - 42.
52. Association AD. *Standards of Medical Care in Diabetes - 2016.* 2016.
53. Inzucchi S, Bergenstal R, Buse J, et al. Management of Hyperglycemia in Type 2 Diabetes: A Patient-Centered Approach. *Diabetes Care*. 2012;35(6):1364-79.
54. Craig CL, Marshall AL, Sjostrom M, et al. International physical activity questionnaire: 12-country reliability and validity. *Med Sci Sports Exerc*. 2003;35(8):1381-95.
55. Clarke P, Gray A, Holman R. Estimating Utility Values for Health States of Type 2 Diabetic Patients Using the EQ-5D (UKPDS 62). *Med Decis Making*. 2002;22(4):340 - 9.
56. Williams JG, Alam MF, Alrubaiy L, et al. Infliximab versus ciclosporin for steroid-resistant acute severe ulcerative colitis (CONSTRUCT): a mixed methods, open-label, pragmatic randomised trial. *The Lancet Gastroenterology & Hepatology*. 2016;1(1):15-24.
57. Chernyak N, Ernsting C, Icks A. Pre-test of questions on health-related resource use and expenditure, using behaviour coding and cognitive interviewing techniques. *BMC Health Serv Res*. 2012;12:303.
58. Petersen E, Zimine I, Ho Y, et al. Non-invasive measurement of perfusion: a critical review of arterial spin labelling techniques. *Br J Radiol*. 2006;79(944):688 - 701.
59. Paiva FF, Tannus A, Silva AC. Measurement of cerebral perfusion territories using arterial spin labelling. *NMR Biomed*. 2007;20(7):633-42.
60. Damoiseaux JS, Rombouts SA, Barkhof F, et al. Consistent resting-state networks across healthy subjects. *Proc Natl Acad Sci U S A*. 2006;103(37):13848-53.
61. Goldstone A, Prechtl de Hernandez C, Beaver J, et al. Fasting biases brain reward systems towards high-calorie foods. *Eur J Neurosci*. 2009;30(8):1625 - 35.
62. Byrne CS, Chambers ES, Alhabeeb H, et al. Increased colonic propionate reduces anticipatory reward responses in the human striatum to high-energy foods. *Am J Clin Nutr*. 2016;104(1):5-14.
63. Goldstone AP, Prechtl CG, Scholtz S, et al. Ghrelin mimics fasting to enhance human hedonic, orbitofrontal cortex, and hippocampal responses to food. *Am J Clin Nutr*. 2014;99(6):1319-30.
64. Knutson B, Adams C, Fong G, et al. Anticipation of Increasing Monetary Reward Selectively Recruits Nucleus Accumbens. *J Neurosci*. 2001;21(16):RC159.
65. Knutson B, Westdorp A, Kaiser E, et al. FMRI visualization of brain activity during a monetary incentive delay task. *Neuroimage*. 2000;12(1):20-7.
66. McGonigle J, Murphy A, Paterson LM, et al. The ICCAM platform study: An experimental medicine platform for evaluating new drugs for relapse prevention in addiction. Part B: fMRI description. *J Psychopharmacol*. 2017;31(1):3-16.
67. Perry JL, Carroll ME. The role of impulsive behavior in drug abuse. *Psychopharmacology (Berl)*. 2008;200(1):1-26.
68. Horn NR, Dolan M, Elliott R, et al. Response inhibition and impulsivity: an fMRI study. *Neuropsychologia*. 2003;41(14):1959-66.
69. Li CS, Yan P, Chao HH, et al. Error-specific medial cortical and subcortical activity during the stop signal task: a functional magnetic resonance imaging study. *Neuroscience*. 2008;155(4):1142-51.

For peer review only

70. Kirby K, Maraković N. Delay-discounting probabilistic rewards: Rates decrease as amounts increase. *Psychonomic Bulletin & Review*. 1996;3(1):100 - 4.

71. Finlayson G, King N, Blundell J. The role of implicit wanting in relation to explicit liking and wanting for food: implications for appetite control. *Appetite*. 2008;50(1):120-7.

72. Bueter M, Miras AD, Chichger H, et al. Alterations of sucrose preference after Roux-en-Y gastric bypass. *Physiol Behav*. 2011;104(5):709-21.

73. Gæde P, Lund-Andersen H, Parving H, et al. Effect of a Multifactorial Intervention on Mortality in Type 2 Diabetes. *N Engl J Med*. 2008;358:580 - 91.

74. Devlin N, Shah K, Feng Y, et al. Economics OoH. *Valuing Health-Related Quality of Life: An EQ-5D-5L Value Set for England.* . 2016.

75. Philips Z, Ginnelly L, Sculpher M, et al. Review of guidelines for good practice in decision-analytic modelling in health technology assessment. *Health technology assessment*. 2004;8(36):iii-iv, ix-xi, 1-158.

76. Caro JJ, Briggs AH, Siebert U, et al. Modeling good research practices--overview: a report of the ISPOR-SMDM Modeling Good Research Practices Task Force-1. *Medical decision making : an international journal of the Society for Medical Decision Making*. 2012;32(5):667-77.

For peer review only

Figure Legends

Figure 1. EndoBarrier® Gastrointestinal Bypass Liner

Figure 2. Study interventions and follow-up schedule

For peer review only

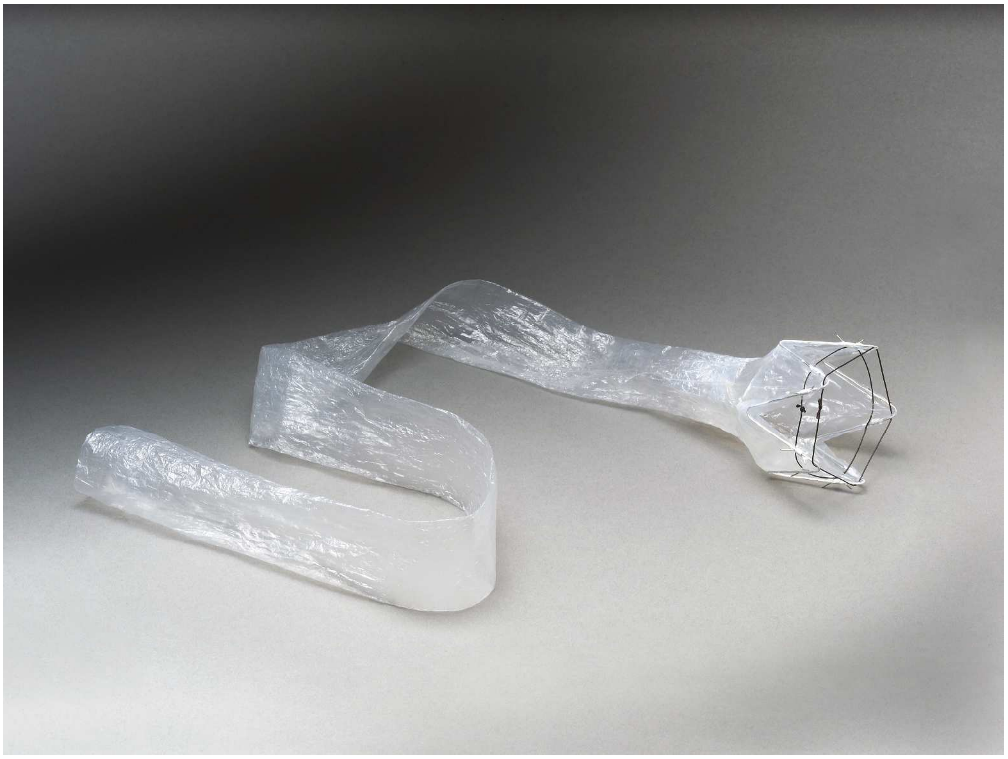

Figure 1. EndoBarrier Gastrointestinal Bypass Liner

344x258mm (300 x 300 DPI)

For peer review only

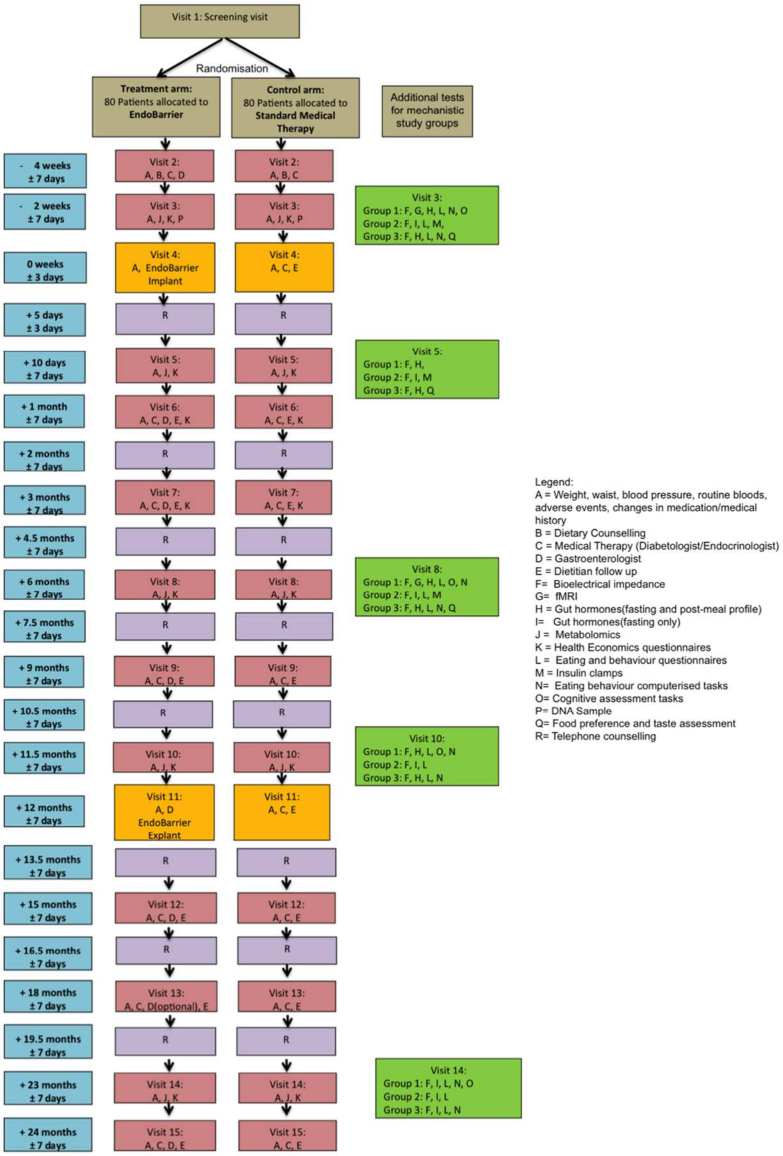

Figure 2. Study interventions and follow-up schedule

95x138mm (300 x 300 DPI)

For peer review only - http://bmjopen.bmj.com/site/about/guidelines.xhtml

STANDARD PROTOCOL ITEMS: RECOMMENDATIONS FOR INTERVENTIONAL TRIALS

SPIRIT 2013 Checklist: Recommended items to address in a clinical trial protocol and related documents*

Section/item	Item No	Description	Addressed on page number
**Administrative information**			
Title	1	Descriptive title identifying the study design, population, interventions, and, if applicable, trial acronym	______1_______
Trial registration	2a	Trial identifier and registry name. If not yet registered, name of intended registry	______4_______
	2b	All items from the World Health Organization Trial Registration Data Set	https://clinicaltrials.gov/ct2/show/record/NCT02459561
Protocol version	3	Date and version identifier	______8_______
Funding	4	Sources and types of financial, material, and other support	______5______
Roles and responsibilities	5a	Names, affiliations, and roles of protocol contributors	_____1-3______
	5b	Name and contact information for the trial sponsor	______5______
	5c	Role of study sponsor and funders, if any, in study design; collection, management, analysis, and interpretation of data; writing of the report; and the decision to submit the report for publication, including whether they will have ultimate authority over any of these activities	______5_______

For peer review only

For peer review only - http://bmjopen.bmj.com/site/about/guidelines.xhtml

	5d	Composition, roles, and responsibilities of the coordinating centre, steering committee, endpoint adjudication committee, data management team, and other individuals or groups overseeing the trial, if applicable (see Item 21a for data monitoring committee)	______8_______
**Introduction**			
Background and rationale	6a	Description of research question and justification for undertaking the trial, including summary of relevant studies (published and unpublished) examining benefits and harms for each intervention	____9-10______
	6b	Explanation for choice of comparators	___ 9-12______
Objectives	7	Specific objectives or hypotheses	____10-11______
Trial design	8	Description of trial design including type of trial (eg, parallel group, crossover, factorial, single group), allocation ratio, and framework (eg, superiority, equivalence, noninferiority, exploratory)	_____11________
**Methods: Participants, interventions, and outcomes**			
Study setting	9	Description of study settings (eg, community clinic, academic hospital) and list of countries where data will be collected. Reference to where list of study sites can be obtained	__11, 16-17____
Eligibility criteria	10	Inclusion and exclusion criteria for participants. If applicable, eligibility criteria for study centres and individuals who will perform the interventions (eg, surgeons, psychotherapists)	_____16_______
Interventions	11a	Interventions for each group with sufficient detail to allow replication, including how and when they will be administered	___17-19______
	11b	Criteria for discontinuing or modifying allocated interventions for a given trial participant (eg, drug dose change in response to harms, participant request, or improving/worsening disease)	____17-19_____ Main protocol Section 7
	11c	Strategies to improve adherence to intervention protocols, and any procedures for monitoring adherence (eg, drug tablet return, laboratory tests)	_____27______

For peer review only - http://bmjopen.bmj.com/site/about/guidelines.xhtml

	11d	Relevant concomitant care and interventions that are permitted or prohibited during the trial	_____16______
Outcomes	12	Primary, secondary, and other outcomes, including the specific measurement variable (eg, systolic blood pressure), analysis metric (eg, change from baseline, final value, time to event), method of aggregation (eg, median, proportion), and time point for each outcome. Explanation of the clinical relevance of chosen efficacy and harm outcomes is strongly recommended	__10-11, 19-23___
Participant timeline	13	Time schedule of enrolment, interventions (including any run-ins and washouts), assessments, and visits for participants. A schematic diagram is highly recommended (see Figure)	__13-15, 29-30__
Sample size	14	Estimated number of participants needed to achieve study objectives and how it was determined, including clinical and statistical assumptions supporting any sample size calculations	_____23_______
Recruitment	15	Strategies for achieving adequate participant enrolment to reach target sample size	____16-17____
**Methods: Assignment of interventions (for controlled trials)**			
Allocation:			
Sequence generation	16a	Method of generating the allocation sequence (eg, computer-generated random numbers), and list of any factors for stratification. To reduce predictability of a random sequence, details of any planned restriction (eg, blocking) should be provided in a separate document that is unavailable to those who enrol participants or assign interventions	_____17______
Allocation concealment mechanism	16b	Mechanism of implementing the allocation sequence (eg, central telephone; sequentially numbered, opaque, sealed envelopes), describing any steps to conceal the sequence until interventions are assigned	_____17______
Implementation	16c	Who will generate the allocation sequence, who will enrol participants, and who will assign participants to interventions	_____17______
Blinding (masking)	17a	Who will be blinded after assignment to interventions (eg, trial participants, care providers, outcome assessors, data analysts), and how	_____N/A______
	17b	If blinded, circumstances under which unblinding is permissible, and procedure for revealing a participant's allocated intervention during the trial	_____N/A______

For peer review only - http://bmjopen.bmj.com/site/about/guidelines.xhtml

Methods: Data collection, management, and analysis

Data collection methods	18a	Plans for assessment and collection of outcome, baseline, and other trial data, including any related processes to promote data quality (eg, duplicate measurements, training of assessors) and a description of study instruments (eg, questionnaires, laboratory tests) along with their reliability and validity, if known. Reference to where data collection forms can be found, if not in the protocol	___19-23____
	18b	Plans to promote participant retention and complete follow-up, including list of any outcome data to be collected for participants who discontinue or deviate from intervention protocols	___23-24, 27____
Data management	19	Plans for data entry, coding, security, and storage, including any related processes to promote data quality (eg, double data entry; range checks for data values). Reference to where details of data management procedures can be found, if not in the protocol	____19, 27_____ Main protocol Section 9, 11.9 & 12
Statistical methods	20a	Statistical methods for analysing primary and secondary outcomes. Reference to where other details of the statistical analysis plan can be found, if not in the protocol	___23-25____
	20b	Methods for any additional analyses (eg, subgroup and adjusted analyses)	____23-25___
	20c	Definition of analysis population relating to protocol non-adherence (eg, as randomised analysis), and any statistical methods to handle missing data (eg, multiple imputation)	___23-25____

Methods: Monitoring

Data monitoring	21a	Composition of data monitoring committee (DMC); summary of its role and reporting structure; statement of whether it is independent from the sponsor and competing interests; and reference to where further details about its charter can be found, if not in the protocol. Alternatively, an explanation of why a DMC is not needed	_____8_____ Main protocol Section 12
	21b	Description of any interim analyses and stopping guidelines, including who will have access to these interim results and make the final decision to terminate the trial	____N/A______
Harms	22	Plans for collecting, assessing, reporting, and managing solicited and spontaneously reported adverse events and other unintended effects of trial interventions or trial conduct	____19_____ Main protocol section 6

For peer review only - http://bmjopen.bmj.com/site/about/guidelines.xhtml

Auditing	23	Frequency and procedures for auditing trial conduct, if any, and whether the process will be independent from investigators and the sponsor	____8, 27____ Main protocol section 12
**Ethics and dissemination**			
Research ethics approval	24	Plans for seeking research ethics committee/institutional review board (REC/IRB) approval	_____N/A_____
Protocol amendments	25	Plans for communicating important protocol modifications (eg, changes to eligibility criteria, outcomes, analyses) to relevant parties (eg, investigators, REC/IRBs, trial participants, trial registries, journals, regulators)	____N/A_____
Consent or assent	26a	Who will obtain informed consent or assent from potential trial participants or authorised surrogates, and how (see Item 32)	_____17_____ Main protocol section 11.5
	26b	Additional consent provisions for collection and use of participant data and biological specimens in ancillary studies, if applicable	_____17_____ Main protocol section 11.5
Confidentiality	27	How personal information about potential and enrolled participants will be collected, shared, and maintained in order to protect confidentiality before, during, and after the trial	_____5______ Main protocol section 11
Declaration of interests	28	Financial and other competing interests for principal investigators for the overall trial and each study site	_____5-6______
Access to data	29	Statement of who will have access to the final trial dataset, and disclosure of contractual agreements that limit such access for investigators	_____5______ Main protocol section 12
Ancillary and post-trial care	30	Provisions, if any, for ancillary and post-trial care, and for compensation to those who suffer harm from trial participation	____N/A_____

For peer review only - http://bmjopen.bmj.com/site/about/guidelines.xhtml

Dissemination policy	31a	Plans for investigators and sponsor to communicate trial results to participants, healthcare professionals, the public, and other relevant groups (eg, via publication, reporting in results databases, or other data sharing arrangements), including any publication restrictions	____N/A_____ Main protocol section 12.8
	31b	Authorship eligibility guidelines and any intended use of professional writers	____N/A______
	31c	Plans, if any, for granting public access to the full protocol, participant-level dataset, and statistical code	____N/A_____
**Appendices**			
Informed consent materials	32	Model consent form and other related documentation given to participants and authorised surrogates	_____N/A_____
Biological specimens	33	Plans for collection, laboratory evaluation, and storage of biological specimens for genetic or molecular analysis in the current trial and for future use in ancillary studies, if applicable	_____N/A_____

*It is strongly recommended that this checklist be read in conjunction with the SPIRIT 2013 Explanation & Elaboration for important clarification on the items. Amendments to the protocol should be tracked and dated. The SPIRIT checklist is copyrighted by the SPIRIT Group under the Creative Commons "Attribution-NonCommercial-NoDerivs 3.0 Unported" license.

For peer review only - http://bmjopen.bmj.com/site/about/guidelines.xhtml
